# Antifungal activity of volatile organic compounds produced by *Bacillus subtilis* GB519 against blast pathogen *Magnaporthe oryzae* in rice

**DOI:** 10.3389/fmicb.2026.1757473

**Published:** 2026-03-11

**Authors:** Feng Zhu, Jichun Wang, Chengli Tian, Yuting Xu, Yingshuang Gao, Xintong Zhang, Peng Gao, Qianfu Su

**Affiliations:** 1 Key Laboratory of Integrated Pest Management on Crops in Northeast, Ministry of Agriculture and Rural Affairs, Institute of Plant Protection, Jilin Academy of Agricultural Sciences (Northeast Agricultural Research Center of China), Changchun, China; 2 Institute of Agricultural Resource and Environment, Jilin Academy of Agricultural Sciences (Northeast Agricultural Research Center of China), Changchun, China; 3 College of Agriculture, Yanbian University, Yanji, China; 4 College of Plant Protection, Jilin Agricultural University, Changchun, China

**Keywords:** antifungal activity, *Bacillus subtili*s GB519, gas chromatography-mass spectrometer, *Magnaporthe oryzae*, volatile organic compounds

## Abstract

The use of microbial-derived volatile organic compounds (VOCs) for the biocontrol of plant diseases has attracted increasing attention. This study, using a two-compartment petri dish assay, investigated the antifungal activity of VOCs produced by *Bacillus subtilis* GB519 against *Magnaporthe oryzae*, the causal pathogen of rice blast disease. The results revealed that the VOCs emitted by strain GB519 significantly inhibited the mycelial growth and biomass accumulation of *M. oryzae* by up to 30.7% and 20.9%, respectively. Exposure to GB519 VOCs disrupted hyphal morphology, increased cell membrane permeability, and induced substantial leakage of intracellular contents, evidenced by significant increases in extracellular pyruvate (59.9%), alkaline phosphatase activity (33.7%), nucleic acids (249.4%), and electrical conductivity (31.8%). Furthermore, VOCs markedly reduced the activity and gene expression of superoxide dismutase (SOD) and catalase (CAT), leading to ROS and subsequent severe hyphal damage. The VOCs also downregulated the expression level of melanin biosynthesis gene *BUF1* in *M. oryzae*, which correlated with suppressed lesion development on rice leaves. Additionally, GB519 VOCs exhibited broad-spectrum antagonism against multiple soil-borne pathogens. SPME-GC-MS analysis of the volatilome identified 12 major VOCs produced by strain GB519. Collectively, these results indicate that GB519 VOCs trigger oxidative stress and mitochondrial dysfunction in *M. oryzae*, thereby exerting potent antifungal effects. These findings provide new insights into the application of microbial VOCs and their bioactive constituents in the sustainable management of blast disease in rice.

## 1 Introduction

Rice blast, caused by the fungal pathogen *Magnaporthe oryzae*, is one of the most devastating diseases to affect global rice production, leading to severe yield losses (Dean et al., 2005). Traditionally, the use of chemical fungicides has served as the primary means to control this disease. However, their overuse has raised serious concerns, including environmental degradation, water contamination, adverse effects on non-target organisms, and the increasing development of resistant pathogen strains (Du et al., 2011). There is a growing consensus on the urgent need for sustainable and eco-friendly alternatives to manage blast disease in rice. Such strategies are critical not only for safeguarding crop health but also for promoting biodiversity, mitigating ecological disruption, and enhancing resilience to climate change and emerging pathogens (Asibi et al., 2019). Ultimately, developing effective, sustainable solutions for blast management is essential to balancing agricultural productivity with environmental conservation and ensuring long-term food security in the world (Younas et al., 2023).

A promising alternative is the use of beneficial bacteria such as *Bacillus*, which offers a sustainable and eco-friendly solution to disease control in crop production. As a widely used biocontrol agent, *Bacillus* inhibits the growth of pathogenic microorganisms through multiple mechanisms, including competition for nutrients and space, root colonization, and the production of diverse antimicrobial substances, thus showing significant potential for disease control (Naqvi et al., 2025). Among these, volatile organic compounds (VOCs) have attracted considerable attention. The low-molecular-weight VOCs encompass a wide range of chemical classes, including aldehydes, alcohols, ketones, terpenes, esters, hydrocarbons, nitrogen-and sulfur-containing compounds and organic acids (Calvo et al., 2020; Chaouachi et al., 2021). VOCs are easily diffusible, possess broad-spectrum antimicrobial activity, and pose low ecological risks, making them ideal candidates for biopesticide development (Sharifi and Ryu, 2018; Dhouib et al., 2019; Cerimi et al., 2025). Previous studies have demonstrated that microbial VOCs can inhibit mycelial growth and spore germination of various phytopathogenic fungi, underscoring their great potential in plant disease control (Yuan et al., 2012; Sharifah et al., 2019; Wu et al., 2019).

However, while the broad-spectrum antifungal effects of *Bacillus* VOCs are recognized, the precise mechanism by which GB519 VOCs disrupt the physiological machinery of *M. oryzae* remains less clear. In particular, it is unknown whether and how these VOCs transcriptionally modulate the fungal antioxidant defense system and whether such modulation is causally linked to the induction of mitochondrial dysfunction-a critical aspect of VOC-mediated fungal inhibition that remains to be fully elucidated.

In this study, we comprehensively examined the antifungal effects of VOCs produced by *B. subtilis* GB519 against *M. oryzae*, a causative agent of blast in rice. Specifically, we aimed to: (i) evaluate the antifungal potency of GB519-derived VOCs; (ii) elucidate their effects on fungal morphology and cellular physiology; (iii) assess their effects on reactive oxygen species (ROS)-related enzyme activities and gene expression; (iv) investigate their impact on pathogenicity-related genes; (v) identify key antifungal VOCs through volatilome profiling.

The findings from our examinations are expected to provide fundamental knowledge for developing VOC-based biocontrol strategies for the sustainable management of blast disease in rice. Moreover, this research supports the broader goal of implementing environmentally responsible agricultural practices to enhance ecosystem resilience and long-term crop productivity.

## 2 Materials and methods

### 2.1 Microorganisms and cultural conditions

The strain *B. subtilis* GB519 was previously isolated and characterized by Zhu F. et al. (2021) and is currently stored at the China General Microbiological Culture Collection Center (CGMCC No. 15115). The strain was activated and cultured in LB liquid medium at 30°C for 48 h.

The fungal pathogens used in this study included *M. oryzae* (Wang et al., 2024), *Fusarium fujikuroi* (Ou et al., 2024), *F. graminearum*, *F. oxysporum*, *Rhizoctonia solani*, and *Sclerotium rolfsii* (Zhu F. et al., 2021). These pathogens were all identified and stored in our laboratory. Prior to use, all the pathogens were cultured on potato dextrose agar (PDA) medium at 25°C for 7 days to ensure their viability.

### 2.2 Antifungal activity of GB519 VOCs on the growth and morphology of M. oryzae

The antifungal effects of the VOCs from *B. subtilis* GB519 were evaluated using a standardized two-compartment (double petri dish) assay, as described by Zou et al. (2022). To ensure reproducibility and minimize confounding factors, key conditions were strictly controlled: 1) a consistent VOC source was established by evenly spreading 100 μL of GB519 broth (10^8^ cfu/mL) onto LB agar in one compartment; 2) a 5-mm mycelial plug of *M. oryzae* was inoculated onto a sterile cellophane-covered PDA plate in the opposing compartment; 3) the two plates (90 mm diameter) were immediately paired and sealed together with three layers of Parafilm to create a shared, confined headspace; and 4) all sets were incubated at 28°C in darkness. A negative control pair, in which the LB plate contained no bacteria, was included under identical conditions. The incubation period was fixed until the control colonies reached the plate edge, ensuring assessment over a comparable growth phase. At this endpoint, the colony diameter of *M. oryzae* was measured, and the mycelial biomass was collected to determine fresh weight. Inhibition rates were calculated as described by Gao et al. (2017). Each treatment was performed in triplicate, and the entire experiment was independently repeated twice. For morphological assessment of M. oryzae in response to GB519 VOCs, hyphae sampled from the colony margin were stained with lactophenol cotton blue, and observed under a Zeiss Axio Imager A1 microscope (Zeiss, Jena, Germany) following established protocols (Medina-Romero et al., 2017; Zhang et al., 2019).

### 2.3 Effects of GB519 VOCs on the activities of antioxidant enzymes in M. oryzae

The mycelia treated with GB519 VOCs were harvested, frozen in liquid nitrogen, and stored at −70°C for analysis. The activities of superoxide dismutase (SOD), catalase (CAT), and total antioxidant capacity (TAOC) were quantified using commercial assay kits (Leagene Biotechnology Co., Ltd., Beijing, China). Total protein content was determined using the BCA protein assay kit (Leagene Biotechnology Co., Ltd., Beijing, China). Pyruvate levels were measured using a micro pyruvate assay kit, following the manufacturer’s instructions. Each treatment included three biological replicates, and the entire experiment was independently repeated twice.

### 2.4 Effects of GB519 VOCs on cell integrity and membrane permeability in M. oryzae

To assess cell integrity, the mycelia treated with GB519 VOCs were stained using the Hoechst 33258/PI apoptosis assay kit (Leagene), and then observed under a Leica TCS SP8 laser confocal microscope (Leica Microsystems, Mannheim, Germany), following the protocol outlined by Harikrishnan et al. (2014) and Zhang et al. (2021). Membrane permeability was assessed by measuring electric conductivity and nucleic acids leakage. Fresh mycelia (0.5 g) were suspended in 5 mL of double-distilled water, shaken for 1 h, and centrifuged at 12,000 × g for 10 min. The resulting supernatant was used to determine cellular leakage using a conductivity meter (FE30, Mettler Toledo Co., Ltd., China) and a multifunctional microplate reader (SpectraMax i3x, Molecular Devices, LLC., Austria), respectively. Alkaline phosphatase activity (AKP) in crude cellular extracts was measured using a commercial kit (Leagene) with the manufacturer’s instructions. Results were normalized to protein content and expressed as U/L of protein. All experiments were repeated three times, and the entire experiment was independently repeated twice.

### 2.5 Measurement of MMP and ROS in *M. oryzae* mycelia treated with GB519 VOCs

Mitochondrial membrane potential (MMP) was measured using a mitochondria-specific probe JC-1, following the procedure described by Li et al. (2019). The mycelia were treated with 5 mmoL/L H_2_O_2_ and 5 mmoL/L ascorbate for 6 h, which were used as the positive and negative control treatments, respectively. The treated mycelia were washed, resuspended in JC-1 buffer and observed under a Leica TCS SP8 laser confocal microscope (Leica Microsystems, Mannheim, Germany). Intracellular ROS accumulation in M. oryzae mycelia treated with GB519 VOCs was assessed using the fluorescent probe DCFH-DA (10 μM; Meilunbio, Dalian, China), as described by Lee et al. (2020). The mycelia of M. oryzae treated with GB519 were incubated at 37°C for 30 min in darkness, washed with PBS buffer, and visualized under a Leica TCS SP8 laser confocal microscope (Leica Microsystems, Mannheim, Germany). Each treatment included three biological replicates, and the entire experiment was independently repeated twice.

### 2.6 Leaf protection test by GB519 VOCs

Wound-free leaves of rice were sampled and sterilized by soaking in 75% alcohol for 2 min and thoroughly rinsed with distilled water. A 2-mm mycelium disc of M. oryzae was then placed on each leaf. Both ends of the leaf were wrapped with sterile cotton balls moistened with 6-BA (0.5 μg/mL) before transferring to a 180-mm petri dish, along with a 90-mm LB petri dish containing GB519 broth. Each large petri dish contained two fungal treatments (non-infected leaves, leaves infected with *M. oryzae*), and each small one contained either the GB519 strain on solid LB medium or no bacterium as a control. The large petri dishes were lined with two sheets of moistened and sterilized paper, sealed with parafilm to prevent leakage of volatile substances, and incubated at 25°C. Lesion diameter due to M. oryzae infection on each leaf was measured 7 days after inoculation. The experiment was repeated three times, and each treatment had two replicates.

### 2.7 Broad-spectrum antifungal activities of GB519 VOCs

The inhibitory effects of GB519 VOCs on several other pathogens were assessed using the double petri dish method described in section 2.2, where the controls consisted of LBA plates without GB519. The experiment was repeated three times, and each treatment had two replicates.

### 2.8 VOC identification via SPME-GC-MS

Solid phase microextraction-gas chromatography-mass spectrometer (SPME-GC- MS) was used to quantify VOCs. GB519 was cultured in headspace vials (5 mL/20 mL) containing autoclaved LB medium at 30°C for 7 d in darkness, where the vials without GB519 served as controls. The VOCs from GB519 were extracted using a Carboxen/DVB/PDMS SPME fiber (2 cm, 50/30 μm) from Agilent Technologies (United States), which was preconditioned at 250°C for 30 min before exposing to the headspace of culture vials for 60 min. The collected VOCs were analyzed using a Shimadzu GC-2010 high-performance gas chromatograph coupled with a Shimadzu GC-MS-QP 2010 Ultra mass spectrometer. The gas was chromatographed using a nonpolar capillary column (Rtx-5MS, 30 m × 0.25 mm × 0.25 μm), where the temperature was programmed at 40°C for 5 min, ramped up to 250°C at 5°C/min, and held for 15 min. Compounds were identified by comparing the mass spectrogram of each peak in the total ion chromatography with the National Institute of Standards and Technology (NIST) database (2017).

### 2.9 Antifungal activity of pure VOCs

Based on the identified VOCs, 12 commercial standard compounds were purchased (Table 1). The antifungal activity of these compounds was assessed using the double-sealed petri dish method (Zou et al., 2022), which was identical to the method used for the GB519 VOCs as described in section 2.2. A 5-mm mycelium plug of *M. oryzae* was inoculated on a PDA plate layered with cellophane, and a 15-mm diameter cellulose disc was placed at the base of another plate, impregnated with 100 μL of pure compounds. Both plates were sealed together with parafilm and incubated at 28°C. A third plate supplemented with ethanol or sterile water served as a negative control. Colony diameters of M. oryzae were measured once the colony reached the edge of the control plate.

**TABLE 1:** Volatile organic compounds from *Bacillus subtilis* GB519 were identified using GC-MS.

Retention time	Identified compound	Molecular formula	CAS#	Antifungal activity (%)
12.991	2-Butanone, 3-hydroxy-	C_4_H_8_O_2_	513–86–0	6.92
13.800	Pyrazine, 2,6-dimethyl-	C_6_H_8_N_2_	108–50–9	4.61
15.082	2-Nonanone	C_9_H_18_O	821–55–6	6.16
15.937	Pyrazine, 3-ethyl-2,5-dimethyl-	C_8_H_12_N_2_	13,360–65–1	5.37
19.218	2-Undecanol	C_11_H_24_O	113,666–64–1	8.67
20.849	2-Tetradecanone	C_14_H_28_O	2,345–27–9	13.26
23.703	Hexadecanoic acid, methyl ester	C_17_H_34_O_2_	112–39–0	11.16
23.933	9-Hexadecenoic acid, methyl ester, (Z)-	C_17_H_32_O_2_	1,120–25–8	9.39
25.544	Octadecanoic acid, methyl ester	C_19_H_38_O_2_	112–61–8	10.30
25.780	9-Octadecenoic acid (Z)-, methyl ester	C_19_H_36_O_2_	112–62–9	11.63
26.306	9,12-Octadecadienoic acid (Z,Z)-, methyl ester	C_19_H_34_O_2_	112–63–0	11.14
39.385	Geranylgeraniol	C_20_H_34_O	24,034–73–9	10.34

### 2.10 RNA extraction and qRT-PCR analysis

Total RNA was extracted from the mycelia using a spin column fungal total RNA purification Kit (Sangon Biotech (Shanghai) Co., Ltd). First-strand cDNA was synthesized using a ToloScript All-in-one RT EasyMix (TOLO Biotech (Shanghai) Co., Ltd). The resulting cDNA was used as the template for subsequent polymerase chain reaction (PCR). Quantitative real-time PCR (qRT-PCR) was performed using a Q3 SYBR qPCR Master mix (TOLOBIO) on a QuantStudio™ Real-Time PCR Detection System. The relative expression levels were calculated using the 2^(−ΔΔCt) method. Each treatment included three biological replicates, and the entire experiment was independently repeated twice. The actin gene *Actin* was used as the internal reference for normalization. Primers for these genes [*Actin* and *CCS2* (Zhang and Sun, 2018), *CHI* (Huang et al., 2019), *CAT* and *SOD* (in this study)] are listed in Supplementary Table 1. The primers for *CAT* and *SOD* were designed with Primer 3 Plus version 3.3.0 software and subsequently validated. The *CAT* gene primer sequences were designed based on the *M. oryzae* catalase gene (GenBank no. NC_017851.1), while the *SOD* gene primers were based on the *M. oryzae* superoxide dismutase gene (GenBank no. NC_017849.1).

### 2.11 Statistical analysis

All quantitative data were analyzed using GraphPad Prism 9 software. Statistical comparisons between the treatment group and the control were performed using unpaired Student’s *t*-tests. For comparisons involving multiple groups, one-way analysis of variance (ANOVA) was conducted, followed by Dunnett’s *post hoc* test for comparisons against a single control group. Differences were considered statistically significant at *p* < 0.05. Data are presented as the mean ± standard deviation (SD).

## 3 Results

### 3.1 Inhibitory effects of GB519 VOCs on mycelial growth and morphology of *M. oryzae*

**FIGURE 1:**
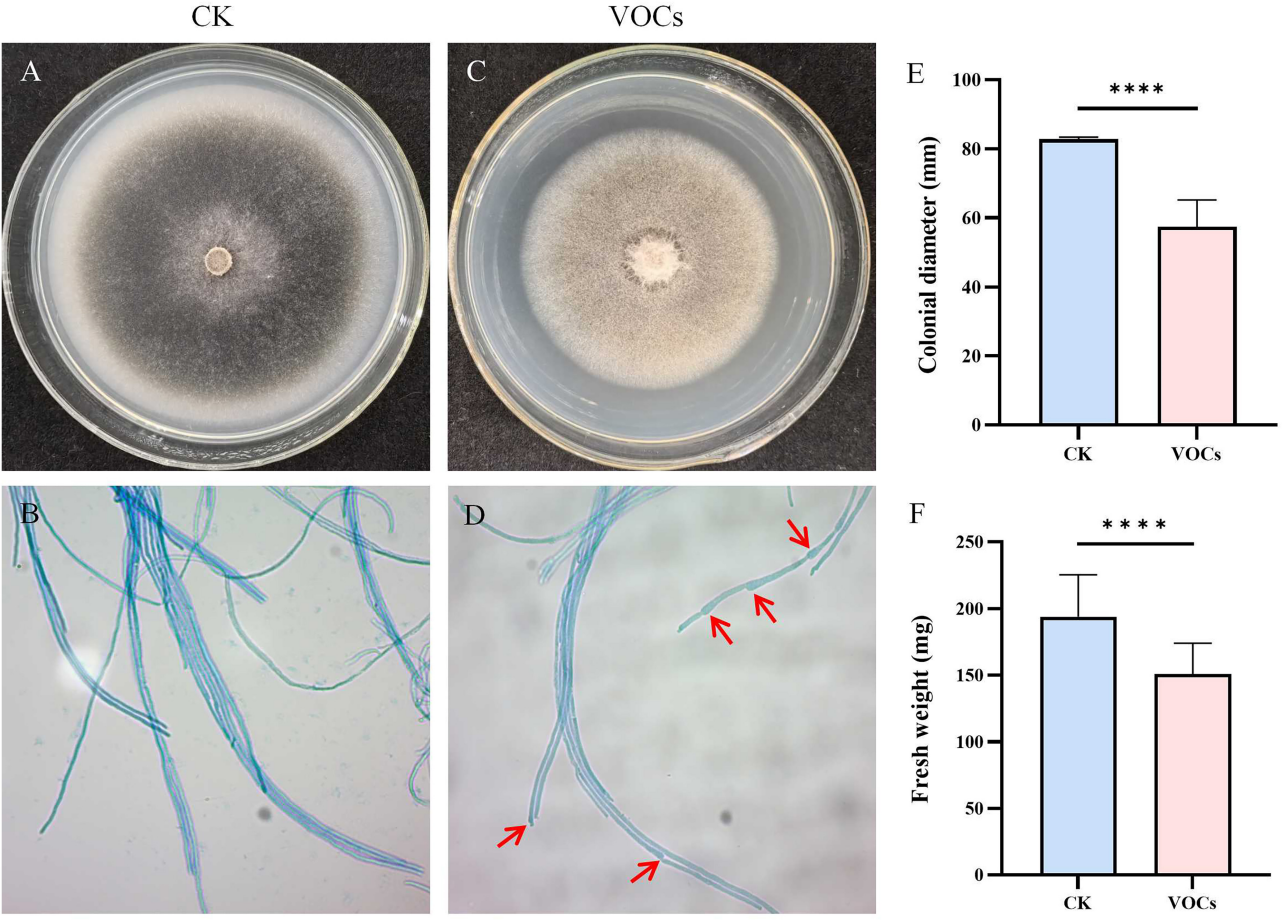
Effect of antifungal activity of VOCs released by GB519 against *Magnaporthe oryzae*. **(A)** Colonial morphologies of *M. oryzae* in the control. **(B)** Colonial morphologies of *M. oryzae* in the presence of VOCs. **(C)** Microscopic observations of *M. oryzae* mycelia in the control. **(D)** Microscopic observations of *M. oryzae* mycelia in the presence of VOCs. **(E)** Colonial diameter of mycelial growth in the presence of VOCs. **(F)** Fresh weight of mycelial growth in the presence of VOCs. Red arrows indicate mycelia abnormality treated by VOCs. All error bars represent standard deviations (*n* = 30). Asterisk indicates significant difference compared with the control (*p* < 0.05). Significance codes for *p*-values: ****p < 0.0001.

**FIGURE 2:**
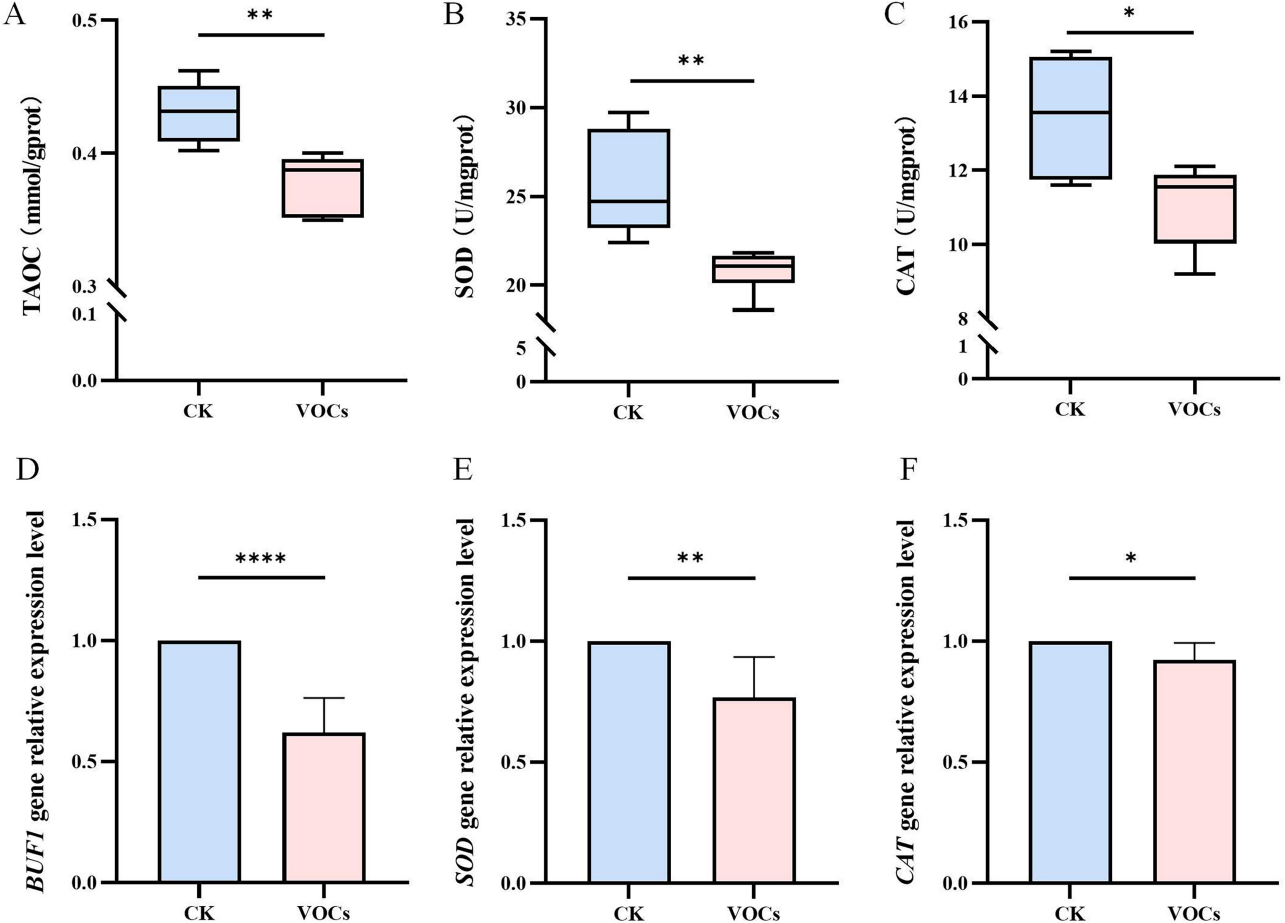
Effect of VOCs released by GB519 on **(A)** the total antioxidant capacity (TAOC), **(B)** superoxide dismutase activity (SOD), **(C)** catalase activity (CAT), **(D)** BUF1 gene expression, **(E)** SOD gene expression, and **(F)** CAT gene expression in *Magnaporthe oryzae* mycelia. All error bars represent standard deviations (*n* = 6). Asterisk indicates significant difference compared with the control (*p* < 0.05). Significance codes for *p*-values: ****p < 0.0001.

Using the double petri dish method, we assessed the effects of GB519 VOCs on *M. oryzae*. In the control group, *M. oryzae* colonies appeared black with normal pigmentation and uniform mycelial structure (Figures 1A,B). In contrast, the colonies treated with GB519 VOCs showed a diminished size and reduced pigmentation, indicating an interference with fungal growth and pigment synthesis (Figures 1C,D). Quantitative PCR analysis demonstrated a significant downregulation in the expression of *BUF1*, a key melanin biosynthesis gene, which decreased to 0.62-fold relative to the control (*p* < 0.05) (Figure 2D).

Furthermore, mycelial growth of *M. oryzae* was significantly inhibited. In the control, the mycelia exhibited a uniform color, smooth surface, and consistent thickness (Figure 1B). In contrast, the treated mycelia exhibited uneven pigmentation and size, with tips significantly thinner than the other parts, resulting in irregular mycelial thickness (Figure 1D).

Additionally, GB519 VOCs demonstrated significant activity against *M. oryzae*. Compared to the control, the colony diameter on treated plates was reduced to 57.4 mm, corresponding to an inhibition rate of 30.7% (Figure 1E). Concurrently, the mycelial fresh weight decreased to 150.9 mg, representing a 21.5% reduction (Figure 1F). These results confirm that GB519 VOCs impair mycelial growth and alter morphology of *M. oryzae*, but did not completely suppress fungal growth.

### 3.2 Accumulation of ROS in *M. oryzae* mycelia treated with GB519 VOCs

**FIGURE 3:**
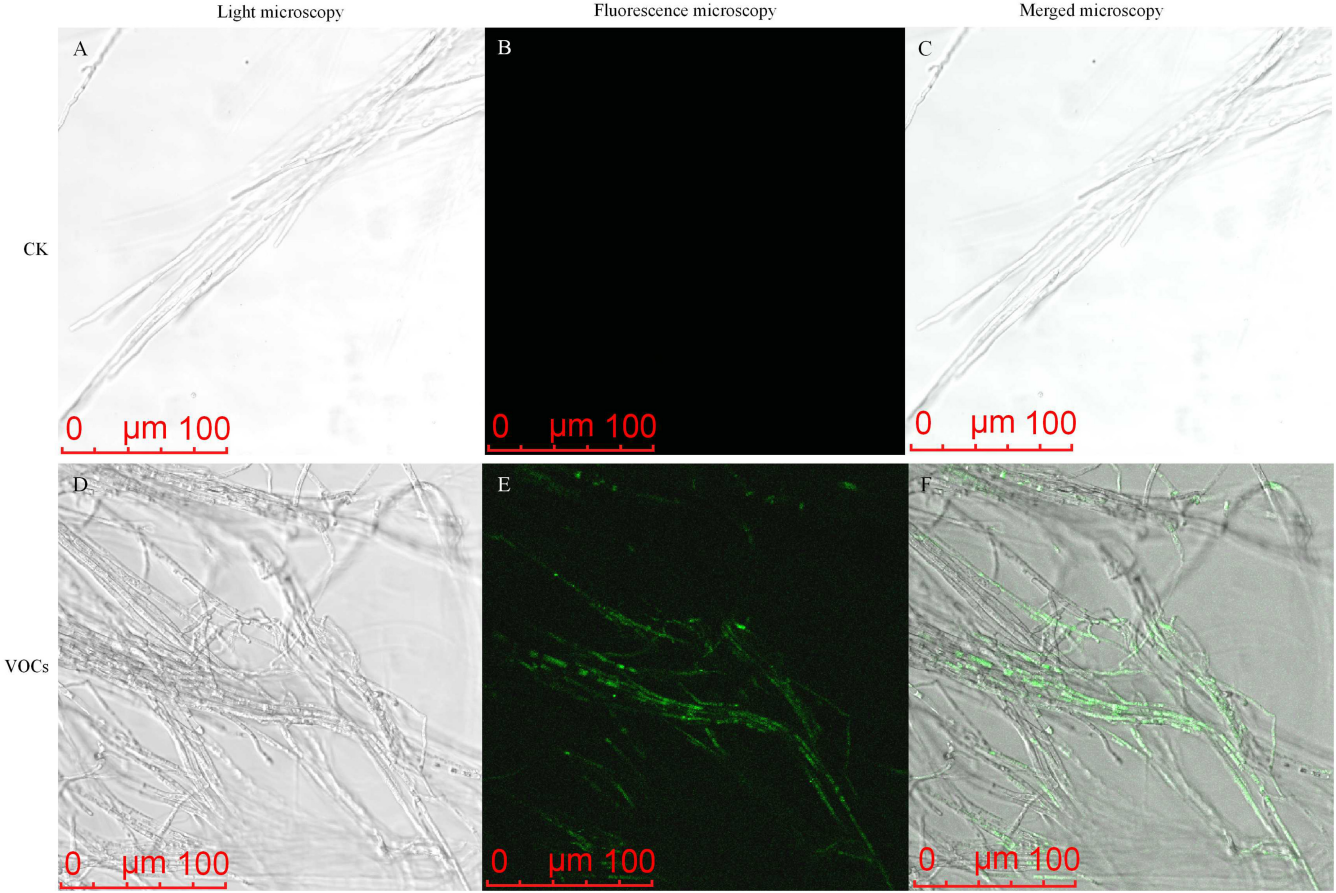
Reactive oxygen species (ROS) accumulation in *Magnaporthe oryzae* mycelia after the treatment with VOCs released by GB519. ROS accumulation was visualized by light microscopy **(A,D)**, fluorescence microscopy **(B,E)**, and merged microscopy **(C,F)**.

To investigate the effects of GB519 VOCs on cellular oxidative stress, reactive oxygen species (ROS) in *M. oryzae* were assessed using fluorescent dye DCFH-DA (Lee et al., 2020). The treated mycelia displayed more intense green fluorescence compared to the control, implying a significant accumulation of ROS (Figure 3E).

Correspondingly, the activities of antioxidative enzymes were significantly reduced in the treated mycelia from those in the control. Total antioxidant capacity (TAOC) decreased to 0.379 mmoL/g protein in the treatment, representing an 11.9% reduction (Figure 2A). Catalase (CAT) and superoxide dismutase (SOD) activities were 11.1 U/mg and 21.1 U/mg protein, with reduction rates of 17.2 and 17.7%, respectively (Figures 2B,C).

qRT-PCR analysis confirmed these findings at the transcription level. The relative expressions of *SOD* and *CAT* genes in the treated mycelia were reduced to 0.77-fold and 0.92-fold from those in the control (Figures 2E,F). The findings suggest that GB519 VOCs suppressed the expressions of genes essential for antioxidative enzymes, thereby markedly reducing enzyme activity and, consequently, compromising both mycelial growth and morphology.

**FIGURE 4:**
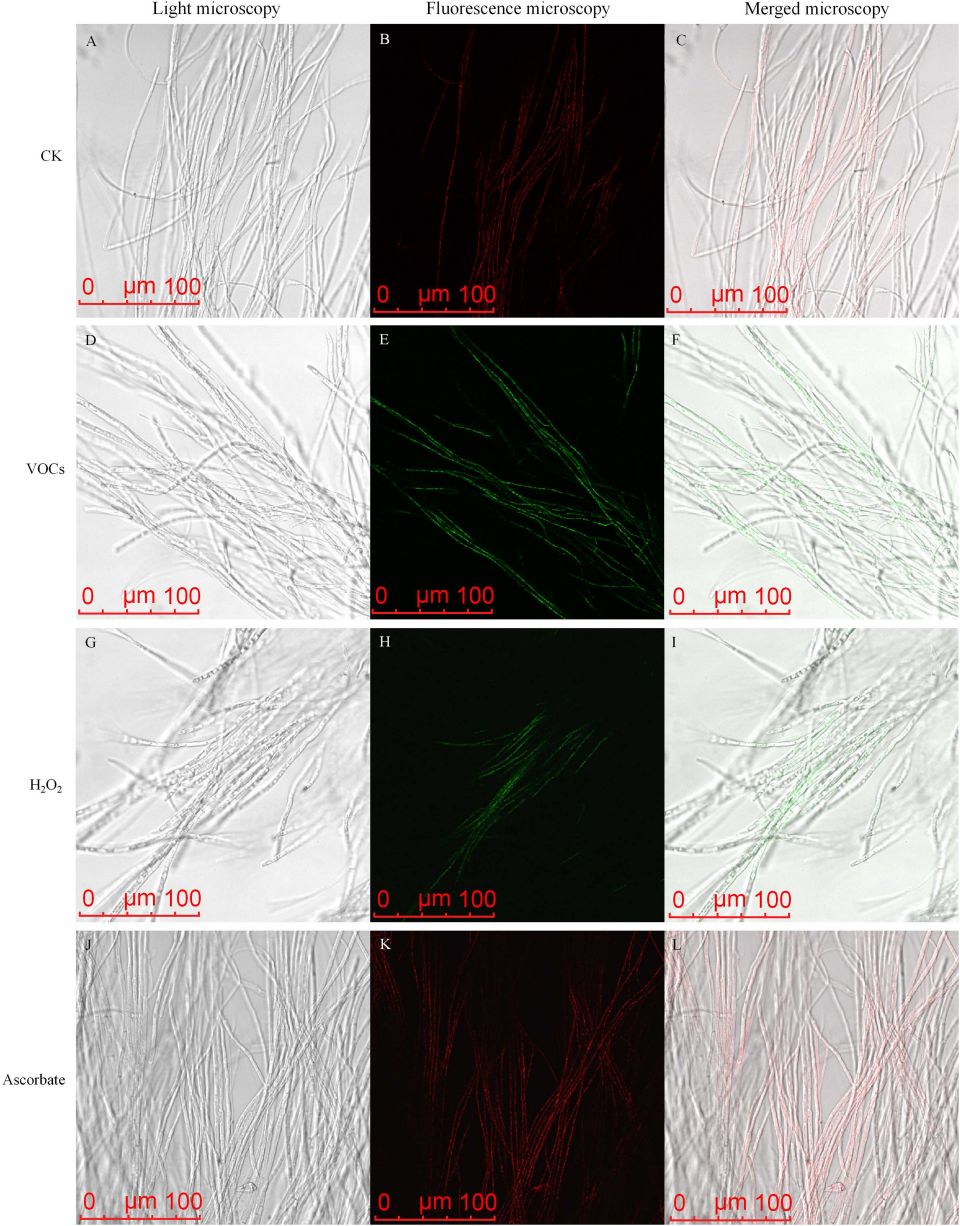
Effect of VOCs released by GB519 on mitochondrial membrane potential (MMP) in *Magnaporthe oryzae* mycelia. MMP was observed by light microscopy **(A,D,G,J)**, fluorescence microscopy **(B,E,H,K)** and merged microscopy **(C,F,I,L)**.

ROS accumulation affects mitochondrial membrane potential (MMP) activity (Kuang et al., 2017). JC-1 staining showed a reduction of MMP from GB519 VOCs. The mycelia of *M. oryzae* in the control group and those treated with ascorbate exhibited prominent red fluorescence, indicating intact mitochondrial function (Figures 4A–C,J–L). In contrast, the mycelia treated with GB519 VOCs and H_2_O_2_ displayed green fluorescence, indicating compromised mitochondrial function (Figures 4D–F,G–I). Together, these results demonstrated that GB519 VOCs induce oxidative stress by impairing both antioxidant defenses and mitochondrial integrity.

### 3.3 Effect of GB519 VOCs on cell wall and membrane integrity

**FIGURE 5:**
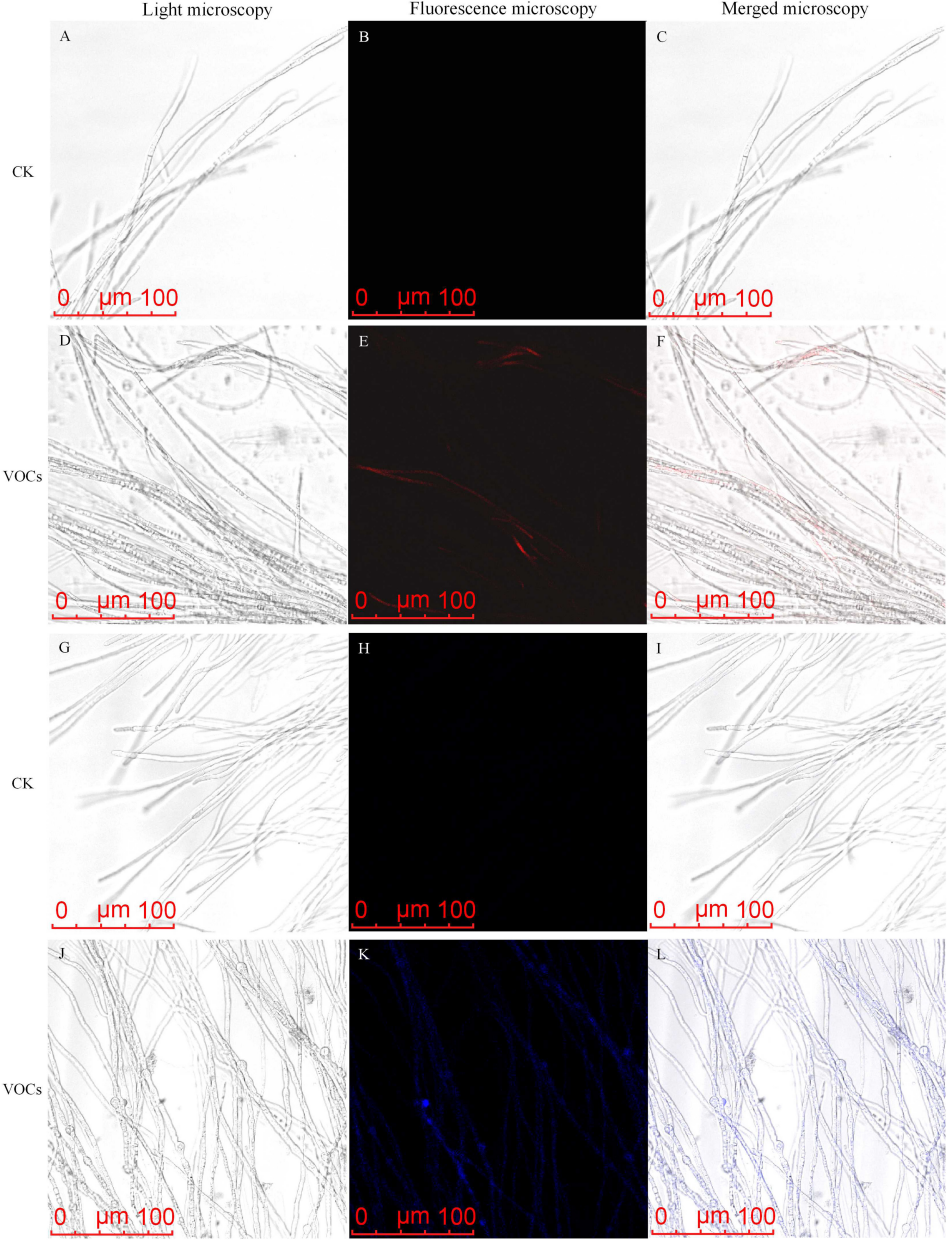
Propidium iodide (PI) staining **(A–F)** and Hoechst 33258 staining **(G–L)** in *Magnaporthe oryzae* mycelia after the treatment with VOCs released by GB519, visualized by light microscopy **(A,D,G,J)** and fluorescence microscopy **(B,E,H,K)** and merged microscopy **(C,F,I,L)**.

Propidium iodide (PI) staining resulted in a stronger red fluorescence in the mycelia treated with GB519 VOCs than those in the control, indicating membrane damage and loss of integrity (Figure 5E). Hoechst 33258 staining further showed intense blue fluorescence in the treated mycelial, signifying chromatin condensation (Figure 5K), which was absent in the control with faint blue fluorescence (Figure 5H).

**FIGURE 6:**
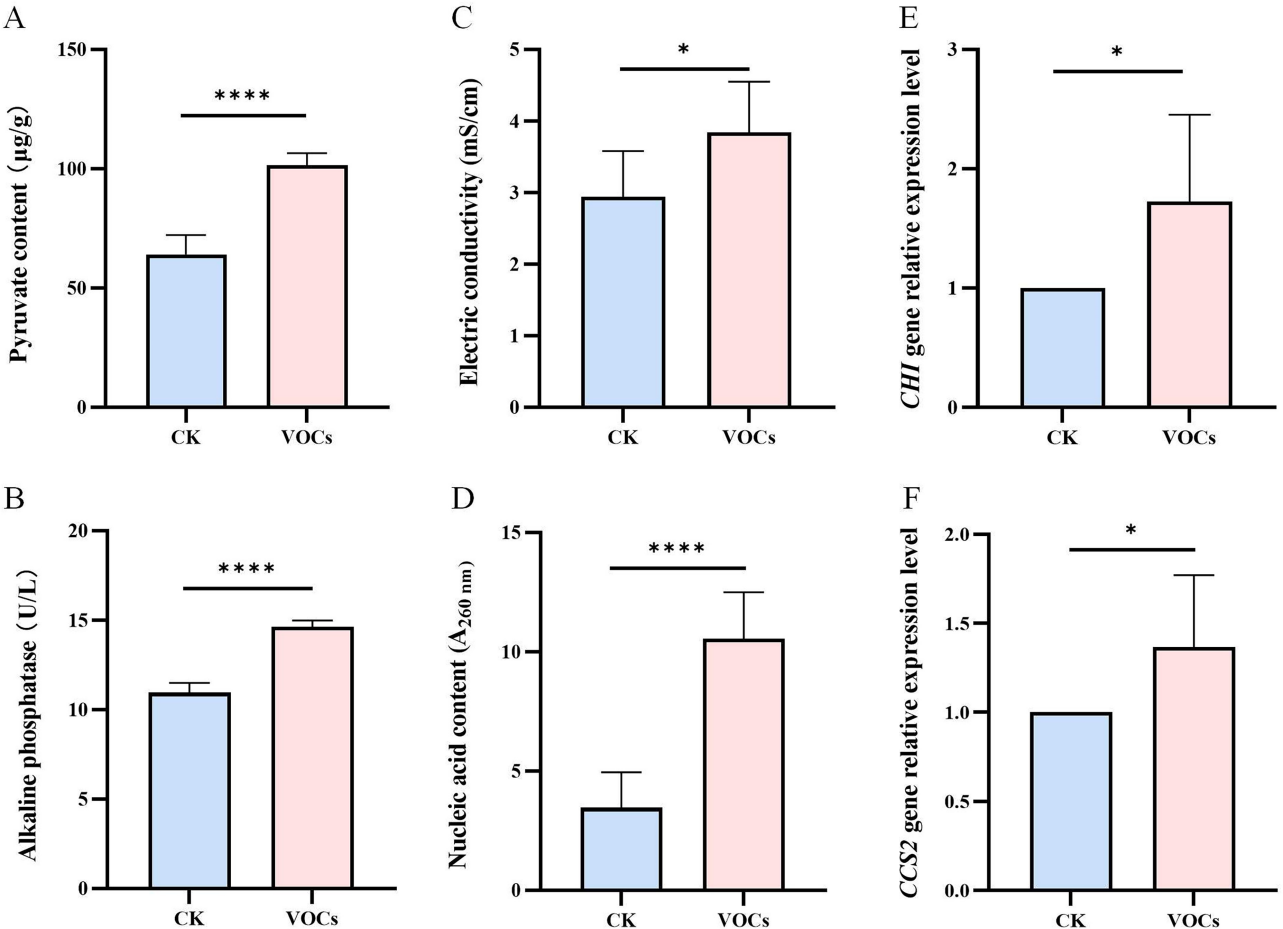
Effect of VOCs released by GB519 on the pyruvate content **(A)**, alkaline phosphatase (AKP) activity **(B)**, electric conductivity **(C)**, nucleic acids content **(D)**, CHI gene expression **(E)**, and CCS2 gene expression **(F)** in *Magnaporthe oryzae* mycelia. All error bars represent standard deviations (*n* = 6). Asterisk indicates significant difference compared with the control (*p* < 0.05). Significance codes for *p*-values: *p < 0.05; ****p < 0.0001.

Metabolic indicators of membrane damage also increased significantly upon exposure to GB519 VOCs. Pyruvate content rose significantly from 64.1 μg/g in the control to 101.4 μg/g in the treatment (Figure 6A), and alkaline phosphatase (AKP) content increased from 10.9 μg/g to 14.6 μg/g in response to the VOCs (Figure 6B). Likewise, electrical conductivity rose from 2.94 to 3.84 mS/cm (Figure 6C), and the absorbance at 260 nm (A_260_), indicative of nucleic acids leakage, increased significantly from 3.5 to 10.6 units (Figure 6D), indicating membrane disruption and intracellular leakage.

qRT-PCR analysis revealed that GB519 VOCs significantly upregulated the expression of the chitinase gene *CHI* (1.72-fold) and the DNA damage response gene *CCS2* (condensin complex subunit 2; 1.37-fold) (Figures 6E,F), indicating stress-induced cell wall remodeling and genotoxic effects.

### 3.4 Antifungal activity of GB519 VOCs against blast disease

**FIGURE 7:**
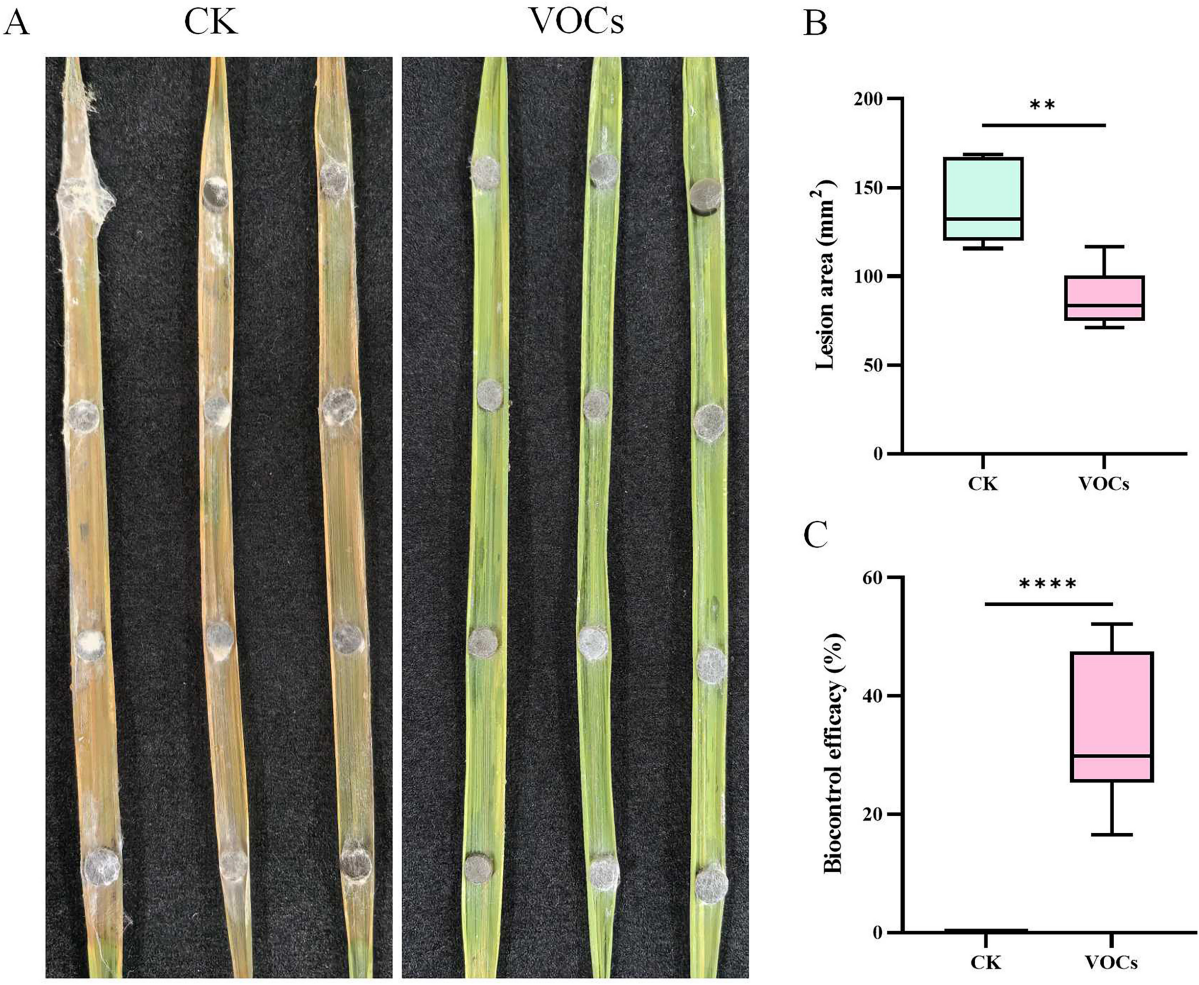
Mycelia growth of *Magnaporthe oryzae* exposed to VOCs from GB519 and control in vivo. **(A)** The rice leaves inoculated with mycelia plugs, **(B)** rot area in leaves, **(C)** biocontrol efficacy in leaves. All error bars represent standard deviations (*n* = 6). Asterisk indicates significant difference compared with the control (*p* < 0.05). Significance codes for *p*-values: **p < 0.01; ****p < 0.0001.

In the detached rice leaf assay, GB519 VOCs significantly suppressed blast disease caused by *M. oryzae* (Figure 7). At 7 days post-inoculation (dpi), the untreated leaves exhibited extensive chlorosis and large lesions (139.6 mm^2^), while the treated leaves remained largely green with small lesions (87.6 mm^2^) (Figures 7A,B). Under these *in vitro* conditions, the GB519 VOCs treatment achieved a disease control efficacy of 33.8% (*p* < 0.05, Figure 7C), indicating its potential to reduce disease severity.

### 3.5 Broad-spectrum antifungal activities of GB519 VOCs

GB519 VOCs displayed inhibitory effects on the mycelial growth of several phytopathogens, including *F. fujikuroi*, *F. oxysporum*, *F. graminearum*, *R. solani*, and *S. rolfsii* (Supplementary Figure 1).

The most pronounced inhibition was observed in *F. fujikuroi*, where GB519 VOCs significantly reduced the colony diameter to 46.7 mm (a 43.7% reduction) and the fresh weight to 147.8 mg (a 24.8% reduction), relative to the control. Similarly, *S. rolfsii* exhibited a high sensitivity, demonstrated by a 42.3% decrease in colony diameter and a 28.7% reduction in fresh weight. The inhibition to *F. graminearum* and *F. oxysporum* was displayed by 37.4 and 35.6% reduction in diameter, and 22.8 and 25.7% reduction in fresh weight, respectively. *R. solani* was the least affected, exhibiting reductions in mycelial growth and fresh weight of 32.7 and 25.5%, respectively. These results suggest that GB519 VOCs possess broad-spectrum antifungal potential and could be used to control several other diseases.

### 3.6 Identification and verification of antifungal VOCs from *B. subtilis* GB519

SPME-GC-MS analysis initially detected 51 distinct peaks from *B. subtilis* GB519 cultures. By applying stringent background subtraction criteria-including comparison with sterile-medium controls and blank runs, setting thresholds for peak area ( > 20-fold larger than control) and relative abundance ( > 0.1%), and requiring a high-confidence NIST library match (Match Factor > 80%)-we confirmed 12 of these compounds as unique GB519-derived VOCs (Table 1). The overall profile of VOCs produced by *B. subtilis* GB519 is shown in the total ion chromatogram (Supplementary Chromatogram 1), while the background from the LB medium is presented in Supplementary Chromatogram 2. These compounds included: 3 ketones: 2-Butanone, 3-hydroxy-; 2-Nonanone; and 2-Tetradecanone. 2 heterocycles: Pyrazine, 2, 6-dimethyl-; and Pyrazine, 3-ethyl-2, 5-dimethyl-. 5 esters: Hexadecanoic acid, methyl ester; 9-Hexadecenoic acid, methyl ester, (Z)-; Octadecanoic acid, methyl ester; 9-Octadecenoic acid (Z)-, methyl ester; 9, 12-Octadecadienoic acid (Z, Z)-, methyl ester. 1 alcohol: 2-Undecanol, and 1 terpene: Geranylgeraniol.

### 3.7 Antifungal activity test of pure volatile compound

We have acquired all 12 volatile organic compounds (VOCs) to be used as analytical standards in subsequent experiments. These compounds represent potential bioactive constituents derived from GB519 that are likely responsible for the antifungal activities observed in this study (Supplementary Figure 2). Among them, 2-tetradecanone exhibited the strongest antifungal effect, with an inhibition zone diameter of 74.7 mm and an inhibition rate of 10.20%.

## 4 Discussion

Although effective, chemical fungicides pose substantial risks, including environmental pollution and potential harm to human health. These concerns have underscored an urgent need for the development of efficient and sustainable biological alternatives (Zhou et al., 2014; Madlhophe et al., 2025). Among such alternatives, *Bacillus* spp., particularly those strains that produce antimicrobial volatile organic compounds (VOCs), show great promise in both agricultural and environmental applications (Koilybayeva et al., 2023). *B. subtilis* has been widely reported to effectively control rice blast disease through the production of various antimicrobial metabolites, such as lytic enzymes, soluble antibiotics, and nonribosomal peptides (Zhu et al., 2020; Zhu F. et al., 2021). These metabolites play crucial roles in inhibiting the growth and virulence of blast fungus, thereby providing a promising biocontrol strategy for controlling rice blast disease. Unlike soluble metabolites that require direct contact, VOCs diffuse through air and soil, allowing them to exert long-distance biocontrol activity and antagonistic effects over broad spatial scales (Santoro et al., 2011). Notably, both VOCs and soluble antifungal compounds contribute comparably to antifungal efficacy, albeit through distinct mechanisms (Xie et al., 2018). While the induction of ROS accumulation, membrane damage, and mitochondrial dysfunction are recognized as common antifungal mechanisms of *Bacillus* VOCs, the precise sequence of events and the relative contribution of specific physiological disruptions can vary among different bacterial strains due to their unique volatilomes. This study provides evidence for a mechanistic link between GB519 VOCs and the disruption of the fungal antioxidant system, with the observed downregulation of antioxidant genes at the transcriptional level being a key associated event.

In this study, GB519 VOCs significantly inhibited the mycelial growth of *M. oryzae*, as demonstrated by large reductions by up to 30.7% in colony diameter and 20.9% in fresh weight (Figure 1). Microscopy revealed a remarkable deformation of mycelial, including irregular thickness and structural deformities of cell walls. These findings align with many other studies, demonstrating that the VOCs from various microbial species alter fungal morphology and deform cell structure, including *Pseudomonas fluorescens* ALB 7B (Zhou et al., 2014), *B. amyloliquefaciens* L3 (Wu et al., 2019), and *Sarocladium brachiariae* HND5 (Yang et al., 2021). Morphological changes in the pathogenic fungi following exposure to GB519 VOCs suggest potential impacts on cellular integrity. In *M. oryzae*, a visible reduction in mycelial pigmentation was observed, which is commonly associated with melanin production-a known virulence factor. Concurrently, the expression of the melanin biosynthesis gene *BUF1* exhibited a decreasing trend (downregulated by 38%). Although this level of downregulation may fall below the conventional threshold for definitive biological significance, the combined observation of phenotypic change and gene expression trend is consistent with earlier reports on the antifungal mechanisms of microbial VOCs (Zhu S. Y. et al., 2021; Rudhra et al., 2024). Thus, our data suggest that GB519 VOCs may interfere with melanin-related pathways, warranting further investigation to confirm this effect and quantify melanin content directly.

Furthermore, the GB519 VOCs exhibited broad-spectrum activity against many other phytopathogens, where significant inhibitions on both mycelial growth and biomass accumulation were observed in five fungal species (Supplementary Figure 1), consistent with the findings from *Pseudomonas* protegens strain CHA0 on Basidiomycete species (Prigigallo et al., 2021).

Our *in vitro* detached leaf assay demonstrated the protective efficacy of GB519 VOCs against *M. oryzae*, as displayed by significantly smaller lesion areas on the treated leaves, which were green since they retained chlorophyll. It should be noted that this assay, while indicative, cannot fully replicate the complex interactions in field conditions. Therefore, the efficacy data primarily illustrate the direct *in vitro* bioactivity of the VOCs. Similar biocontrol potential has been observed with other *Bacillus* strains in different pathosystems, such as *B. pumilus* B19 against gray mold in apples, and *Bacillus* TB09/TB72 against anthracnose in mangoes (Jamalizadeh et al., 2010; Zheng et al., 2013).

Many studies have demonstrated that the cell membrane is an important target of antimicrobial agents (Zhornik et al., 2015; Mari et al., 2016; Zhao et al., 2019). Damages to membrane integrity result in permeability of the cell, leading to the leakage of intracellular macromolecules (e.g., proteins, phosphates, DNA, and RNA) into the extracellular environment (Vitro et al., 2005). In this study, propidium iodide and Hoechst 33,258 staining showed an increase of red and blue fluorescence in the treated *M. oryzae* mycelia treated with GB519 VOCs (Figure 5), consistent with reported results (Ye et al., 2020; Zhang et al., 2021).

Our staining results were corroborated by elevated concentrations of extracellular pyruvate (59.9%) and nucleic acids (526.3%), and an increase in solution electrical conductivity (31.8%) (Figures 6A,C,D), all indicative of cytoplasmic leakage due to compromised membrane integrity.

Consistent with the staining, membrane integrity was quantitatively assessed. GB519 VOCs triggered the leakage of intracellular components, evidenced by increases in extracellular pyruvate, electrical conductivity, and in the absorbance at 260 nm (Figures 6A,C,D). The A260 value, while not a direct measure of absolute cell death, serves as a sensitive indicator of nucleic acids leakage and, thereby, the severity of membrane compromise. Similarly, the VOCs emitted by *Rahnella aquatilis* JZ-GX1 compromise the membrane integrity of *C. gloeosporioides*, as evidenced by a significant elevation of absorbance at 260 nm (Kong et al., 2020). A significantly higher A_260_ nm and electrical conductivity have been found after tea tree oil treatment (Shao et al., 2013).

Alkaline phosphatase (AKP) is synthesized in the cytoplasm, secreted into the periplasmic space, and leaks from damaged cell into the wall layers (Cheng et al., 1970; Lindsay et al., 1973). In our study, AKP activity was significantly increased by 33.7% upon GB519 VOCs exposure, suggesting early damage to the cell wall of *M. oryzae*. Similarly, a higher AKP activity in *B. cinerea* has been observed after tea tree oil treatment (Shao et al., 2013). Additionally, we observed an upregulation of *CHI* and *CCS2* genes associated with chitinase synthesis and DNA repair in *M. oryzae* after treatment with GB519 VOCs, implying a compensatory response to cellular stress and damage (Figures 6E,F). These findings align with the observations from Huang et al. (2019) for the *CHI* gene and (Zhang and Sun, 2018) for the *CCS2* gene.

An accumulation of reactive oxygen species (ROS) is a well-documented mechanism of microbial-induced cell death *via* apoptosis, necroptosis, or ferroptosis (Cadenas and Davies, 2000). Consistent with this mechanism, An increase in ROS accumulation was observed in *M. oryzae* mycelia following treatment with GB519 VOCs (Figure 3). Other studies have demonstrated that VOCs from *Corallococcus* sp. EGB, *S. brachiariae* HND5, and *P. chlororaphis* subsp. *aureofaciens* SPS-41 induce fungal cell death by triggering ROS accumulation-mediated oxidative stress (Ye et al., 2020; Yang et al., 2021; Zhang et al., 2021).

Beyond confirming ROS accumulation, a key novel finding of this work is the concurrent and coordinated downregulation of the core antioxidant genes *SOD* and *CAT* at the transcriptional level (Figures 2E,F), which was coupled with reduced enzyme activities (Figures 2A–C). Our results indicate that GB519 VOCs not only induce oxidative stress but also impair the pathogen’s primary transcriptional response to neutralize ROS, thereby exacerbating and sustaining the oxidative damage. This two-pronged attack on the redox system represents a more detailed and debilitating mode of action than the general increase in ROS commonly reported.

Although the magnitude of enzymatic inhibition may appear subtle, its biological significance can be interpreted in the context of oxidative stress homeostasis. The antioxidant system operates as a tightly balanced network; even a partial compromise in the activity of its core components (SOD and CAT) could impair the fungus’s ability to detoxify ROS efficiently. This may lead to a progressive accumulation of ROS, moving the cellular redox balance toward a pro-oxidant state.

The observed dissipation of mitochondrial membrane potential (MMP) (Figure 4), further supports the occurrence of mitochondrial dysfunction, a common consequence of unresolved oxidative stress. Thus, our data delineate a coherent pathological sequence for GB519 VOCs: the transcriptional suppression of antioxidant defenses likely predisposes the fungus to severe redox imbalance, which subsequently drives mitochondrial failure. Thus, the coordinated downregulation of antioxidant enzyme genes and activities, even at these levels, likely contributes to an attenuated oxidative stress response in *M. oryzae*. This attenuation, synergizing with direct ROS induction by VOCs and MMP collapse, could collectively drive oxidative damage, ultimately inhibiting fungal growth (Arita et al., 2020; Yang et al., 2020; Zhang et al., 2021).

GC-MS profiling identified 12 VOCs emitted from GB519, including ketones, pyrazines, esters, alcohols, and terpenes, which exhibited varying degrees of antimicrobial activity (Table 1). These compounds showed varying levels of antimicrobial activity. It should be noted that Kovats retention indices were not determined in this study; future work will include n-alkane calibration to strengthen compound identification. For example, 2-nonanone displayed only 5.0% and 2-tetradecanone exhibited only 11.3% antifungal activity against *M. oryzae* (Supplementary Figure 2), consistent with previous studies, suggesting that the synergistic effects of the VOC mixture often outperform individual compounds (Raza et al., 2015; Raza et al., 2016; Wu et al., 2019). This suggestion highlights the complexity of VOC-mediated biocontrol and underscores the importance of identifying strain-specific VOC profiles for effective pathogen targeting (Zhou et al., 2014).

## 5 Conclusion

VOCs produced by *B. subtilis* GB519 exhibit significant antifungal activity against *M. oryzae*, the causal agent of rice blast. The antifungal activity is mediated through multiple mechanisms, including (1) disruption of fungal cell membrane integrity, (2) alteration of mycelial morphology and structure, (3) accumulation of intracellular ROS, (4) reduction of MMP, and (5) increasing cytoplasmic leakage of intracellular compounds, including nucleic acids and pyruvate.

This study demonstrates a promising potential to use the VOCs emitted from *B. subtilis* GB519 as sustainable biocontrol agents for effectively managing rice blast. However, the underlying molecular pathways and synergistic interactions among the VOCs remain to be fully elucidated. Future studies should focus on (1) dissecting the VOC biosynthetic pathway, (2) identifying molecular targets in pathogens, and (3) developing formulation and delivery systems for field applications.

By advancing our understanding of microbial VOCs, this work contributes to the development of environmentally safe, effective, and sustainable strategies for crop production.

## Data Availability

The raw data supporting the conclusions of this article will be made available by the authors, without undue reservation.

## Supplementary material

Supplementary Figure 1 Effect of VOCs released by GB519 against several plant pathogens. **(A)** Colonial morphologies of plant pathogens in the presence of VOCs. **(B)** Colonial Diameter of mycelial growth in the presence of VOCs. **(C)** Fresh weight of mycelial growth in the presence of VOCs. Asterisks indicate a significant difference from the control with *p* < 0.05. All error bars represent standard deviations (*n* = 10). Asterisk indicates significant difference compared with the control (*p* < 0.05). Significance codes for *p*-values: ****, *p* < 0.0001.

Supplementary Figure 2 Effect of antifungal activity of pure volatile compound released by GB519 against *Magnaporthe oryzae*. All error bars represent standard deviations (*n* = 10). Asterisk indicates significant difference compared with the control (p < 0.05). Significance codes for p-values: *, *p* < 0.05; **, *p* < 0.01; ***, *p* < 0.001; ****, *p* < 0.0001; not significant, ns.

Supplementary Chromatogram 1 Chromatographic results of VOCs produced by *B. subtilis* GB519.

Supplementary Chromatogram 2 Chromatographic results of VOCs produced by LB medium.
