# Supplementary material for: Antifungal activity of volatile organic compounds produced by Bacillus subtilis GB519 against blast pathogen Magnaporthe oryzae in rice
Source: Front Microbiol. 2026 Mar 11;17:1757473. doi: 10.3389/fmicb.2026.1757473 (PMC13013540; doi:10.3389/fmicb.2026.1757473)
Supplement: Supplementary file 1 [file Table_1.docx]

| **Gene** | **Primer 5′-3′** | **Primer source** |
| --- | --- | --- |
| *ACTIN* | F: AGCGTGGTATCCTCACTTTGC  R: ATCTGCGTCATCTTCTCTCGG | Zhang and Sun, 2018 |
| *SOD* | F: GGCAGCCTCGACCAATTCAAG  R: CCAGCCGCCGACTTCTCC | This study |
| *CAT* | F: GCAACATCACCTCGGCATCG  R: GAGCCAGCCACGGTAGAGAAG | This study |
| *CHI* | F: TTGAGCCGCATCTCGACTTT  R: CACTCCGTCCATAGAAGCCC | Huang et al., 2019 |
| *CCS2* | F: TGTGCGTGTCGGCAGAATACT  R: CATGTCACCAAGGGCGATAAC | Zhang and Sun, 2018 |
| *BUF1* | F：TGTCTTTACCATCAACACCCG  R：CTTTGATCCTGAGTAGACGGC | Zhu et al. 2020 |

**Supplementary Table 1. Primers used in this study.**
