# Supplementary figures and images for: Antifungal activity of volatile organic compounds produced by Bacillus subtilis GB519 against blast pathogen Magnaporthe oryzae in rice
Source: Front Microbiol. 2026 Mar 11;17:1757473. doi: 10.3389/fmicb.2026.1757473 (PMC13013540; doi:10.3389/fmicb.2026.1757473)

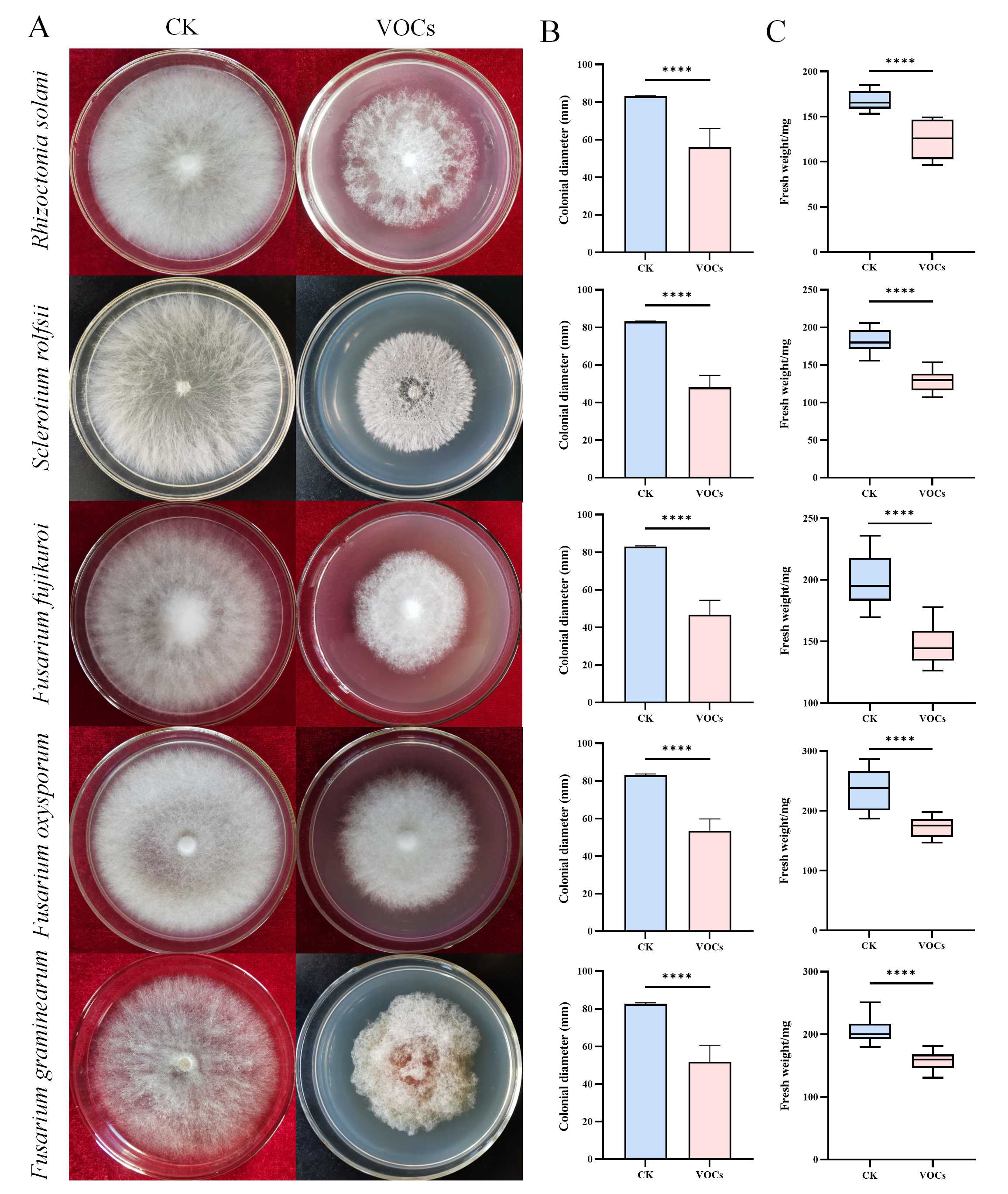

Supplement: Supplementary Figure 1 — Effect of VOCs released by GB519 against several plant pathogens. (A) Colonial morphologies of plant pathogens in the presence of VOCs. (B) Colonial Diameter of mycelial growth in the presence of VOCs. (C) Fresh weight of mycelial growth in the presence of VOCs. Asterisks indicate a significant difference from the control with p < 0.05. All error bars represent standard deviations (n = 10). Asterisk indicates significant difference compared with the control (p < 0.05). Significance codes for p-values: ****, p < 0.0001. [file Image_1.jpg]

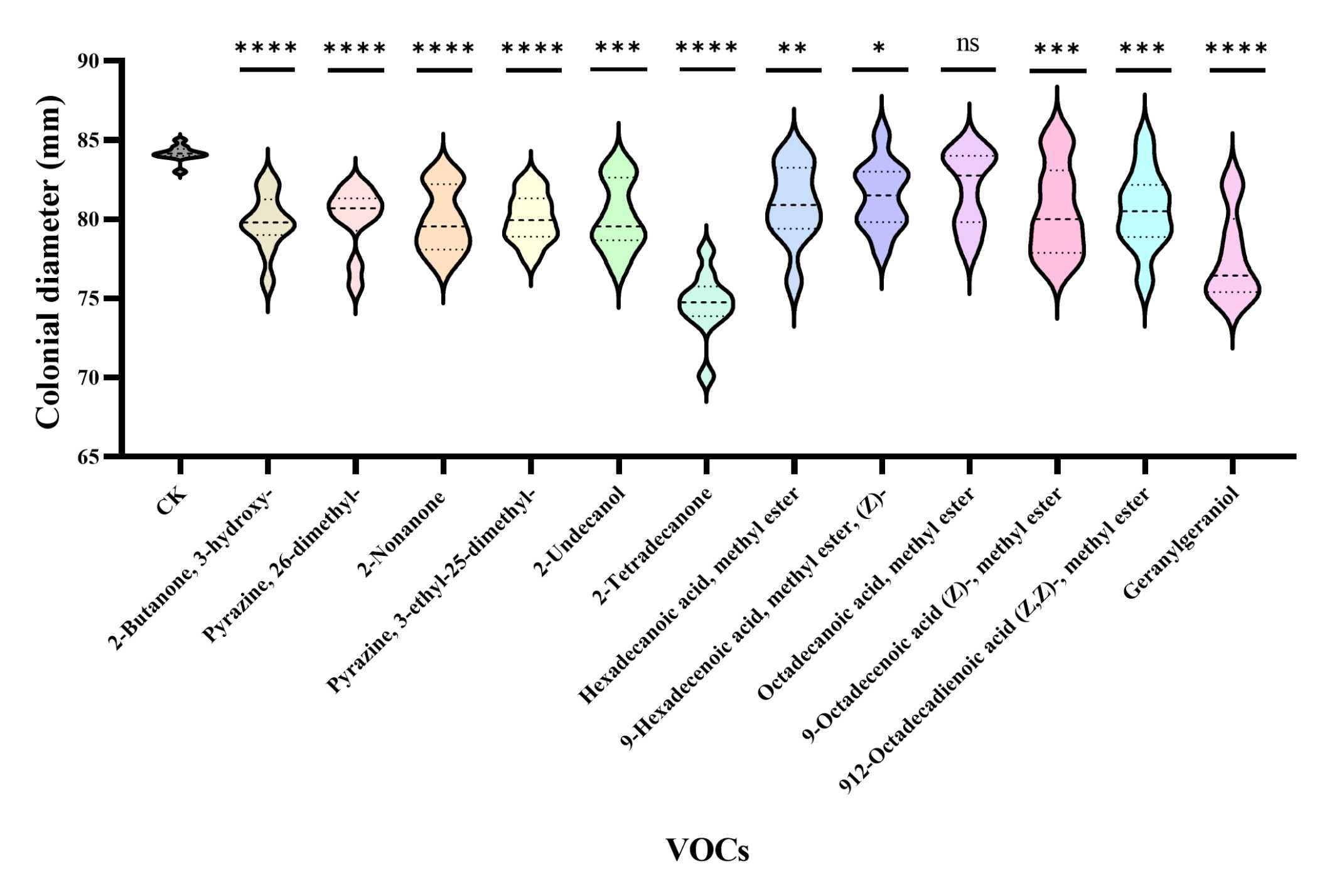

Supplement: Supplementary Figure 2 — Effect of antifungal activity of pure volatile compound released by GB519 against Magnaporthe oryzae. All error bars represent standard deviations (n = 10). Asterisk indicates significant difference compared with the control (p < 0.05). Significance codes for p-values: *, p < 0.05; **, p < 0.01; ***, p < 0.001; ****, p < 0.0001; not significant, ns. [file Image_2.jpg]
