# Supplementary material for: Antifungal activity of volatile organic compounds produced by Bacillus subtilis GB519 against blast pathogen Magnaporthe oryzae in rice
Source: Front Microbiol. 2026 Mar 11;17:1757473. doi: 10.3389/fmicb.2026.1757473 (PMC13013540; doi:10.3389/fmicb.2026.1757473)

未知物分析报告 - 所有匹配

批处理路径	F:\20241203-		
分析文件名称	TEST.uaf		
分析员姓名	5977		
分析时间	2024/12/5 8:02:29		
数据文件名称	Y1-1.D	数据路径名称	F:\20241203-
样品名称	Sample Run on ChemStation	样品类型	样品
采集方法文件	HS.M	采集方法路径	
采集时间	2024/12/3 19:22:04	操作员	MassHunter GC/MS Translator
仪器名称	Instrument #1	稀释	1

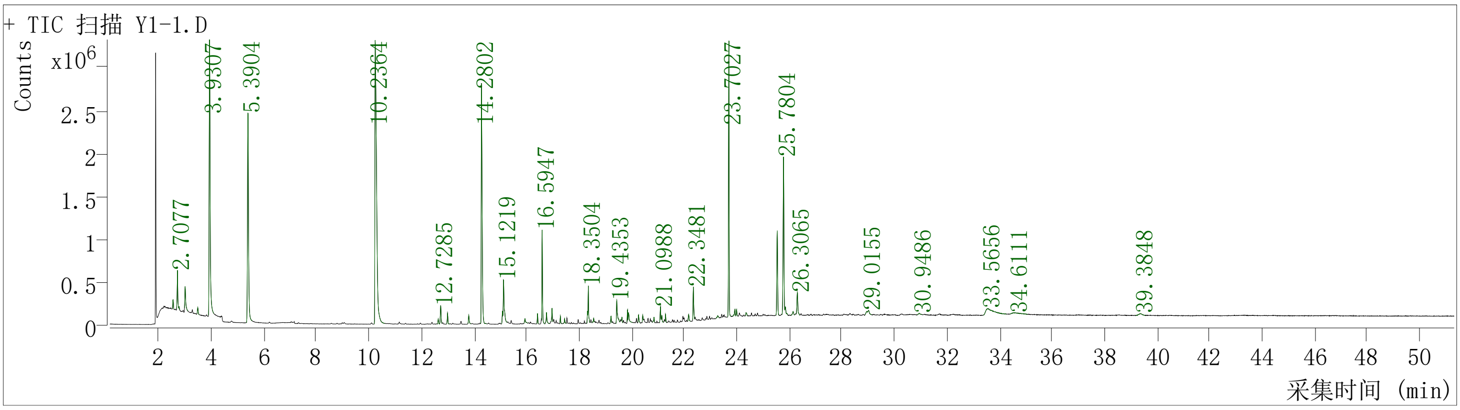

RT	化合物名称	CAS#	分子式	面积	匹配分数	样品	样品
2.5433	Di-tert-butyl peroxide	110-05-4	C8H18O2	190654	95.4	0.29	1.32
2.7077	Acetone	67-64-1	C3H6O	878207	97.6	1.34	6.09
3.0036	Cyclotrisiloxane, hexamethyl-	541-05-9	C6H18O3Si3	680719	98.5	1.04	4.72
3.4836	2-Propanol, 2-methyl-	75-65-0	C4H10O	167790	91.6	0.26	1.16
3.9307	Methylene Chloride	75-09-2	CH2Cl2	8826655	98.1	13.50	61.23
5.3904	Cyclotetrasiloxane, octamethyl-	556-67-2	C8H24O4Si4	6994832	96.4	10.70	48.52
10.2364	Cyclopentasiloxane, decamethyl-	541-02-6	C10H30O5Si5	14415322	98.3	22.06	100.00
12.6364	N,N'-Bis(salicylidene)ethylenediiminocobalt(II)	14167-18-1	C16H14CoN2O2	104549	58.6	0.16	0.73
12.7285	3-Pentanol, 3-(1,1-dimethylethyl)-2,2,4,4-tetramethyl-	41902-42-5	C13H28O	463754	77.7	0.71	3.22
12.9915	2-Butanone, 3-hydroxy-	513-86-0	C4H8O2	230645	94.7	0.35	1.60
13.8002	Pyrazine, 2,6-dimethyl-	108-50-9	C6H8N2	214248	70.6	0.33	1.49
14.2802	Cyclohexasiloxane, dodecamethyl-	540-97-6	C12H36O6Si6	6809135	95.6	10.42	47.24
15.0824	2-Nonanone	821-55-6	C9H18O	222171	90.3	0.34	1.54
15.1219	2,5-Dimethylhexane-2,5-dihydroperoxide	3025-88-5	C8H18O4	1483191	70.6	2.27	10.29
15.9372	Pyrazine, 3-ethyl-2,5-dimethyl-	13360-65-1	C8H12N2	154958	86.6	0.24	1.07
16.4172	2H-Pyranmethanol, tetrahydro-2,5-dimethyl-	54004-46-5	C8H16O2	186013	84.4	0.28	1.29
16.5947	Cycloheptasiloxane, tetradecamethyl-	107-50-6	C14H42O7Si7	1709938	97.5	2.62	11.86
16.7591	Vanillin, tert-butyldimethylsilyl ether	1000352-84-6	C14H22O3Si	296295	78.0	0.45	2.06
16.9695	Benzaldehyde, 2,5-bis[(trimethylsilyl)oxy]-	56114-69-3	C13H22O3Si2	320813	75.6	0.49	2.23
17.2852	2,5-Dimethylhexane-2,5-dihydroperoxide	3025-88-5	C8H18O4	128236	76.5	0.20	0.89
17.4495	Silane, dimethyl(dimethyl(dimethyl(2-isopropylphenoxy)silyloxy)silyloxy)(2-isopropylphenoxy)-	1000347-25-6	C24H40O4Si3	105164	62.6	0.16	0.73
18.3504	Cyclooctasiloxane, hexadecamethyl-	556-68-3	C16H48O8Si8	867367	93.0	1.33	6.02
18.5542	Ethanone, 1-phenyl-2-(1-piperidinyl)-	779-52-2	C13H17NO	153488	68.1	0.23	1.06
19.2183	2-Undecanol	1653-30-1	C11H24O	166978	89.0	0.26	1.16
19.4353	Oxime-, methoxy-phenyl-_	1000222-86-6	C8H9NO2	769810	83.3	1.18	5.34
19.6260	Aniline	62-53-3	C6H7N	155906	87.6	0.24	1.08
19.8429	Cyclononasiloxane, octadecamethyl-	556-71-8	C18H54O9Si9	188298	92.5	0.29	1.31
19.8824	Cyclotrisiloxane, hexamethyl-	541-05-9	C6H18O3Si3	309389	88.8	0.47	2.15
20.2638	Tridecanal	10486-19-8	C13H26O	183396	90.3	0.28	1.27
20.4216	Cyclotetrasiloxane, octamethyl-	556-67-2	C8H24O4Si4	156970	93.9	0.24	1.09

Agilent Trusted Answers

RT	化合物名称	CAS#	分子式	面积	匹配分数	样品	样品
20.8490	2-Tetradecanone	2345-27-9	C14H28O	116077	84.0	0.18	0.81
21.0988	Cyclopentasiloxane, decamethyl-	541-02-6	C10H30O5Si5	322322	63.6	0.49	2.24
21.1514	Cyclodecasiloxane, eicosamethyl-	18772-36-6	C20H60O10Si10	158519	75.1	0.24	1.10
21.2895	Benzyl nitrile	140-29-4	C8H7N	134710	94.0	0.21	0.93
22.1706	Oxirane, hexadecyl-	7390-81-0	C18H36O	174284	87.4	0.27	1.21
22.3481	Nonanoic acid, 9-oxo-, methyl ester	1931-63-1	C10H18O3	747948	96.8	1.14	5.19
23.2753	2-Undecenoic acid	4189-02-0	C11H20O2	100163	61.7	0.15	0.69
23.7027	Hexadecanoic acid, methyl ester	112-39-0	C17H34O2	5367558	98.4	8.21	37.24
23.9328	9-Hexadecenoic acid, methyl ester, (Z)-	1120-25-8	C17H32O2	192296	89.9	0.29	1.33
23.9985	Hexadecanoic acid, ethyl ester	628-97-7	C18H36O2	132458	89.7	0.20	0.92
24.3536	Phenol, 2,4-bis(1,1-dimethylethyl)-	96-76-4	C14H22O	101156	85.2	0.15	0.70
25.5437	Octadecanoic acid, methyl ester	112-61-8	C19H38O2	2103302	98.0	3.22	14.59
25.7804	9-Octadecenoic acid (Z)-, methyl ester	112-62-9	C19H36O2	4271181	99.5	6.53	29.63
25.8528	9-Octadecenoic acid (Z)-, methyl ester	112-62-9	C19H36O2	267769	79.6	0.41	1.86
26.1487	Ethyl Oleate	111-62-6	C20H38O2	135663	80.2	0.21	0.94
26.3065	9,12-Octadecadienoic acid (Z,Z)-, methyl ester	112-63-0	C19H34O2	692797	96.5	1.06	4.81
29.0155	1,2-Benzenedicarboxylic acid, butyl 2-methylpropyl ester	17851-53-5	C16H22O4	377065	74.5	0.58	2.62
30.9486	Pentadecanoic acid	1002-84-2	C15H30O2	101920	68.6	0.16	0.71
33.5656	n-Hexadecanoic acid	57-10-3	C16H32O2	1698163	88.1	2.60	11.78
34.6111	cis-9-Hexadecenoic acid	1000333-19-5	C16H30O2	386794	61.6	0.59	2.68
39.3848	Geranylgeraniol	24034-73-9	C20H34O	242983	68.9	0.37	1.69

RT	化合物名称	CAS#	分子式	面积	匹配分数	样品	样品
2.5433	Di-tert-butyl peroxide	110-05-4	C8H18O2	190654	95.4	0.29	1.32

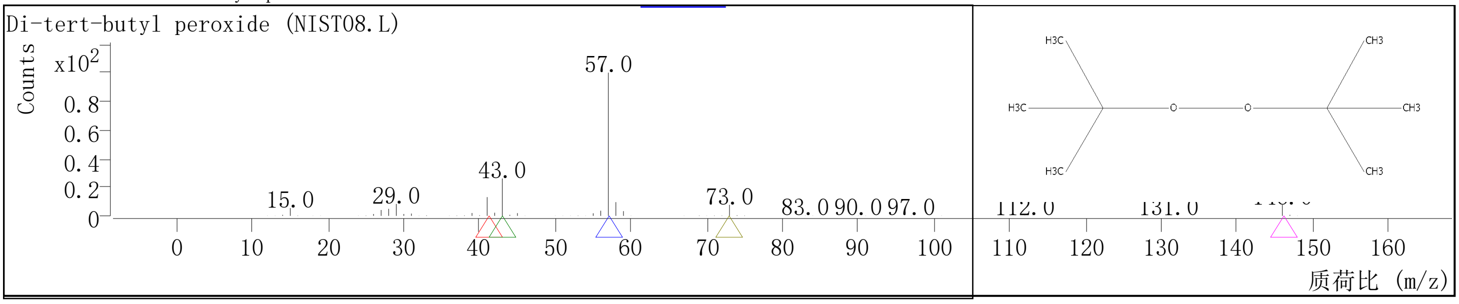

RT	化合物名称	CAS#	分子式	面积	匹配分数	样品	样品
2.7077	Acetone	67-64-1	C3H6O	878207	97.6	1.34	6.09

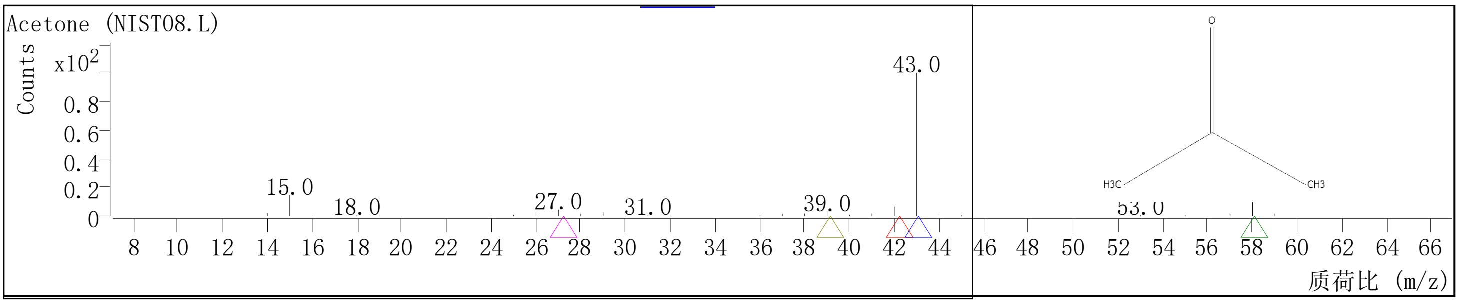

RT	化合物名称	CAS#	分子式	面积	匹配分数	样品	样品
3.0036	Cyclotrisiloxane, hexamethyl-	541-05-9	C6H18O3Si3	680719	98.5	1.04	4.72

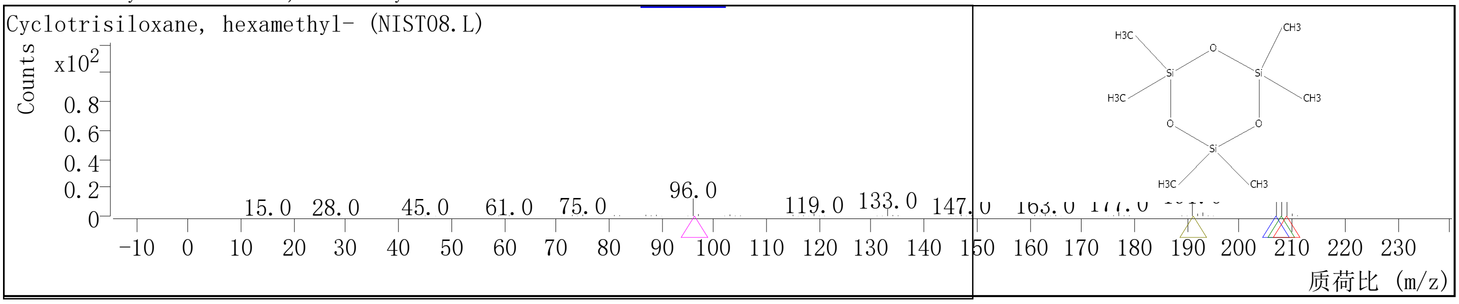

RT	化合物名称	CAS#	分子式	面积	匹配分数	样品	样品
3.4836	2-Propanol, 2-methyl-	75-65-0	C4H10O	167790	91.6	0.26	1.16

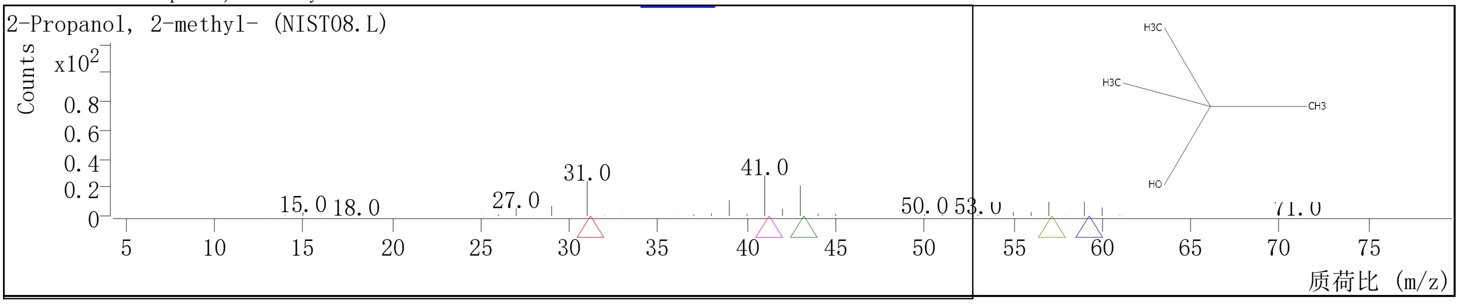

RT	化合物名称	CAS#	分子式	面积	匹配分数	样品	样品
3.9307	Methylene Chloride	75-09-2	CH2Cl2	8826655	98.1	13.50	61.23

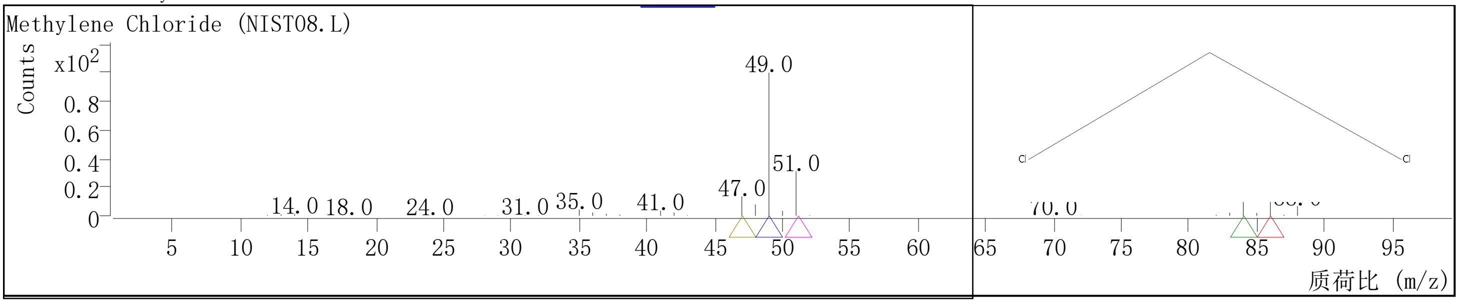

RT	化合物名称	CAS#	分子式	面积	匹配分数	样品	样品
5.3904	Cyclotetrasiloxane, octamethyl-	556-67-2	C8H24O4Si4	6994832	96.4	10.70	48.52

Cyclotetrasiloxane, octamethyl- (NIST08.L)

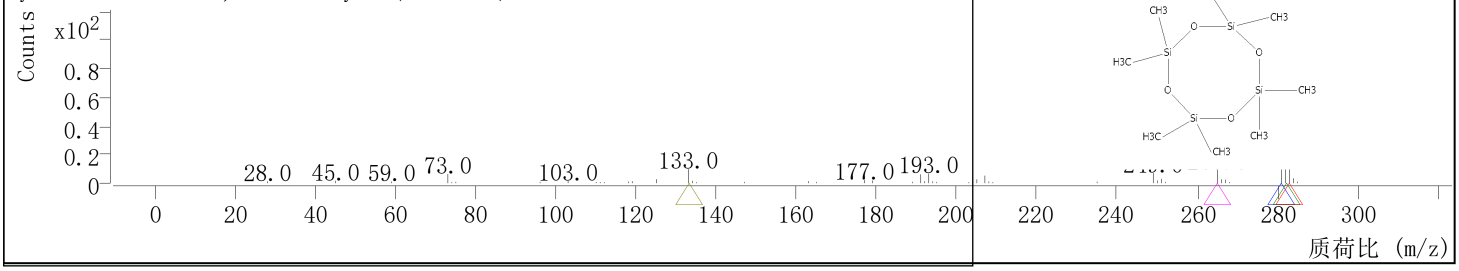

RT	化合物名称	CAS#	分子式	面积	匹配分数	样品	样品
10.2364	Cyclopentasiloxane, decamethyl-	541-02-6	C10H30O5Si5	14415322	98.3	22.06	100.00

Cyclopentasiloxane, decamethyl- (NIST08.L)

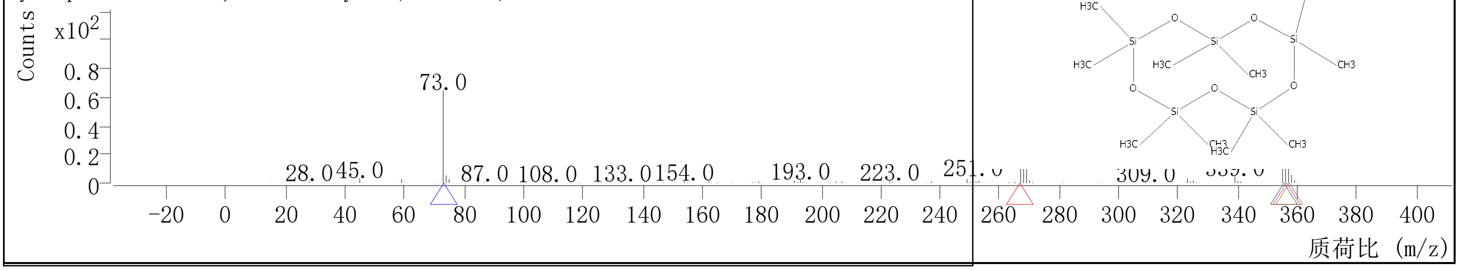

RT	化合物名称	CAS#	分子式	面积	匹配分数	样品	样品
12.6364	N,N'-Bis(salicylidene)ethylenediiminocobalt(II)	14167-18-1	C16H14CoN2O2	104549	58.6	0.16	0.73

N,N'-Bis(salicylidene)ethylenediiminocobalt(II) (NIST08.L)

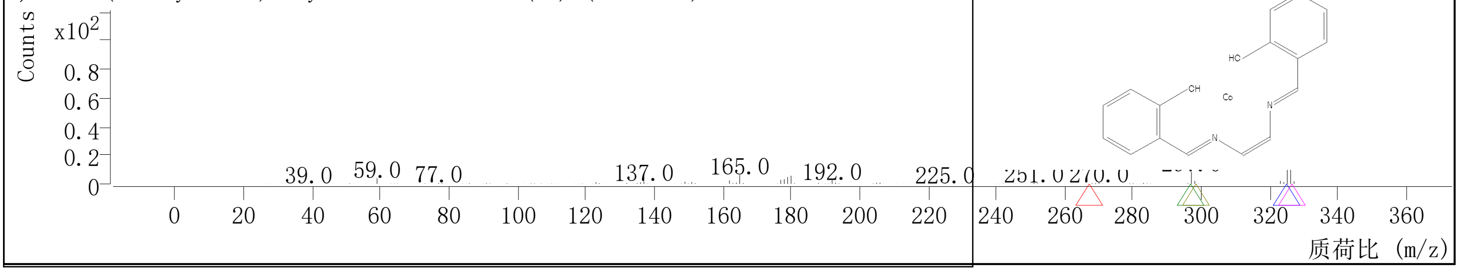

RT	化合物名称	CAS#	分子式	面积	匹配分数	样品	样品
12.7285	3-Pentanol, 3-(1,1-dimethylethyl)-2,2,4,4-tetramethyl-	41902-42-5	C13H28O	463754	77.7	0.71	3.22

3-Pentanol, 3-(1,1-dimethylethyl)-2,2,4,4-tetramethyl- (NIST08.L)

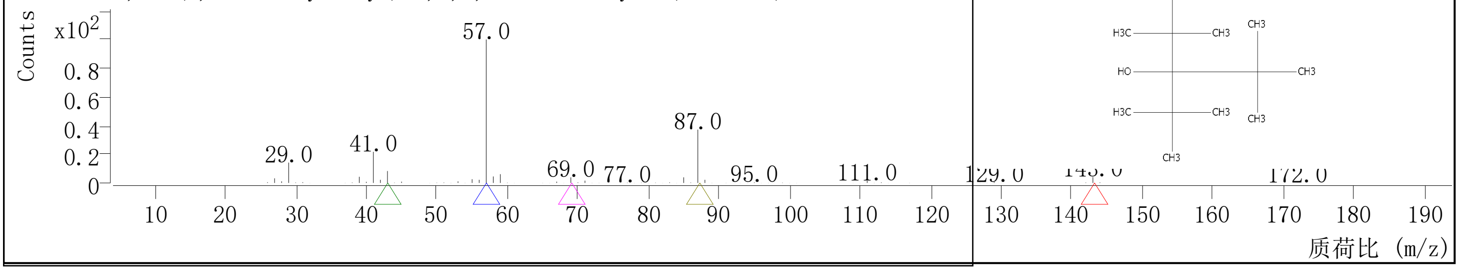

RT	化合物名称	CAS#	分子式	面积	匹配分数	样品	样品
12.9915	2-Butanone, 3-hydroxy-	513-86-0	C4H8O2	230645	94.7	0.35	1.60

2-Butanone, 3-hydroxy- (NIST08.L)

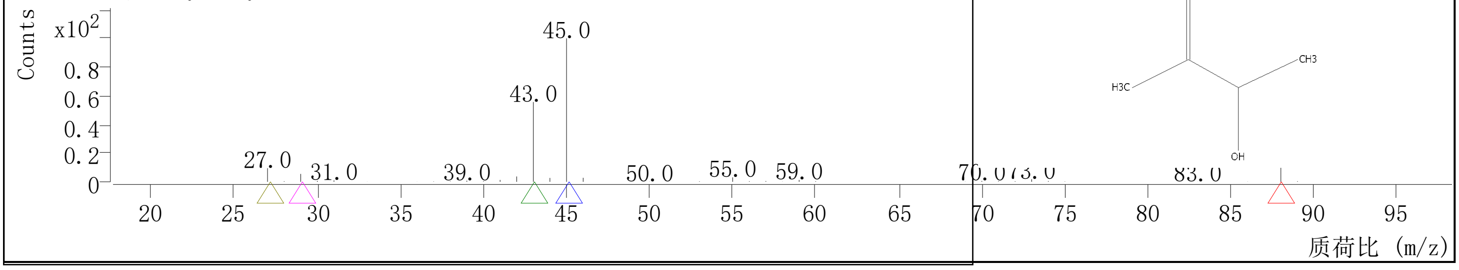

RT	化合物名称	CAS#	分子式	面积	匹配分数	样品	样品
13.8002	Pyrazine, 2,6-dimethyl-	108-50-9	C6H8N2	214248	70.6	0.33	1.49

Pyrazine, 2,6-dimethyl- (NIST08.L)

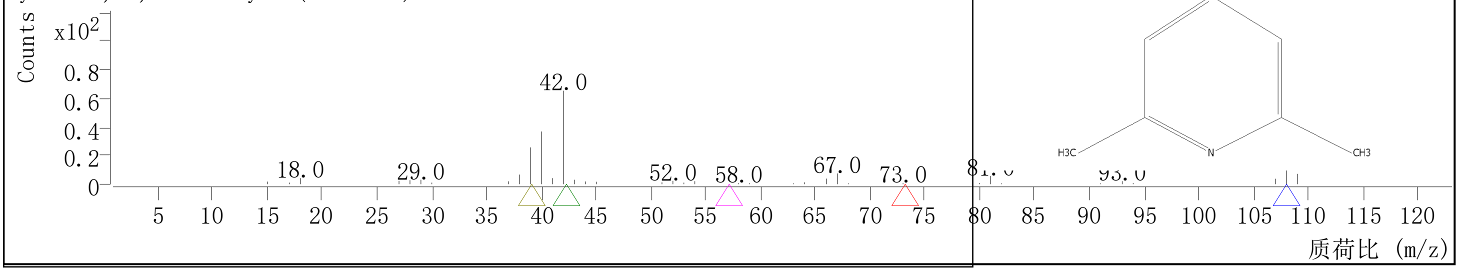

RT	化合物名称	CAS#	分子式	面积	匹配分数	样品	样品
14.2802	Cyclohexasiloxane, dodecamethyl-	540-97-6	C12H36O6Si6	6809135	95.6	10.42	47.24

Cyclohexasiloxane, dodecamethyl- (NIST08.L)

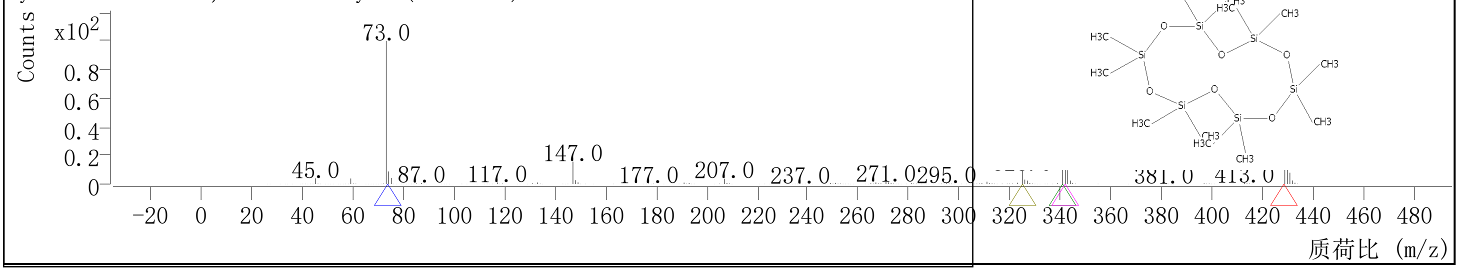

RT	化合物名称	CAS#	分子式	面积	匹配分数	样品	样品
15.0824	2-Nonanone	821-55-6	C9H18O	222171	90.3	0.34	1.54

2-Nonanone (NIST08.L)

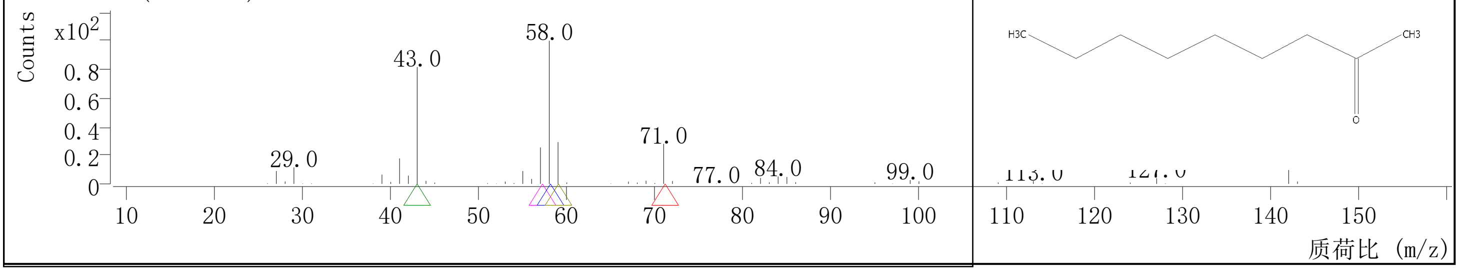

RT	化合物名称	CAS#	分子式	面积	匹配分数	样品	样品
15.1219	2,5-Dimethylhexane-2,5-dihydroperoxide	3025-88-5	C8H18O4	1483191	70.6	2.27	10.29

2,5-Dimethylhexane-2,5-dihydroperoxide (NIST08.L)

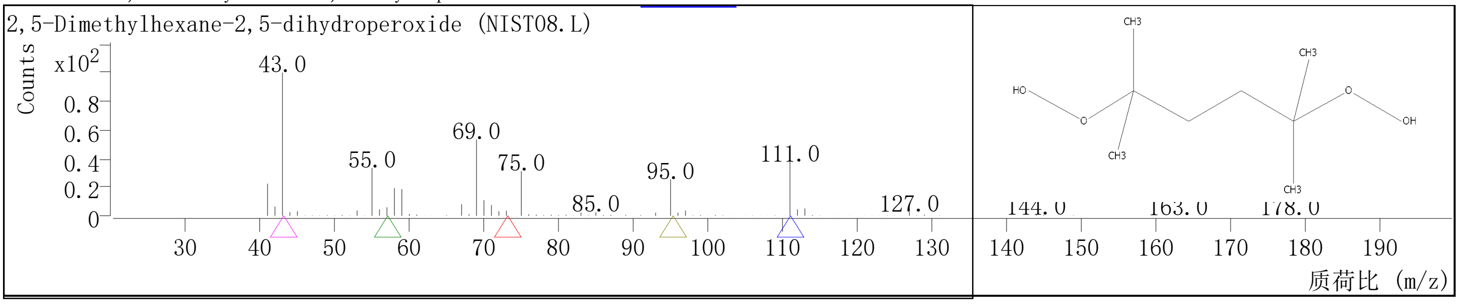

RT	化合物名称	CAS#	分子式	面积	匹配分数	样品	样品
15.9372	Pyrazine, 3-ethyl-2,5-dimethyl-	13360-65-1	C8H12N2	154958	86.6	0.24	1.07

Pyrazine, 3-ethyl-2,5-dimethyl- (NIST08.L)

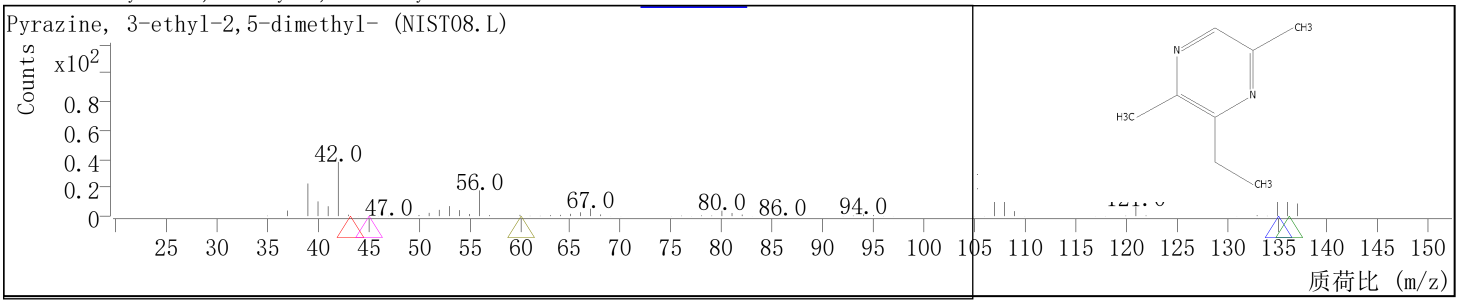

RT	化合物名称	CAS#	分子式	面积	匹配分数	样品	样品
16.4172	2H-Pyranmethanol, tetrahydro-2,5-dimethyl-	54004-46-5	C8H16O2	186013	84.4	0.28	1.29

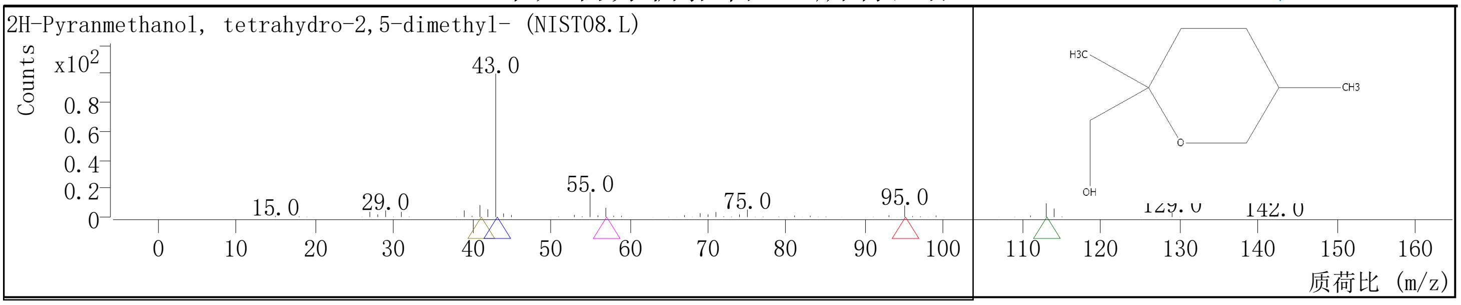

RT	化合物名称	CAS#	分子式	面积	匹配分数	样品	样品
16.5947	Cycloheptasiloxane, tetradecamethyl-	107-50-6	C14H42O7Si7	1709938	97.5	2.62	11.86

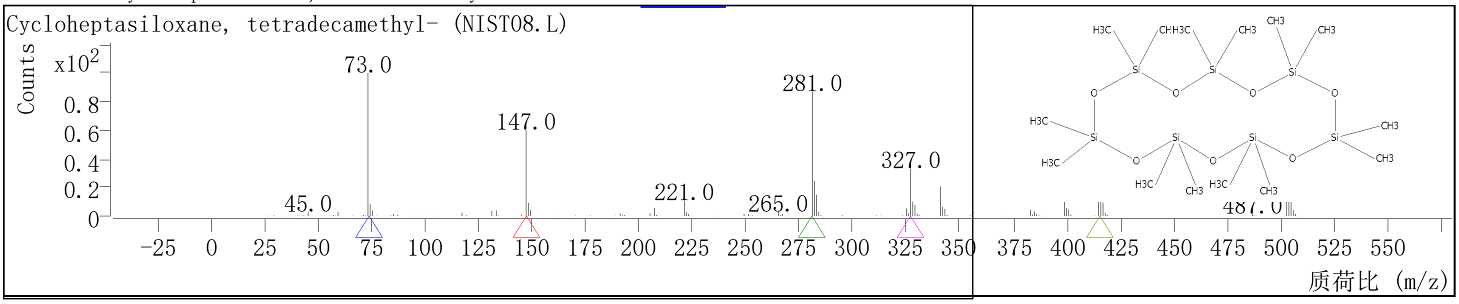

RT	化合物名称	CAS#	分子式	面积	匹配分数	样品	样品
16.7591	Vanillin, tert-butyldimethylsilyl ether	1000352-84-6	C14H22O3Si	296295	78.0	0.45	2.06

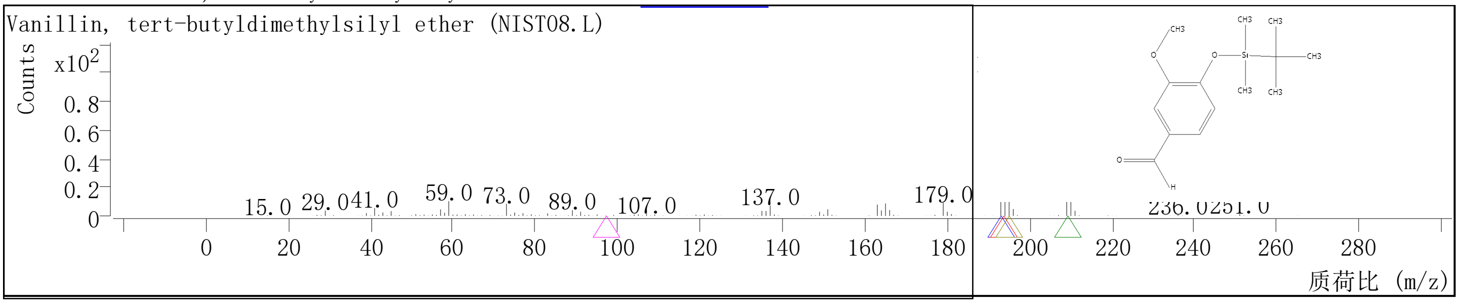

RT	化合物名称	CAS#	分子式	面积	匹配分数	样品	样品
16.9695	Benzaldehyde, 2,5-bis[(trimethylsilyl)oxy]-	56114-69-3	C13H22O3Si2	320813	75.6	0.49	2.23

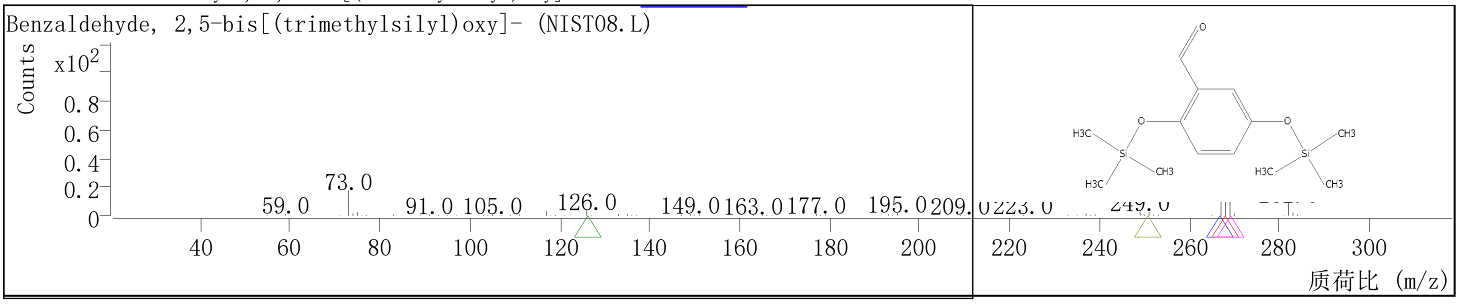

RT	化合物名称	CAS#	分子式	面积	匹配分数	样品	样品
17.2852	2,5-Dimethylhexane-2,5-dihydroperoxide	3025-88-5	C8H18O4	128236	76.5	0.20	0.89

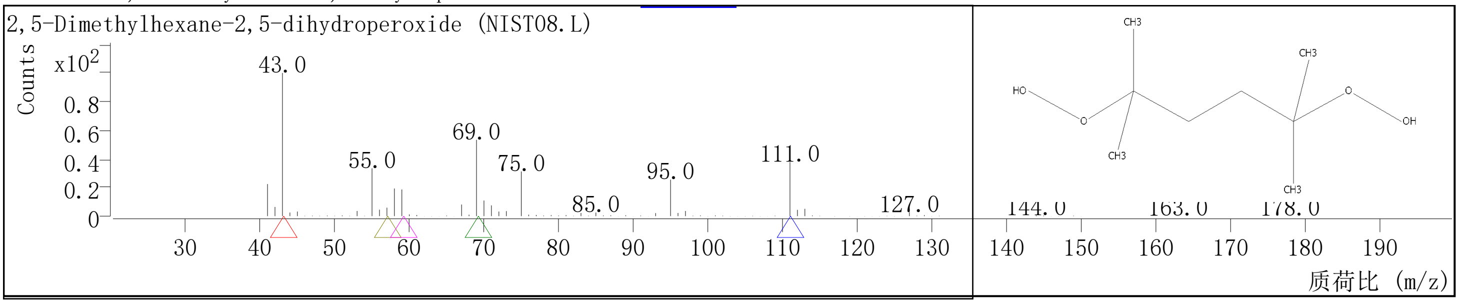

RT	化合物名称	CAS#	分子式	面积	匹配分数	样品	样品
17.4495	Silane, dimethyl(dimethyl(dimethyl(2-isopropylphenoxy)silyloxy)silyloxy)(2-isopropylphenoxy)-	1000347-25-6	C24H40O4Si3	105164	62.6	0.16	0.73

Agilent Trusted Answers

Silane, dimethyl(dimethyl(dimethyl(2-isopropylphenoxy)silyloxy)silyloxy)(2-isopro

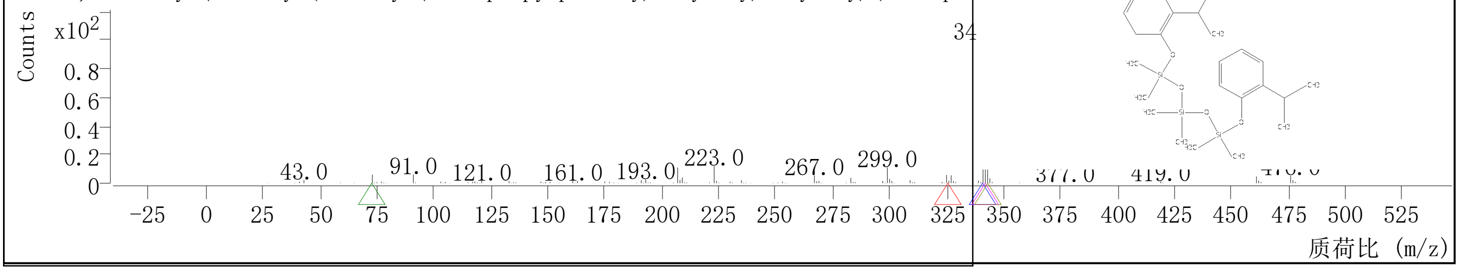

RT	化合物名称	CAS#	分子式	面积	匹配分数	样品	样品
18.3504	Cyclooctasiloxane, hexadecamethyl-	556-68-3	C16H48O8Si8	867367	93.0	1.33	6.02

Cyclooctasiloxane, hexadecamethyl- (NIST08.L)

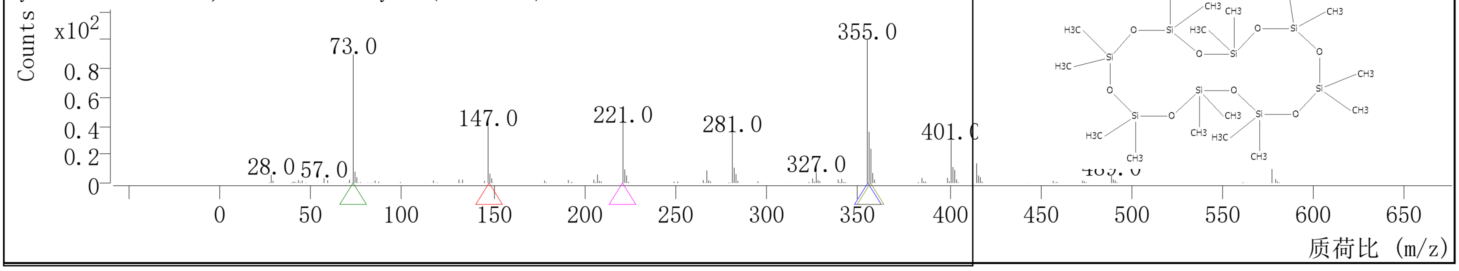

RT	化合物名称	CAS#	分子式	面积	匹配分数	样品	样品
18.5542	Ethanone, 1-phenyl-2-(1-piperidinyl)-	779-52-2	C13H17NO	153488	68.1	0.23	1.06

Ethanone, 1-phenyl-2-(1-piperidinyl)- (NIST08.L)

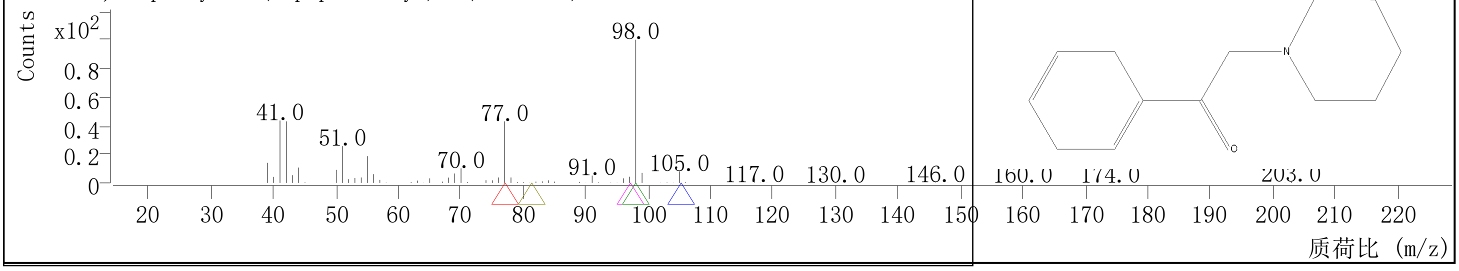

RT	化合物名称	CAS#	分子式	面积	匹配分数	样品	样品
19.2183	2-Undecanol	1653-30-1	C11H24O	166978	89.0	0.26	1.16

2-Undecanol (NIST08.L)

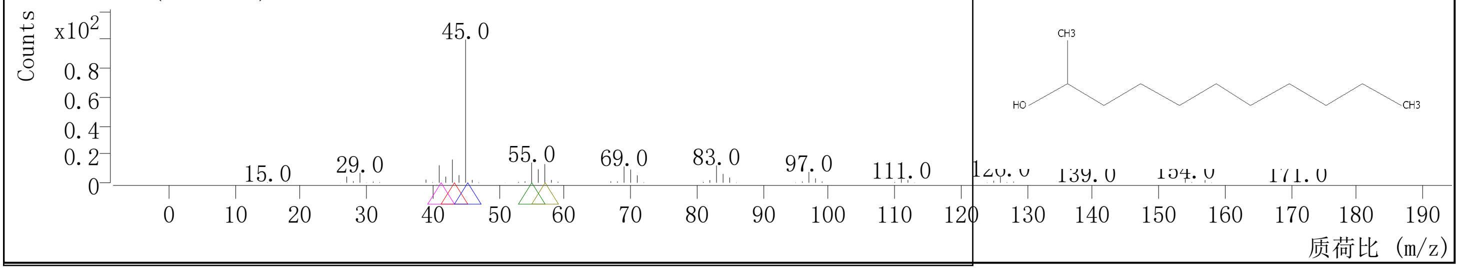

RT	化合物名称	CAS#	分子式	面积	匹配分数	样品	样品
19.4353	Oxime-, methoxy-phenyl-_	1000222-86-6	C8H9NO2	769810	83.3	1.18	5.34

Oxime-, methoxy-phenyl-_ (NIST08.L)

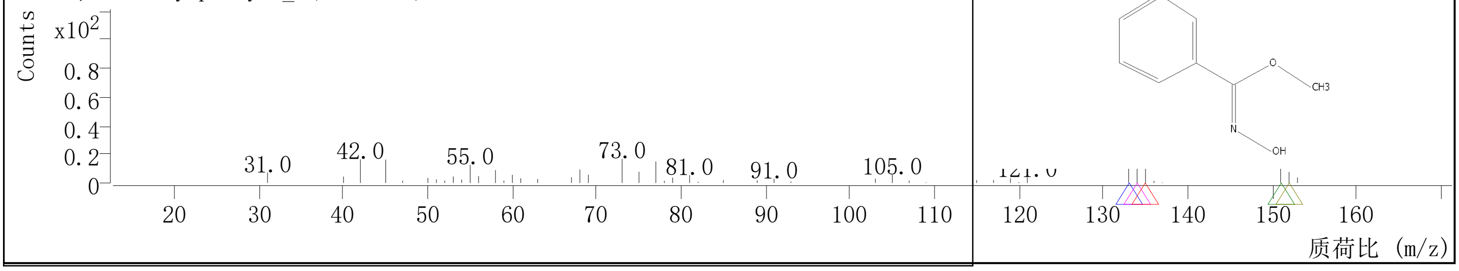

RT	化合物名称	CAS#	分子式	面积	匹配分数	样品	样品
19.6260	Aniline	62-53-3	C6H7N	155906	87.6	0.24	1.08

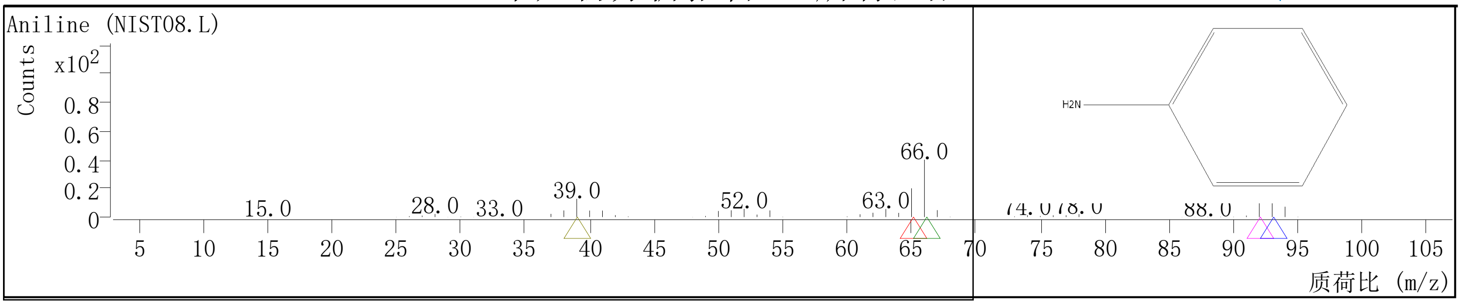

RT	化合物名称	CAS#	分子式	面积	匹配分数	样品	样品
19.8429	Cyclononasiloxane, octadecamethyl-	556-71-8	C18H54O9Si9	188298	92.5	0.29	1.31

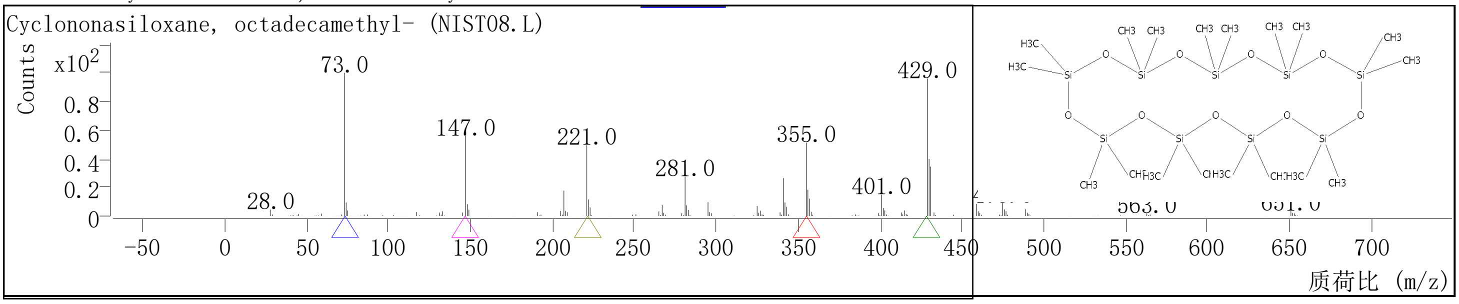

RT	化合物名称	CAS#	分子式	面积	匹配分数	样品	样品
19.8824	Cyclotrisiloxane, hexamethyl-	541-05-9	C6H18O3Si3	309389	88.8	0.47	2.15

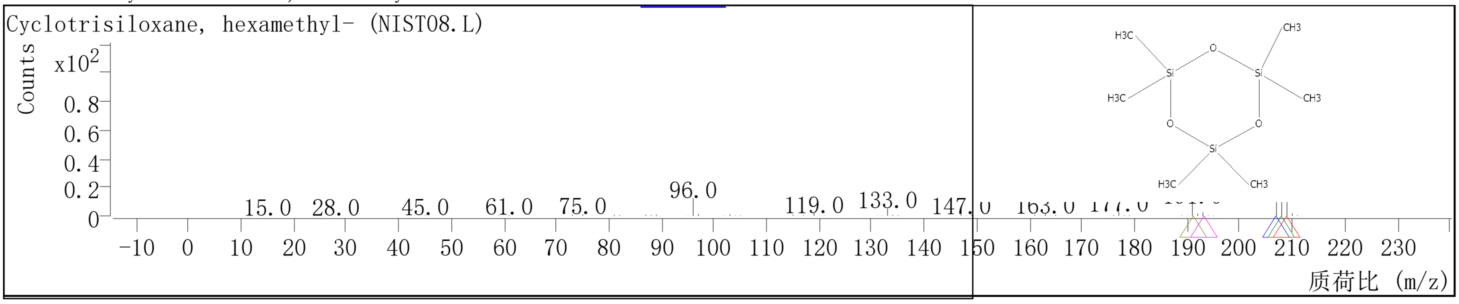

RT	化合物名称	CAS#	分子式	面积	匹配分数	样品	样品
20.2638	Tridecanal	10486-19-8	C13H26O	183396	90.3	0.28	1.27

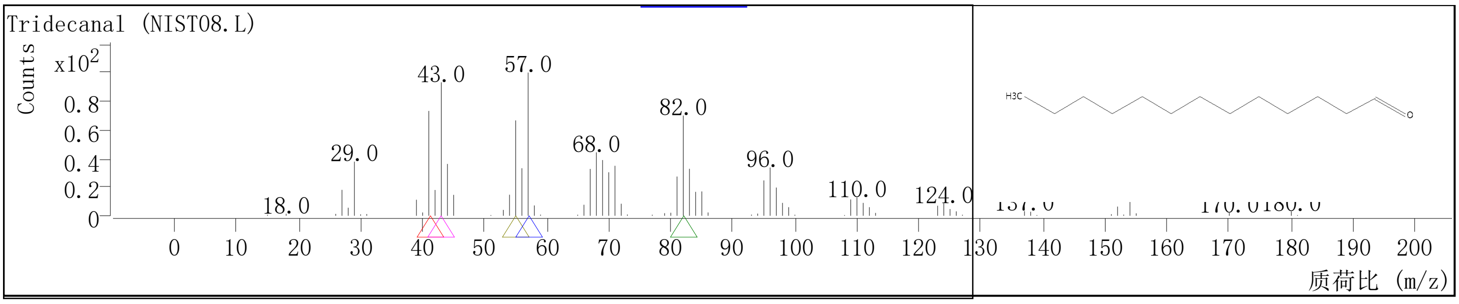

RT	化合物名称	CAS#	分子式	面积	匹配分数	样品	样品
20.4216	Cyclotetrasiloxane, octamethyl-	556-67-2	C8H24O4Si4	156970	93.9	0.24	1.09

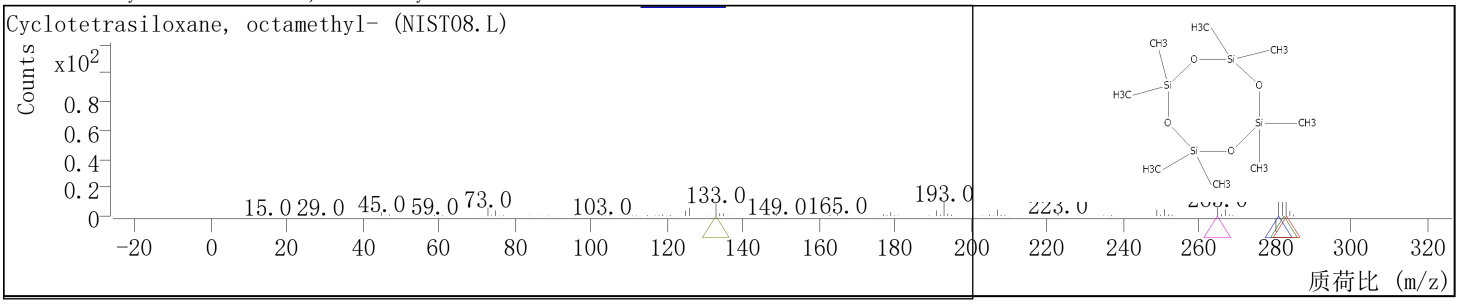

RT	化合物名称	CAS#	分子式	面积	匹配分数	样品	样品
20.8490	2-Tetradecanone	2345-27-9	C14H28O	116077	84.0	0.18	0.81

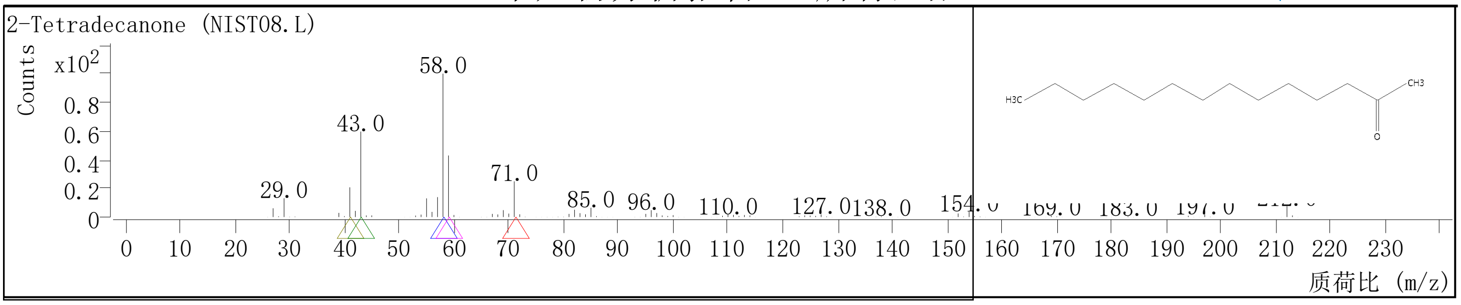

RT	化合物名称	CAS#	分子式	面积	匹配分数	样品	样品
21.0988	Cyclopentasiloxane, decamethyl-	541-02-6	C10H30O5Si5	322322	63.6	0.49	2.24

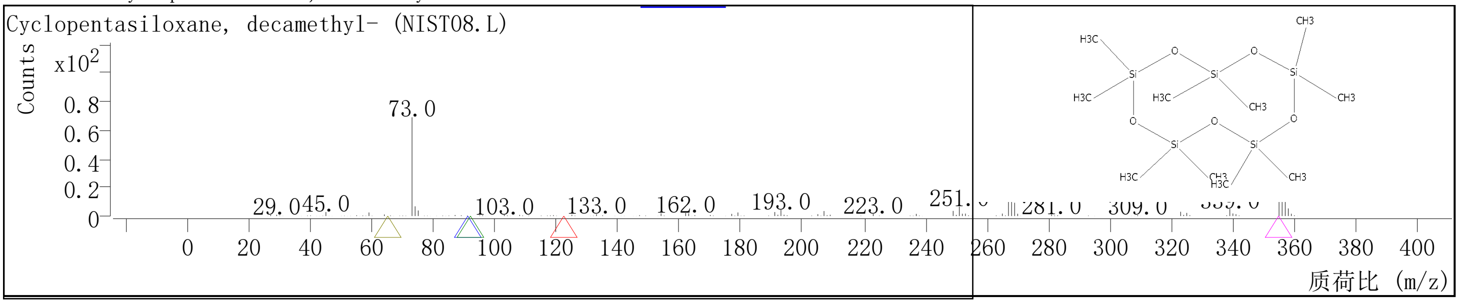

RT	化合物名称	CAS#	分子式	面积	匹配分数	样品	样品
21.1514	Cyclodecasiloxane, eicosamethyl-	18772-36-6	C20H60O10Si10	158519	75.1	0.24	1.10

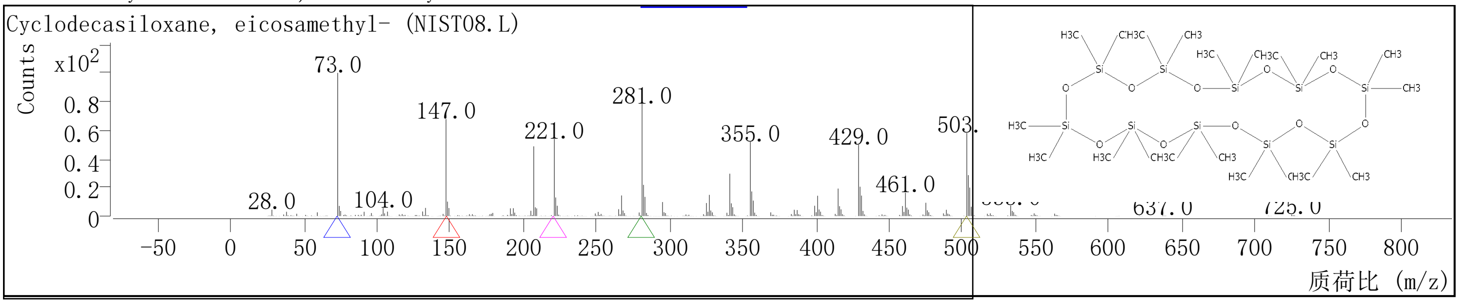

RT	化合物名称	CAS#	分子式	面积	匹配分数	样品	样品
21.2895	Benzyl nitrile	140-29-4	C8H7N	134710	94.0	0.21	0.93

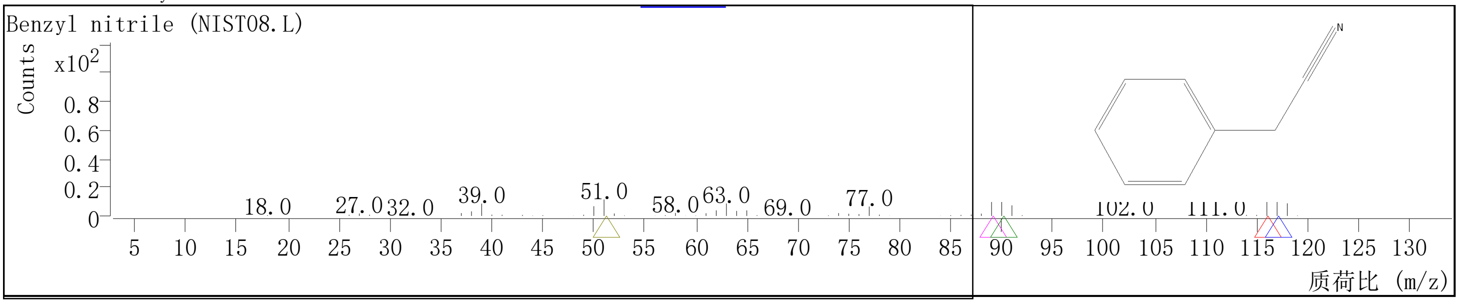

RT	化合物名称	CAS#	分子式	面积	匹配分数	样品	样品
22.1706	Oxirane, hexadecyl-	7390-81-0	C18H36O	174284	87.4	0.27	1.21

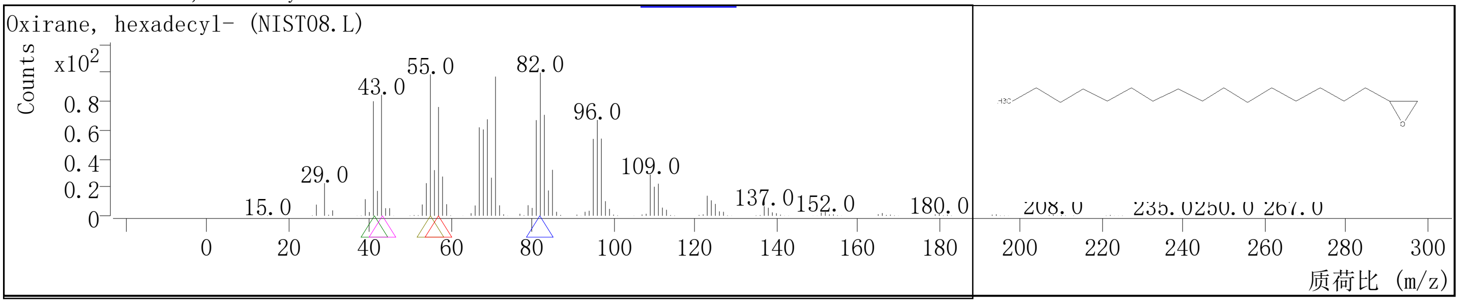

RT	化合物名称	CAS#	分子式	面积	匹配分数	样品	样品
22.3481	Nonanoic acid, 9-oxo-, methyl ester	1931-63-1	C10H18O3	747948	96.8	1.14	5.19

Nonanoic acid, 9-oxo-, methyl ester (NIST08.L)

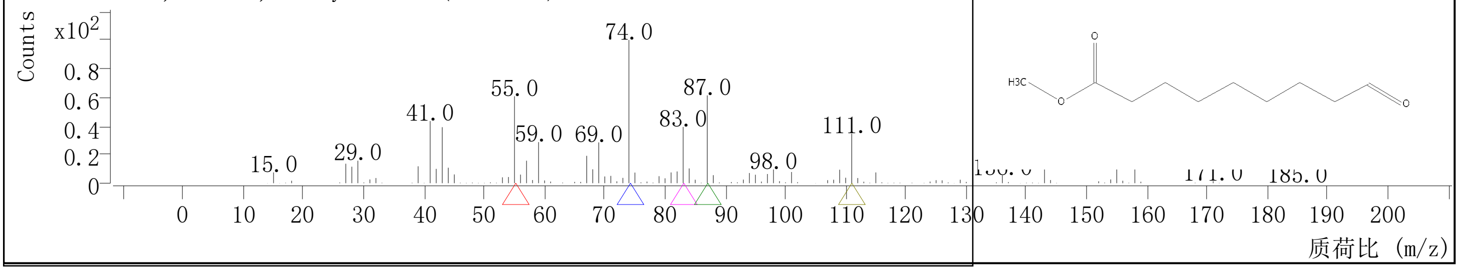

RT	化合物名称	CAS#	分子式	面积	匹配分数	样品	样品
23.2753	2-Undecenoic acid	4189-02-0	C11H20O2	100163	61.7	0.15	0.69

2-Undecenoic acid (NIST08.L)

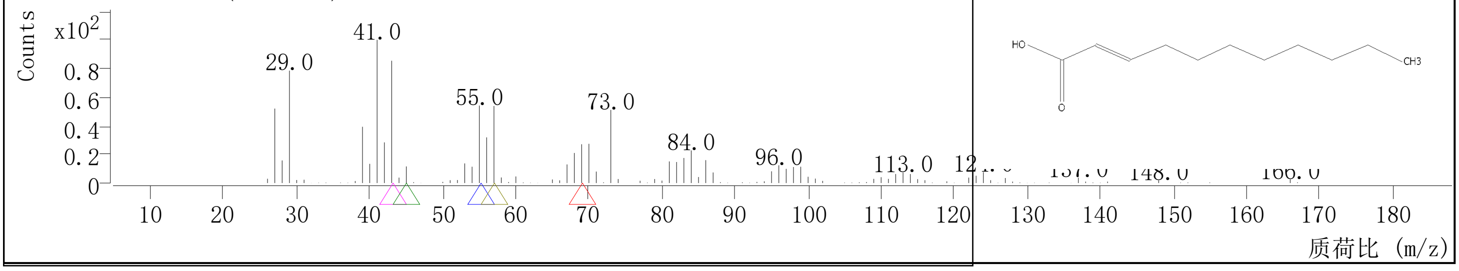

RT	化合物名称	CAS#	分子式	面积	匹配分数	样品	样品
23.7027	Hexadecanoic acid, methyl ester	112-39-0	C17H34O2	5367558	98.4	8.21	37.24

Hexadecanoic acid, methyl ester (NIST08.L)

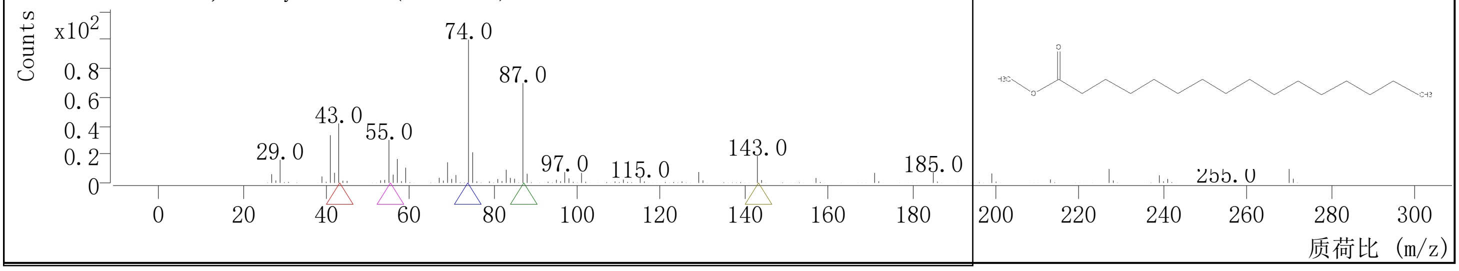

RT	化合物名称	CAS#	分子式	面积	匹配分数	样品	样品
23.9328	9-Hexadecenoic acid, methyl ester, (Z)-	1120-25-8	C17H32O2	192296	89.9	0.29	1.33

9-Hexadecenoic acid, methyl ester, (Z)- (NIST08.L)

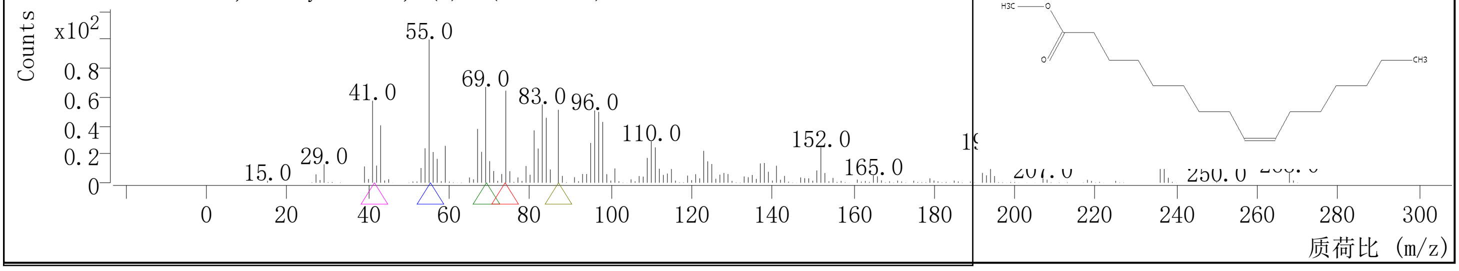

RT	化合物名称	CAS#	分子式	面积	匹配分数	样品	样品
23.9985	Hexadecanoic acid, ethyl ester	628-97-7	C18H36O2	132458	89.7	0.20	0.92

Hexadecanoic acid, ethyl ester (NIST08.L)

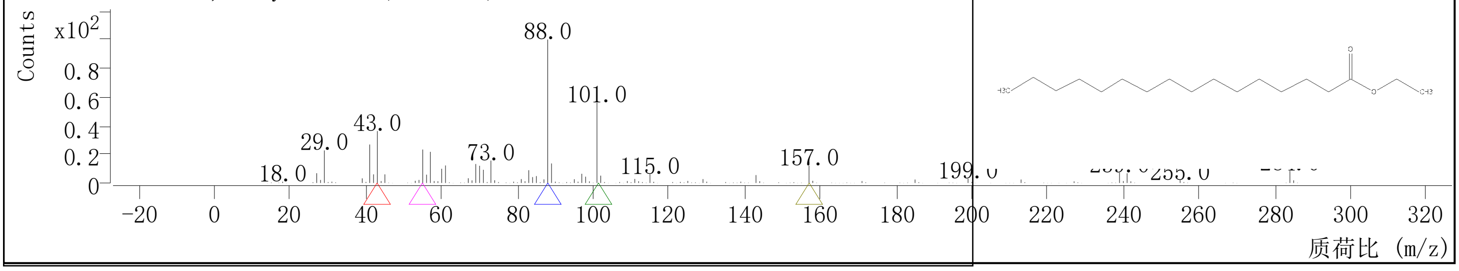

RT	化合物名称	CAS#	分子式	面积	匹配分数	样品	样品
24.3536	Phenol, 2,4-bis(1,1-dimethylethyl)-	96-76-4	C14H22O	101156	85.2	0.15	0.70

Phenol, 2,4-bis(1,1-dimethylethyl)- (NIST08.L)

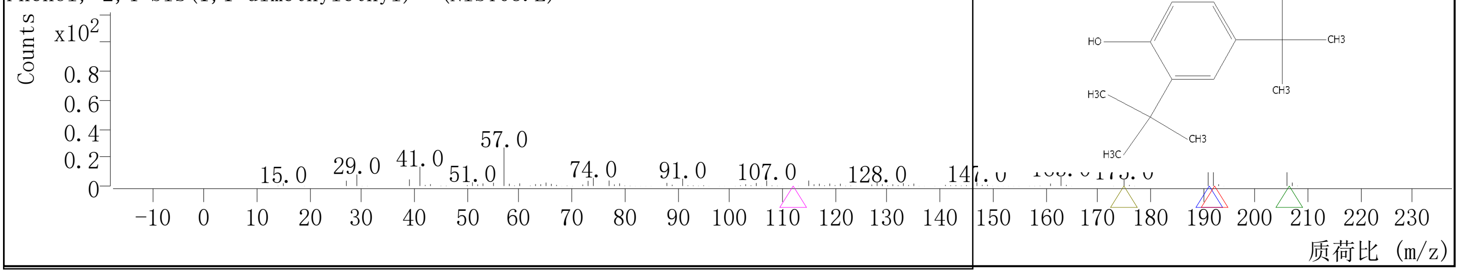

RT	化合物名称	CAS#	分子式	面积	匹配分数	样品	样品
25.5437	Octadecanoic acid, methyl ester	112-61-8	C19H3802	2103302	98.0	3.22	14.59

Octadecanoic acid, methyl ester (NIST08.L)

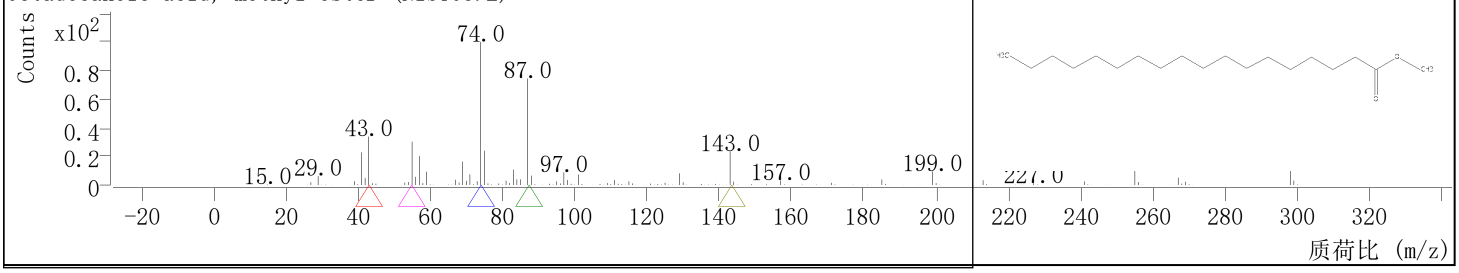

RT	化合物名称	CAS#	分子式	面积	匹配分数	样品	样品
25.7804	9-Octadecenoic acid (Z)-, methyl ester	112-62-9	C19H3602	4271181	99.5	6.53	29.63

9-Octadecenoic acid (Z)-, methyl ester (NIST08.L)

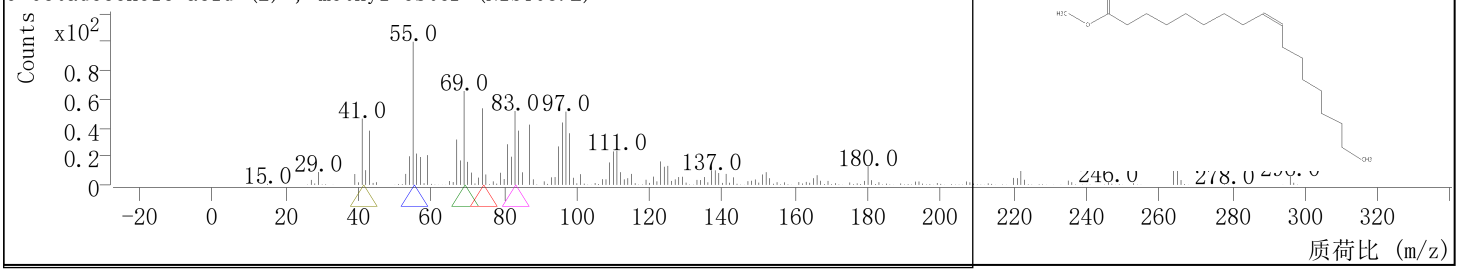

RT	化合物名称	CAS#	分子式	面积	匹配分数	样品	样品
25.8528	9-Octadecenoic acid (Z)-, methyl ester	112-62-9	C19H3602	267769	79.6	0.41	1.86

9-Octadecenoic acid (Z)-, methyl ester (NIST08.L)

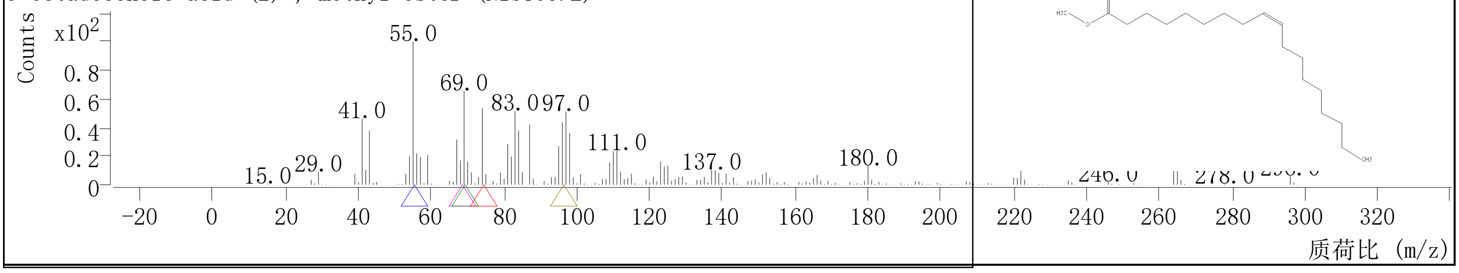

RT	化合物名称	CAS#	分子式	面积	匹配分数	样品	样品
26.1487	Ethyl Oleate	111-62-6	C20H3802	135663	80.2	0.21	0.94

Ethyl Oleate (NIST08.L)

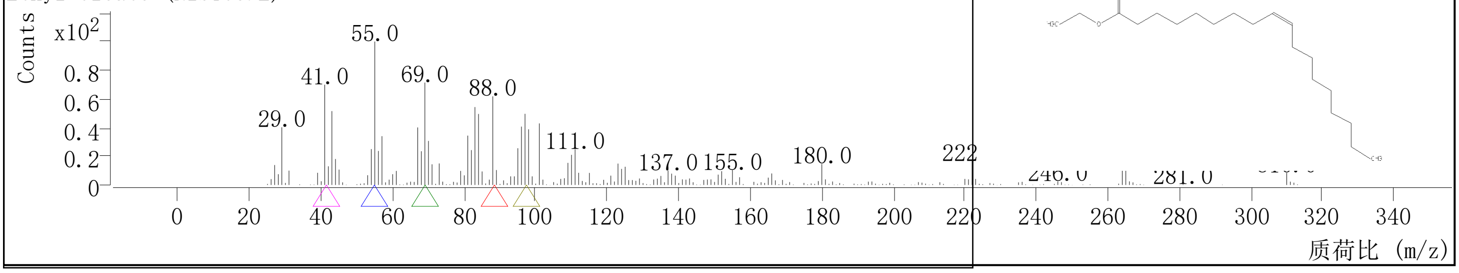

RT	化合物名称	CAS#	分子式	面积	匹配分数	样品	样品
26.3065	9,12-Octadecadienoic acid (Z,Z)-, methyl ester	112-63-0	C19H3402	692797	96.5	1.06	4.81

9,12-Octadecadienoic acid (Z,Z)-, methyl ester (NIST08.L)

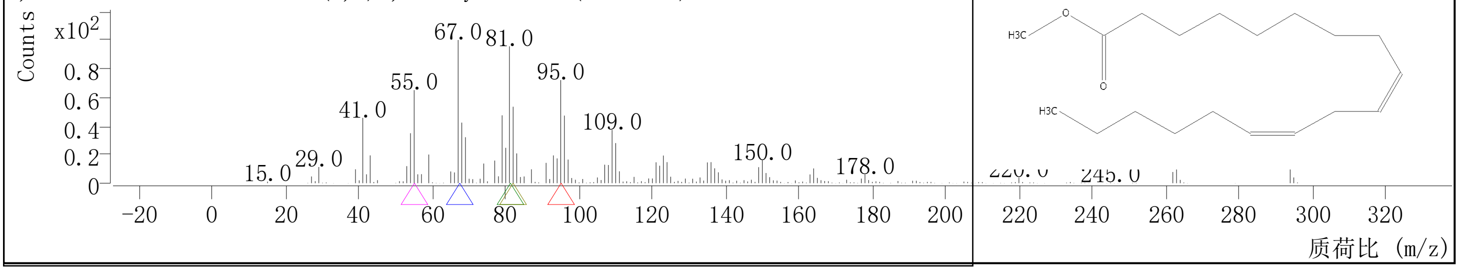

RT	化合物名称	CAS#	分子式	面积	匹配分数	样品	样品
29.0155	1,2-Benzenedicarboxylic acid, butyl 2-methylpropyl ester	17851-53-5	C16H22O4	377065	74.5	0.58	2.62

1,2-Benzenedicarboxylic acid, butyl 2-methylpropyl ester (NIST08.L)

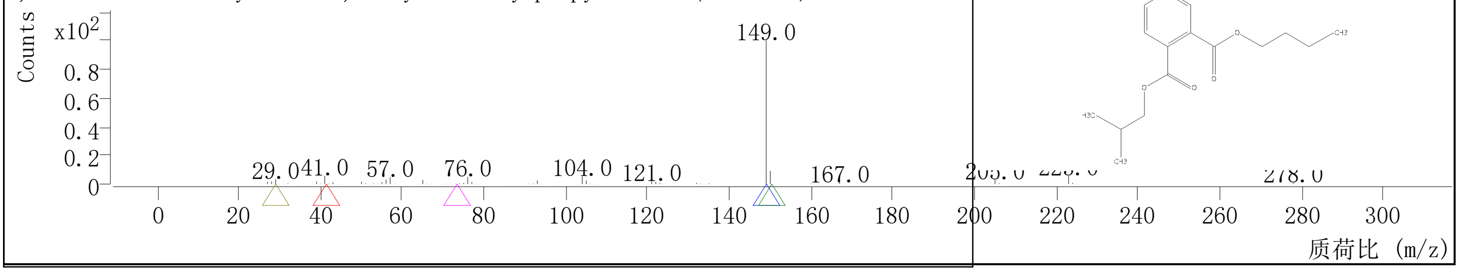

RT	化合物名称	CAS#	分子式	面积	匹配分数	样品	样品
30.9486	Pentadecanoic acid	1002-84-2	C15H30O2	101920	68.6	0.16	0.71

Pentadecanoic acid (NIST08.L)

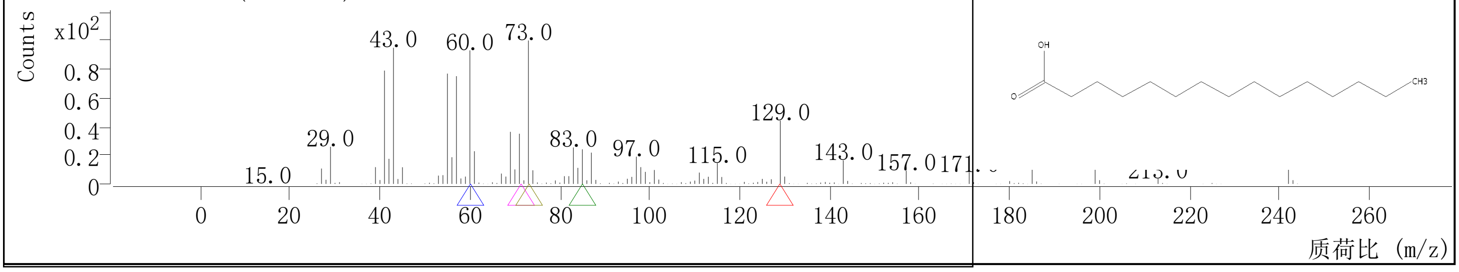

RT	化合物名称	CAS#	分子式	面积	匹配分数	样品	样品
33.5656	n-Hexadecanoic acid	57-10-3	C16H32O2	1698163	88.1	2.60	11.78

n-Hexadecanoic acid (NIST08.L)

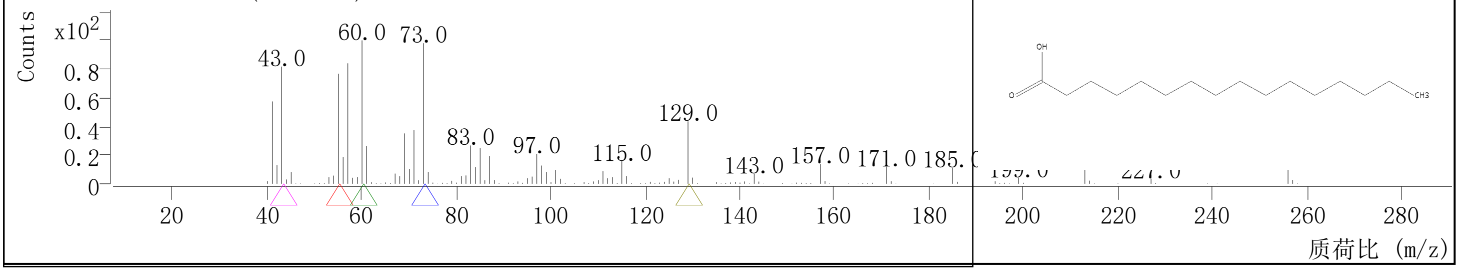

RT	化合物名称	CAS#	分子式	面积	匹配分数	样品	样品
34.6111	cis-9-Hexadecenoic acid	1000333-19-5	C16H30O2	386794	61.6	0.59	2.68

cis-9-Hexadecenoic acid (NIST08.L)

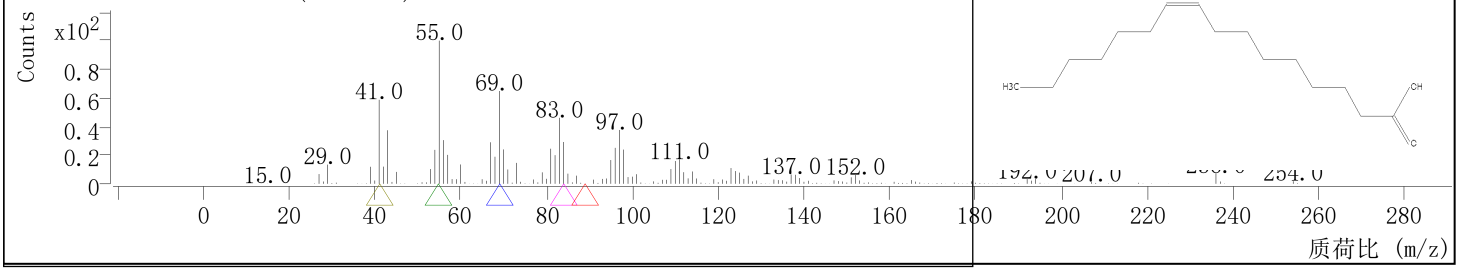

RT	化合物名称	CAS#	分子式	面积	匹配分数	样品	样品
39.3848	Geranylgeraniol	24034-73-9	C20H34O	242983	68.9	0.37	1.69

Agilent | Trusted Answers

Geranylgeraniol (NIST08.L)

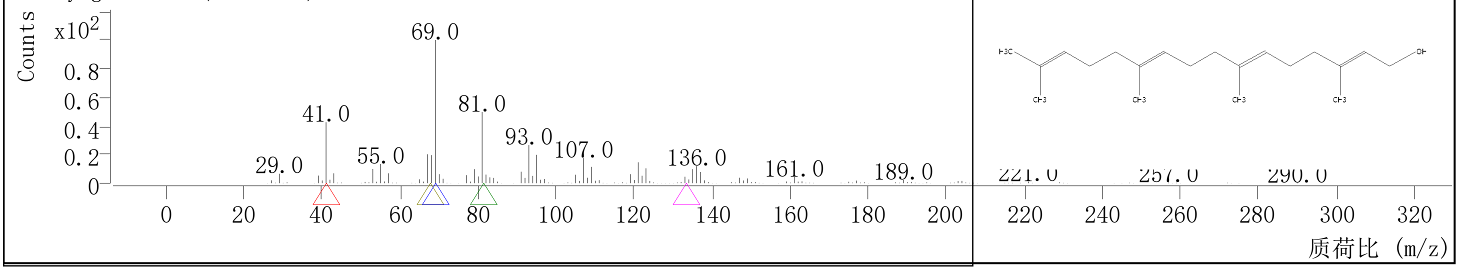

Supplement: Supplementary Chromatogram 1 — Chromatographic results of VOCs produced by B. subtilis GB519. [file Data_Sheet_1.pdf]
