# Supplementary material for: Antifungal activity of volatile organic compounds produced by Bacillus subtilis GB519 against blast pathogen Magnaporthe oryzae in rice
Source: Front Microbiol. 2026 Mar 11;17:1757473. doi: 10.3389/fmicb.2026.1757473 (PMC13013540; doi:10.3389/fmicb.2026.1757473)

Agilent Trusted Answers

批处理路径	F:\20241203-		
分析文件名称	TEST.uaf		
分析员姓名	5977		
分析时间	2024/12/5 8:02:29		
数据文件名称	1-空白.D	数据路径名称	F:\20241203-
样品名称	Sample Run on ChemStation	样品类型	样品
采集方法文件	HS.M	采集方法路径	
采集时间	2024/12/3 18:15:23	操作员	MassHunter GC/MS Translator
仪器名称	Instrument #1	稀释	1

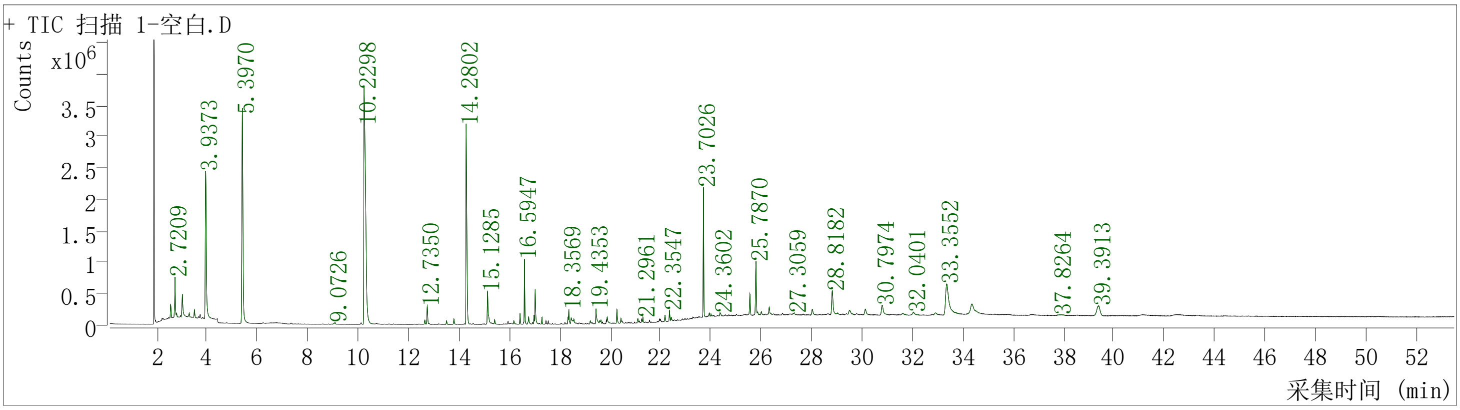

RT	化合物名称	CAS#	分子式	面积	匹配分数	样品	样品
2.5499	Di-tert-butyl peroxide	110-05-4	C8H18O2	427112	95.3	0.55	2.31
2.7209	Acetone	67-64-1	C3H6O	1225513	98.9	1.59	6.64
2.7866	Oxetane, 2,2-dimethyl-	6245-99-4	C5H10O	102906	83.9	0.13	0.56
3.0167	Cyclotrisiloxane, hexamethyl-	541-05-9	C6H18O3Si3	1085709	98.5	1.41	5.88
3.2863	Pentane, 2,3,4-trimethyl-	565-75-3	C8H18	173079	80.9	0.22	0.94
3.4902	2-Propanol, 2-methyl-	75-65-0	C4H10O	237952	93.6	0.31	1.29
3.9373	Methylene Chloride	75-09-2	CH2Cl2	7150843	98.1	9.28	38.72
5.3970	Cyclotetrasiloxane, octamethyl-	556-67-2	C8H24O4Si4	10309295	96.4	13.38	55.83
9.0726	Orthoformic acid, triisobutyl ester	16754-49-7	C13H28O3	109467	78.6	0.14	0.59
10.2298	Cyclopentasiloxane, decamethyl-	541-02-6	C10H30O5Si5	18466574	98.3	23.97	100.00
12.6430	dl-Metanephrine, bis(trimethylsilyl) ether	1000334-02-2	C16H31NO3Si2	146087	58.4	0.19	0.79
12.7350	3-Pentanol, 3-(1,1-dimethylethyl)-2,2,4,4-tetramethyl-	41902-42-5	C13H28O	652862	78.7	0.85	3.54
13.5044	2-tert-Butoxytetrahydrofuran	1927-59-9	C8H16O2	117635	79.6	0.15	0.64
13.7937	Oxalic acid, dodecyl 2-methylphenyl ester	1000309-60-2	C21H32O4	188388	61.5	0.24	1.02
14.2802	Cyclohexasiloxane, dodecamethyl-	540-97-6	C12H36O6Si6	7844008	95.6	10.18	42.48
15.1285	2,5-Dimethylhexane-2,5-dihydroperoxide	3025-88-5	C8H18O4	1455811	77.6	1.89	7.88
15.4112	2H-Pyranmethanol, tetrahydro-2,5-dimethyl-	54004-46-5	C8H16O2	153233	86.0	0.20	0.83
16.1739	Furfural	98-01-1	C5H4O2	136374	92.6	0.18	0.74
16.4172	2H-Pyranmethanol, tetrahydro-2,5-dimethyl-	54004-46-5	C8H16O2	273070	85.4	0.35	1.48
16.5947	Cycloheptasiloxane, tetradecamethyl-	107-50-6	C14H42O7Si7	1583220	97.3	2.05	8.57
16.7591	Vanillin, tert-butyldimethylsilyl ether	1000352-84-6	C14H22O3Si	306040	76.9	0.40	1.66
16.9695	Benzaldehyde, 2,5-bis[(trimethylsilyl)oxy]-	56114-69-3	C13H22O3Si2	224171	77.1	0.29	1.21
17.0221	Benzaldehyde	100-52-7	C7H6O	1115975	99.0	1.45	6.04
17.2917	2,5-Dimethylhexane-2,5-dihydroperoxide	3025-88-5	C8H18O4	171254	73.7	0.22	0.93
18.3240	Silanediol, dimethyl-	1066-42-8	C2H8O2Si	157878	50.4	0.20	0.85
18.3569	Cyclooctasiloxane, hexadecamethyl-	556-68-3	C16H48O8Si8	386211	93.2	0.50	2.09
18.4490	Coumarin, 6-benzyloxy-3,4-dihydro-4,4-dimethyl-5-nitro-	1000129-25-7	C18H17NO5	416586	61.3	0.54	2.26
18.5542	2-Furanmethanol	98-00-0	C5H6O2	206061	66.9	0.27	1.12
19.2117	Dodecanal	112-54-9	C12H24O	108158	90.6	0.14	0.59
19.4353	Oxime-, methoxy-phenyl-_	1000222-86-6	C8H9NO2	673700	83.1	0.87	3.65

RT	化合物名称	CAS#	分子式	面积	匹配分数	样品	样品
19.5799	Naphthalene	91-20-3	C10H8	127249	92.1	0.17	0.69
19.6325	Aniline	62-53-3	C6H7N	113899	84.5	0.15	0.62
19.8824	Cyclotrisiloxane, hexamethyl-	541-05-9	C6H18O3Si3	292141	91.5	0.38	1.58
20.2638	Tridecanal	10486-19-8	C13H26O	456710	94.0	0.59	2.47
20.4216	Cyclotetrasiloxane, octamethyl-	556-67-2	C8H24O4Si4	151932	93.1	0.20	0.82
21.0988	Cyclopentasiloxane, decamethyl-	541-02-6	C10H30O5Si5	153387	79.2	0.20	0.83
21.2961	Benzyl nitrile	140-29-4	C8H7N	141765	92.2	0.18	0.77
22.1706	Oxirane, hexadecyl-	7390-81-0	C18H36O	210898	82.9	0.27	1.14
22.3547	Nonanoic acid, 9-oxo-, methyl ester	1931-63-1	C10H18O3	340533	94.5	0.44	1.84
22.4205	N-Acryloylmorpholine	5117-12-4	C7H11NO2	125225	80.6	0.16	0.68
23.7026	Hexadecanoic acid, methyl ester	112-39-0	C17H34O2	3335168	98.5	4.33	18.06
23.9328	9-Hexadecenoic acid, methyl ester, (Z)-	1120-25-8	C17H32O2	139253	87.9	0.18	0.75
23.9985	Hexadecanoic acid, ethyl ester	628-97-7	C18H36O2	109674	78.5	0.14	0.59
24.3602	Phenol, 2,4-bis(1,1-dimethylethyl)-	96-76-4	C14H22O	159086	86.3	0.21	0.86
25.5437	Octadecanoic acid, methyl ester	112-61-8	C19H38O2	741379	96.7	0.96	4.01
25.7870	9-Octadecenoic acid (Z)-, methyl ester	112-62-9	C19H36O2	2019870	98.9	2.62	10.94
25.8528	trans-13-Octadecenoic acid, methyl ester	1000333-61-3	C19H36O2	111184	67.0	0.14	0.60
25.9974	Dodecanoic acid	143-07-7	C12H24O2	123260	82.8	0.16	0.67
26.3130	9,12-Octadecadienoic acid (Z,Z)-, methyl ester	112-63-0	C19H34O2	372612	91.7	0.48	2.02
27.3059	E-14-Hexadecenal	330207-53-9	C16H30O	111513	73.8	0.14	0.60
28.0226	Tetradecanoic acid	544-63-8	C14H28O2	311432	86.9	0.40	1.69
28.8182	Tetradecanoic acid	544-63-8	C14H28O2	1487234	96.0	1.93	8.05
29.0286	Dibutyl phthalate	84-74-2	C16H22O4	109588	63.6	0.14	0.59
29.5021	Z-7-Tetradecenoic acid	1000130-98-4	C14H26O2	381210	79.5	0.49	2.06
30.1267	Pentadecanoic acid	1002-84-2	C15H30O2	398306	80.8	0.52	2.16
30.7974	Pentadecanoic acid	1002-84-2	C15H30O2	825709	92.2	1.07	4.47
31.6062	Cyclopentadecanone, 2-hydroxy-	4727-18-8	C15H28O2	146674	68.7	0.19	0.79
32.0401	n-Hexadecanoic acid	57-10-3	C16H32O2	272028	77.2	0.35	1.47
32.9081	cis-9-Hexadecenoic acid	1000333-19-5	C16H30O2	194297	71.7	0.25	1.05
33.3552	n-Hexadecanoic acid	57-10-3	C16H32O2	4787927	96.1	6.21	25.93
34.3678	cis-9-Hexadecenoic acid	1000333-19-5	C16H30O2	1599687	92.8	2.08	8.66
37.8264	.beta.-D-Mannofuranoside, farnesyl-	1000155-15-5	C21H36O6	177528	55.0	0.23	0.96
39.3913	Squalene	7683-64-9	C30H50	1409518	90.5	1.83	7.63

RT	化合物名称	CAS#	分子式	面积	匹配分数	样品	样品
2.5499	Di-tert-butyl peroxide	110-05-4	C8H18O2	427112	95.3	0.55	2.31

Di-tert-butyl peroxide (NIST08.L)

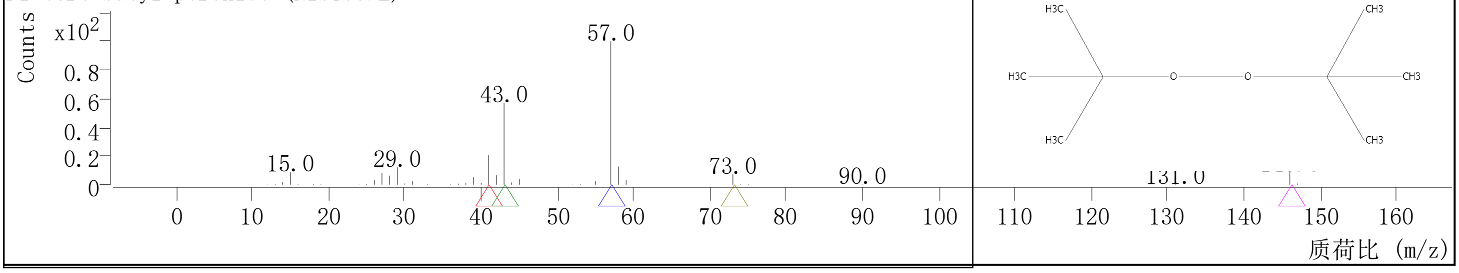

RT	化合物名称	CAS#	分子式	面积	匹配分数	样品	样品
2.7209	Acetone	67-64-1	C3H6O	1225513	98.9	1.59	6.64

Acetone (NIST08.L)

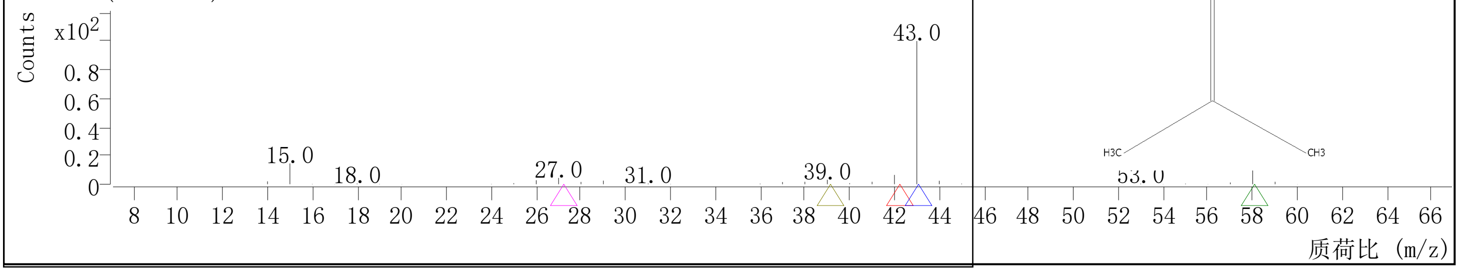

RT	化合物名称	CAS#	分子式	面积	匹配分数	样品	样品
2.7866	Oxetane, 2,2-dimethyl-	6245-99-4	C5H10O	102906	83.9	0.13	0.56

Oxetane, 2,2-dimethyl- (NIST08.L)

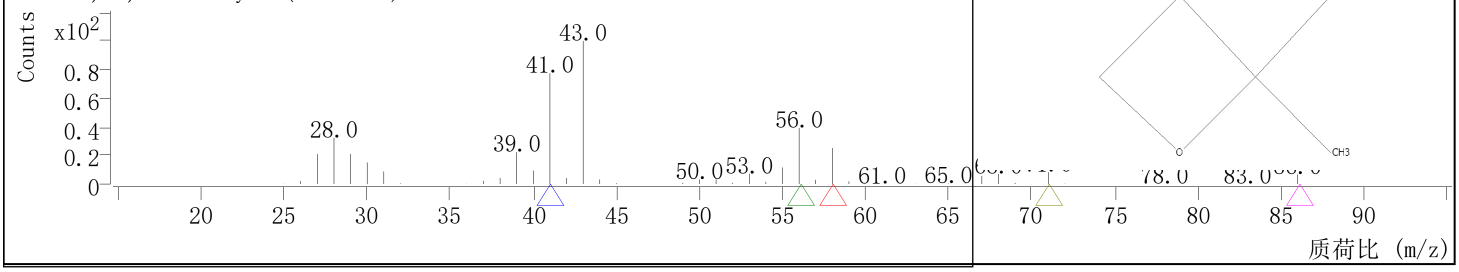

RT	化合物名称	CAS#	分子式	面积	匹配分数	样品	样品
3.0167	Cyclotrisiloxane, hexamethyl-	541-05-9	C6H18O3Si3	1085709	98.5	1.41	5.88

Cyclotrisiloxane, hexamethyl- (NIST08.L)

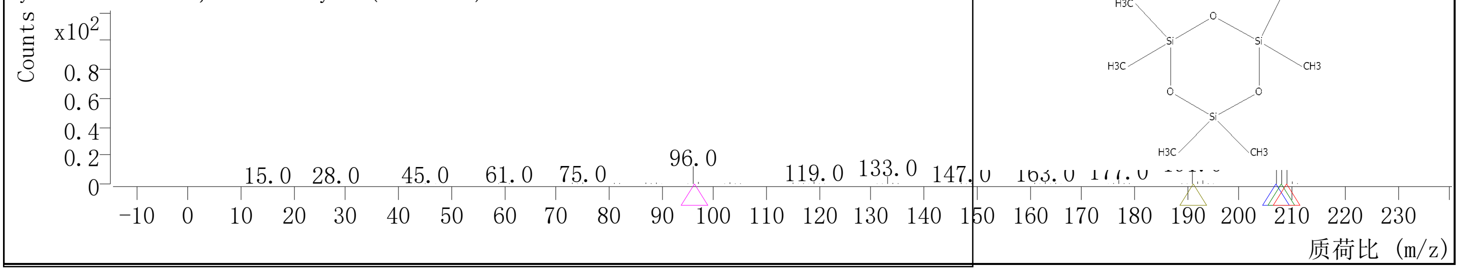

RT	化合物名称	CAS#	分子式	面积	匹配分数	样品	样品
3.2863	Pentane, 2,3,4-trimethyl-	565-75-3	C8H18	173079	80.9	0.22	0.94

Pentane, 2,3,4-trimethyl- (NIST08.L)

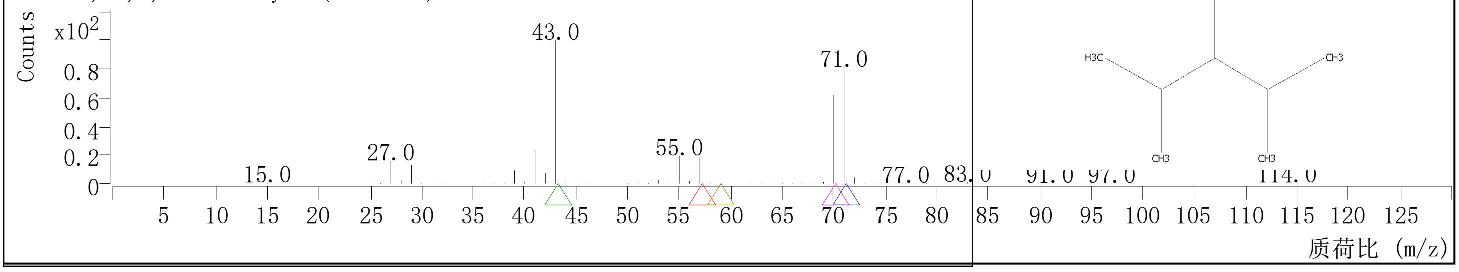

RT	化合物名称	CAS#	分子式	面积	匹配分数	样品	样品
3.4902	2-Propanol, 2-methyl-	75-65-0	C4H10O	237952	93.6	0.31	1.29

2-Propanol, 2-methyl- (NIST08.L)

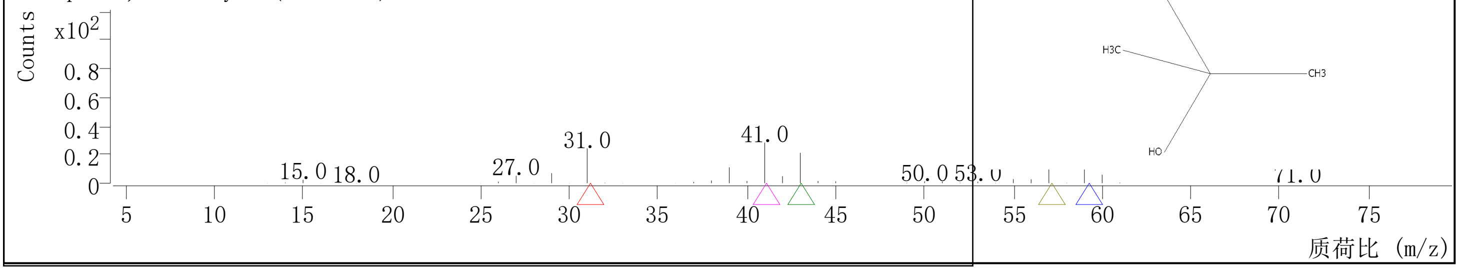

RT	化合物名称	CAS#	分子式	面积	匹配分数	样品	样品
3.9373	Methylene Chloride	75-09-2	CH2Cl2	7150843	98.1	9.28	38.72

Methylene Chloride (NIST08.L)

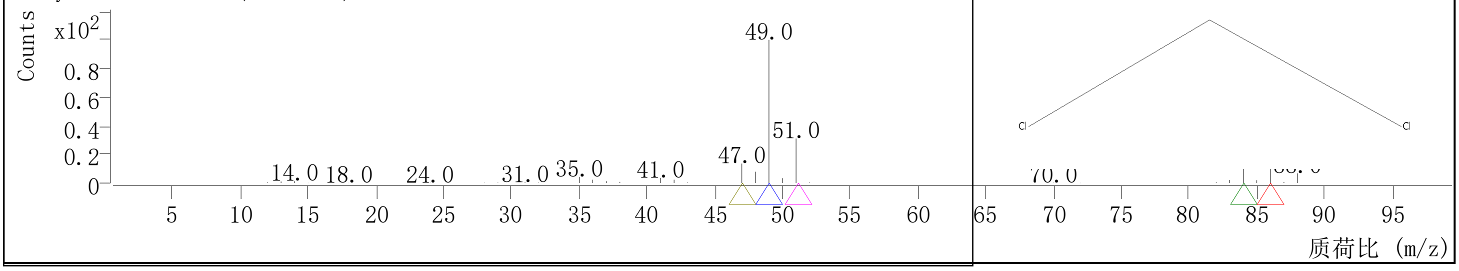

RT	化合物名称	CAS#	分子式	面积	匹配分数	样品	样品
5.3970	Cyclotetrasiloxane, octamethyl-	556-67-2	C8H24O4Si4	10309295	96.4	13.38	55.83

Cyclotetrasiloxane, octamethyl- (NIST08.L)

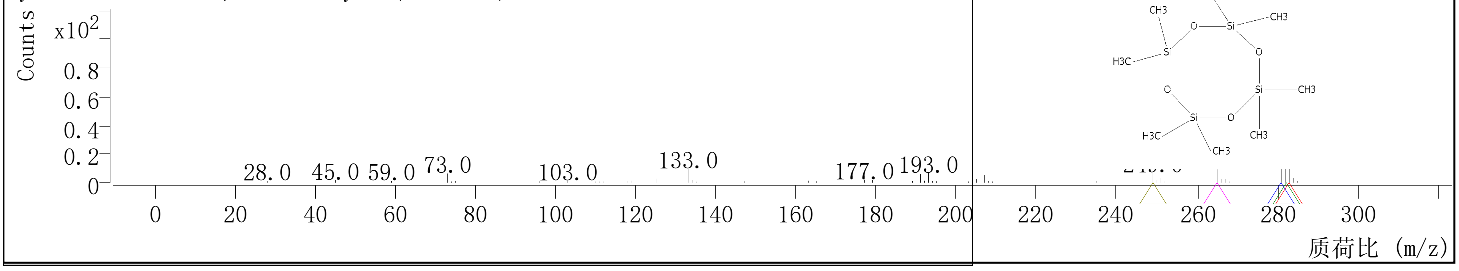

RT	化合物名称	CAS#	分子式	面积	匹配分数	样品	样品
9.0726	Orthoformic acid, triisobutyl ester	16754-49-7	C13H28O3	109467	78.6	0.14	0.59

Orthoformic acid, triisobutyl ester (NIST08.L)

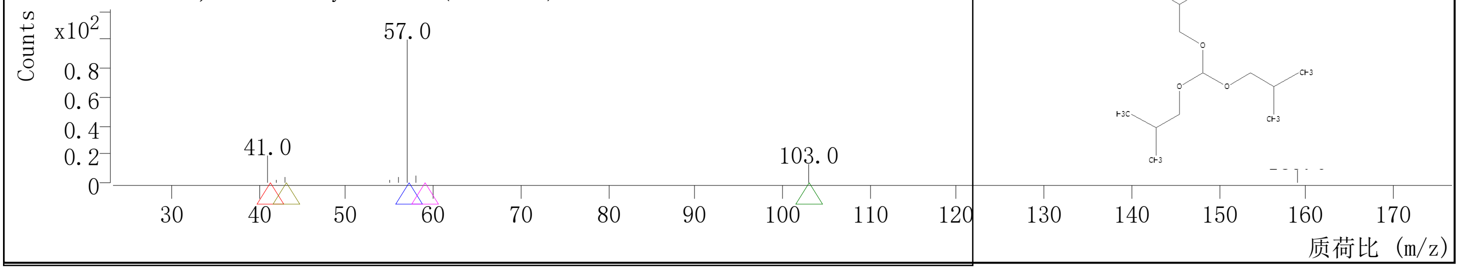

RT	化合物名称	CAS#	分子式	面积	匹配分数	样品	样品
10.2298	Cyclopentasiloxane, decamethyl-	541-02-6	C10H30O5Si5	18466574	98.3	23.97	100.00

Cyclopentasiloxane, decamethyl- (NIST08.L)

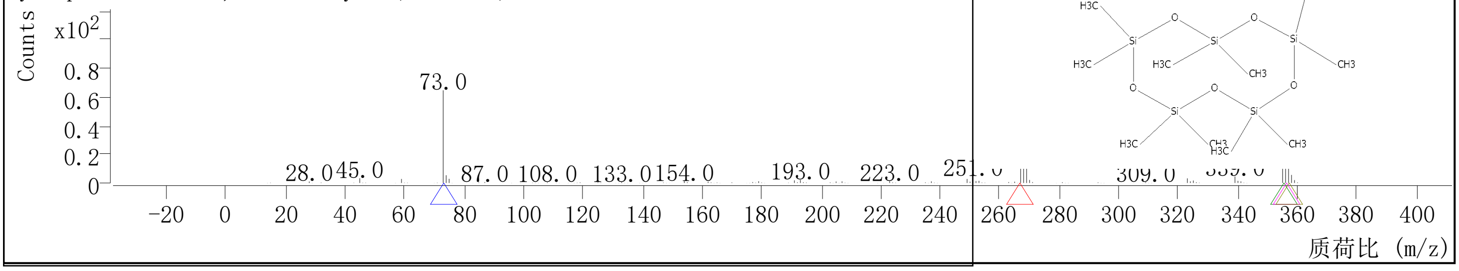

RT	化合物名称	CAS#	分子式	面积	匹配分数	样品	样品
12.6430	dl-Metanephrine, bis(trimethylsilyl) ether	1000334-02-2	C16H31NO3Si2	146087	58.4	0.19	0.79

dl-Metanephrine, bis(trimethylsilyl) ether (NIST08.L)

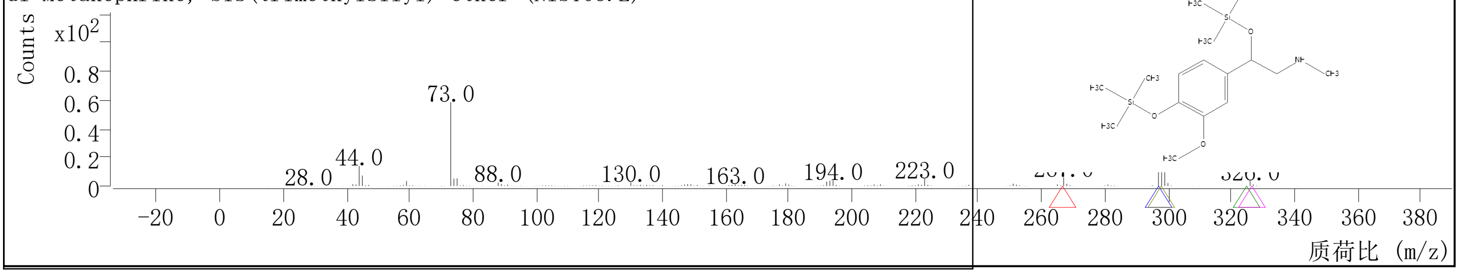

RT	化合物名称	CAS#	分子式	面积	匹配分数	样品	样品
12.7350	3-Pentanol, 3-(1,1-dimethylethyl)-2,2,4,4-tetramethyl-	41902-42-5	C13H28O	652862	78.7	0.85	3.54

3-Pentanol, 3-(1,1-dimethylethyl)-2,2,4,4-tetramethyl- (NIST08.L)

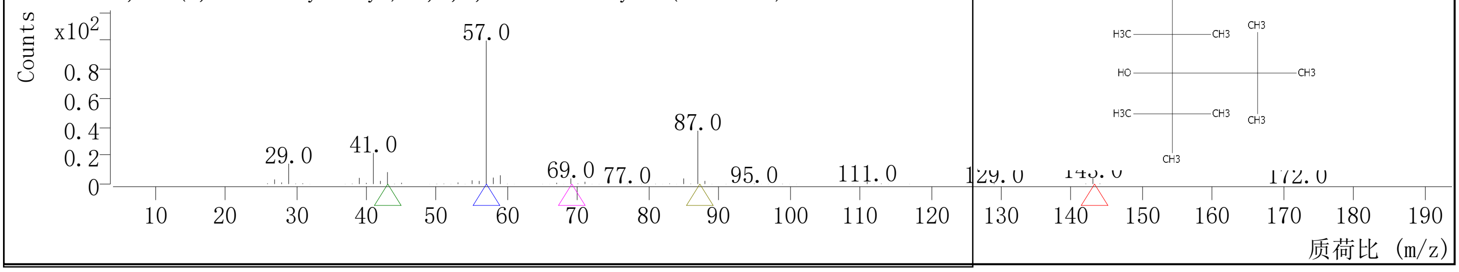

RT	化合物名称	CAS#	分子式	面积	匹配分数	样品	样品
13.5044	2-tert-Butoxytetrahydrofuran	1927-59-9	C8H16O2	117635	79.6	0.15	0.64

2-tert-Butoxytetrahydrofuran (NIST08.L)

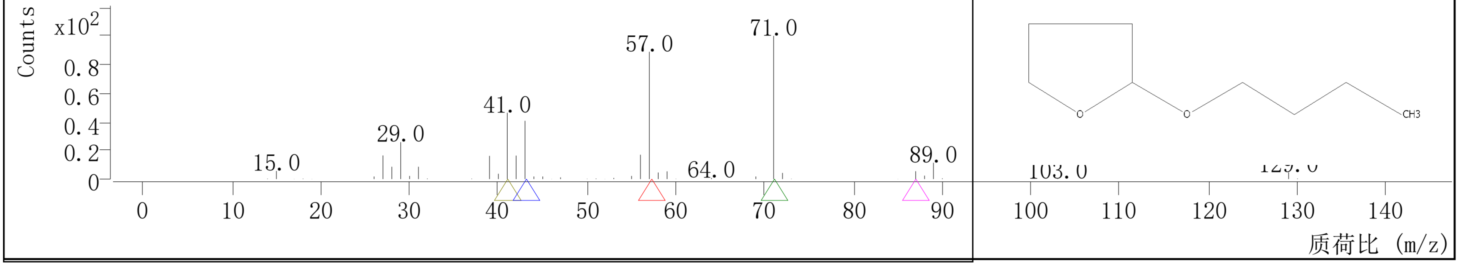

RT	化合物名称	CAS#	分子式	面积	匹配分数	样品	样品
13.7937	Oxalic acid, dodecyl 2-methylphenyl ester	1000309-60-2	C21H32O4	188388	61.5	0.24	1.02

Oxalic acid, dodecyl 2-methylphenyl ester (NIST08.L)

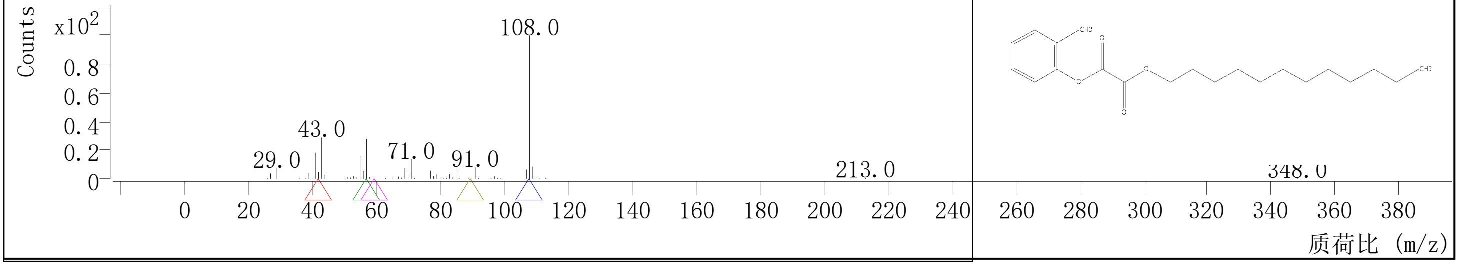

RT	化合物名称	CAS#	分子式	面积	匹配分数	样品	样品
14.2802	Cyclohexasiloxane, dodecamethyl-	540-97-6	C12H36O6Si6	7844008	95.6	10.18	42.48

Cyclohexasiloxane, dodecamethyl- (NIST08.L)

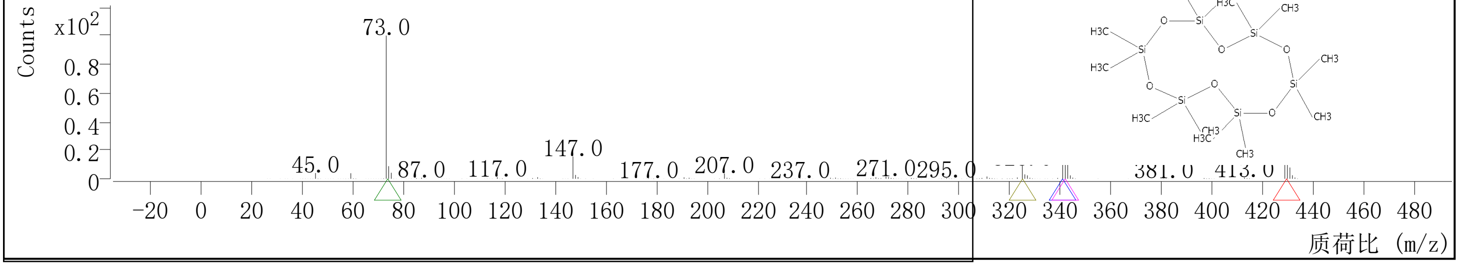

RT	化合物名称	CAS#	分子式	面积	匹配分数	样品	样品
15.1285	2,5-Dimethylhexane-2,5-dihydroperoxide	3025-88-5	C8H18O4	1455811	77.6	1.89	7.88

2,5-Dimethylhexane-2,5-dihydroperoxide (NIST08.L)

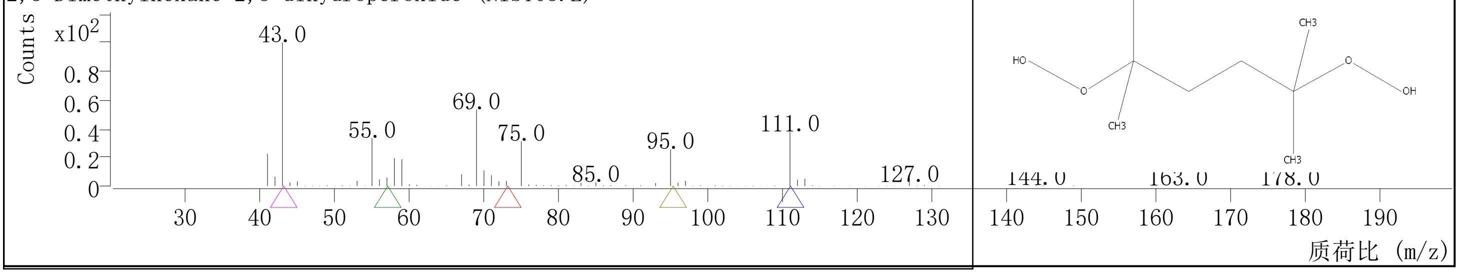

RT	化合物名称	CAS#	分子式	面积	匹配分数	样品	样品
15.4112	2H-Pyranmethanol, tetrahydro-2,5-dimethyl-	54004-46-5	C8H16O2	153233	86.0	0.20	0.83

2H-Pyranmethanol, tetrahydro-2,5-dimethyl- (NIST08.L)

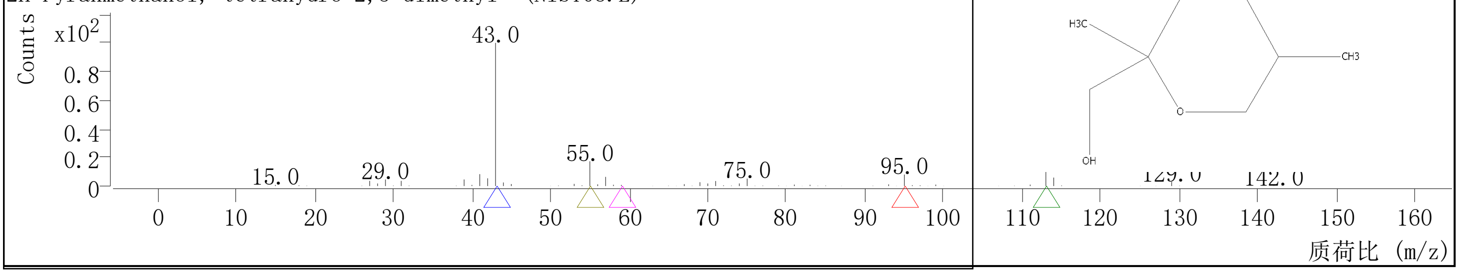

RT	化合物名称	CAS#	分子式	面积	匹配分数	样品	样品
16.1739	Furfural	98-01-1	C5H4O2	136374	92.6	0.18	0.74

Furfural (NIST08.L)

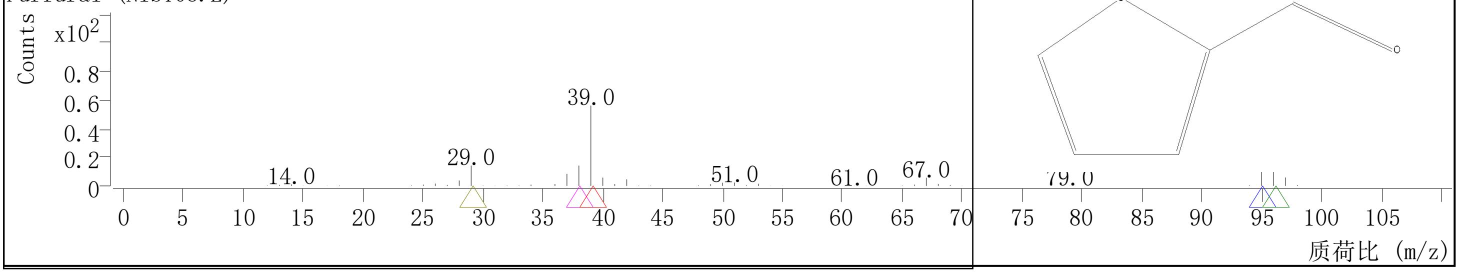

RT	化合物名称	CAS#	分子式	面积	匹配分数	样品	样品
16.4172	2H-Pyranmethanol, tetrahydro-2,5-dimethyl-	54004-46-5	C8H16O2	273070	85.4	0.35	1.48

2H-Pyranmethanol, tetrahydro-2,5-dimethyl- (NIST08.L)

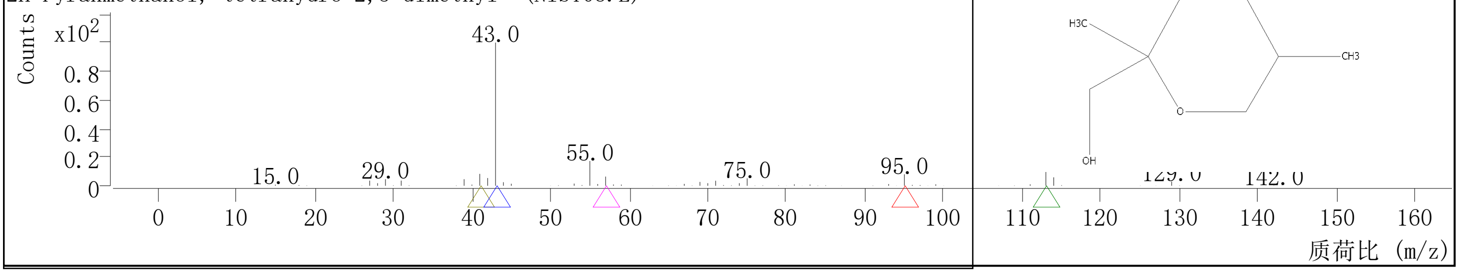

RT	化合物名称	CAS#	分子式	面积	匹配分数	样品	样品
16.5947	Cycloheptasiloxane, tetradecamethyl-	107-50-6	C14H42O7Si7	1583220	97.3	2.05	8.57

Cycloheptasiloxane, tetradecamethyl- (NIST08.L)

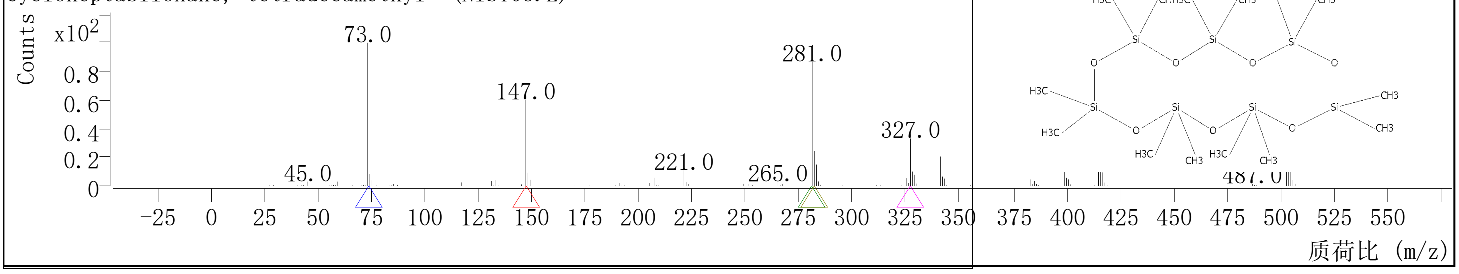

RT	化合物名称	CAS#	分子式	面积	匹配分数	样品	样品
16.7591	Vanillin, tert-butyldimethylsilyl ether	1000352-84-6	C14H22O3Si	306040	76.9	0.40	1.66

Vanillin, tert-butyldimethylsilyl ether (NIST08.L)

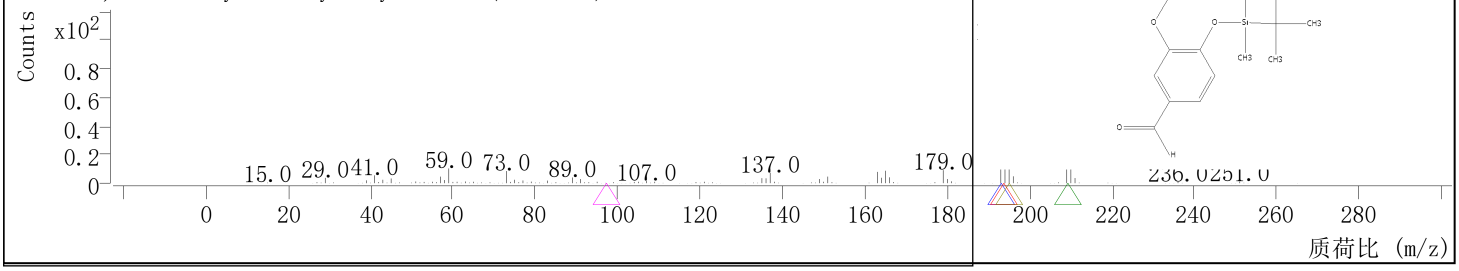

RT	化合物名称	CAS#	分子式	面积	匹配分数	样品	样品
16.9695	Benzaldehyde, 2,5-bis[(trimethylsilyl)oxy]-	56114-69-3	C13H22O3Si2	224171	77.1	0.29	1.21

Benzaldehyde, 2,5-bis[(trimethylsilyl)oxy]- (NIST08.L)

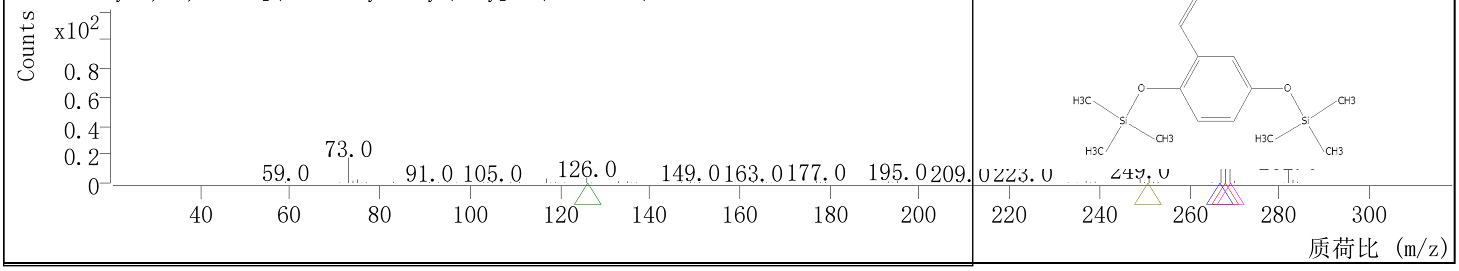

RT	化合物名称	CAS#	分子式	面积	匹配分数	样品	样品
17.0221	Benzaldehyde	100-52-7	C7H6O	1115975	99.0	1.45	6.04

Benzaldehyde (NIST08.L)

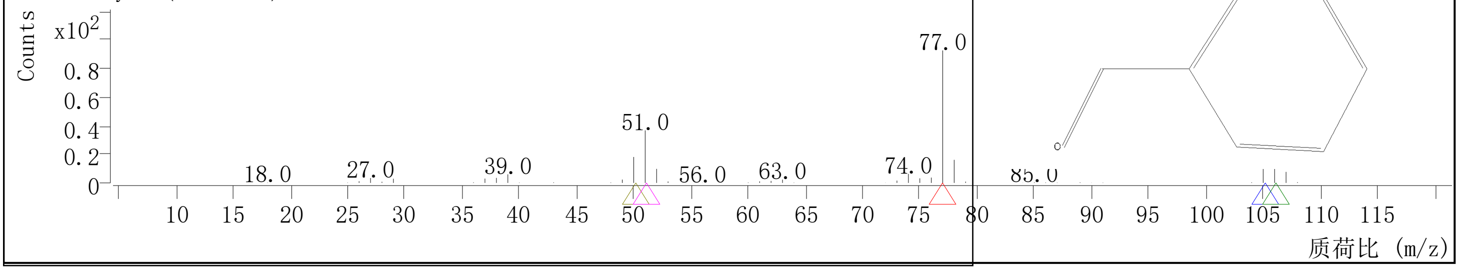

RT	化合物名称	CAS#	分子式	面积	匹配分数	样品	样品
17.2917	2,5-Dimethylhexane-2,5-dihydroperoxide	3025-88-5	C8H18O4	171254	73.7	0.22	0.93

2,5-Dimethylhexane-2,5-dihydroperoxide (NIST08.L)

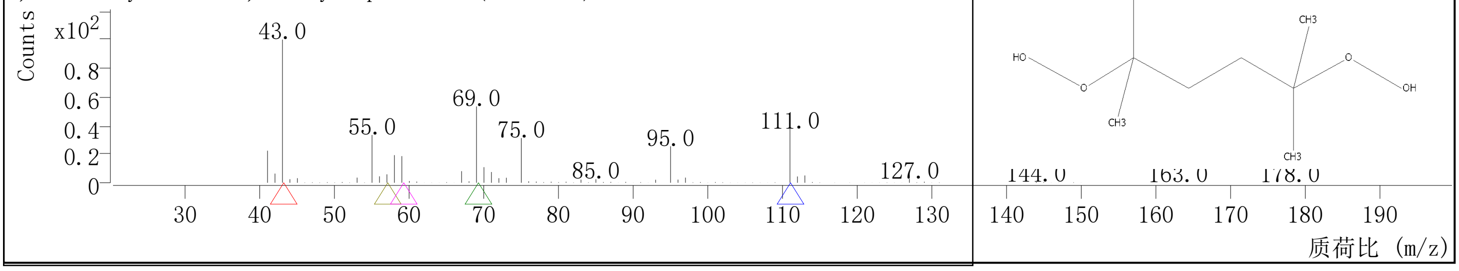

RT	化合物名称	CAS#	分子式	面积	匹配分数	样品	样品
18.3240	Silanediol, dimethyl-	1066-42-8	C2H8O2Si	157878	50.4	0.20	0.85

Silanediol, dimethyl- (NIST08.L)

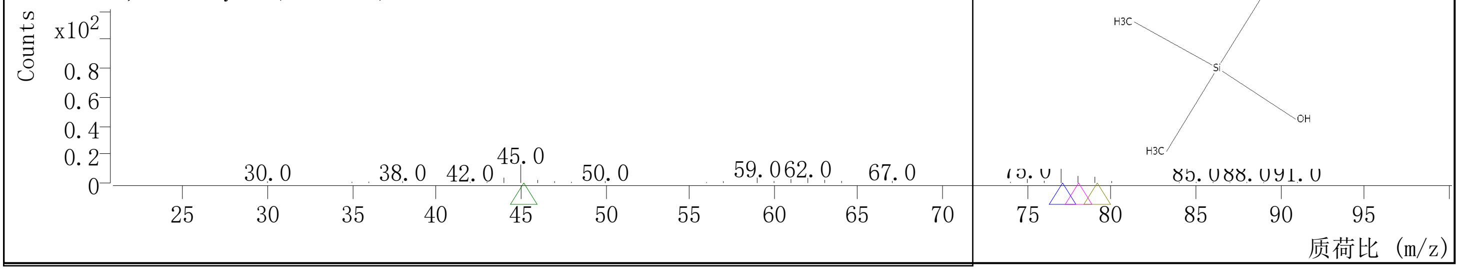

RT	化合物名称	CAS#	分子式	面积	匹配分数	样品	样品
18.3569	Cyclooctasiloxane, hexadecamethyl-	556-68-3	C16H48O8Si8	386211	93.2	0.50	2.09

Cyclooctasiloxane, hexadecamethyl- (NIST08.L)

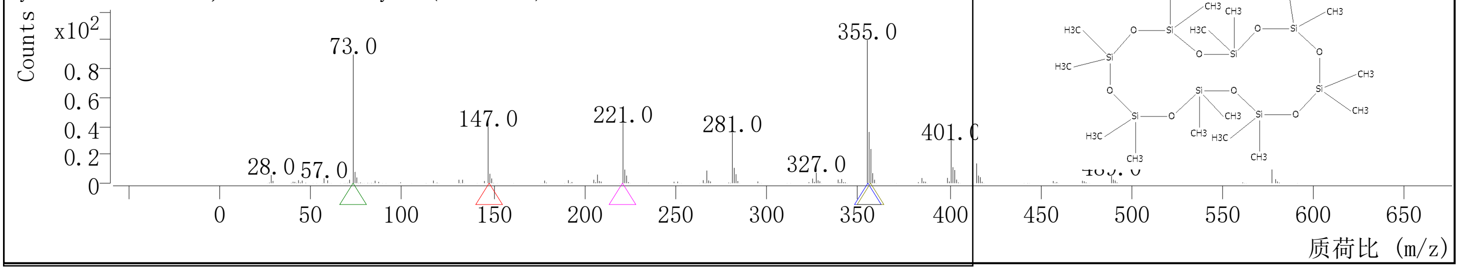

RT	化合物名称	CAS#	分子式	面积	匹配分数	样品	样品
18.4490	Coumarin, 6-benzyloxy-3,4-dihydro-4,4-dimethyl-5-nitro-	1000129-25-7	C18H17NO5	416586	61.3	0.54	2.26

Coumarin, 6-benzyloxy-3,4-dihydro-4,4-dimethyl-5-nitro- (NIST08.L)

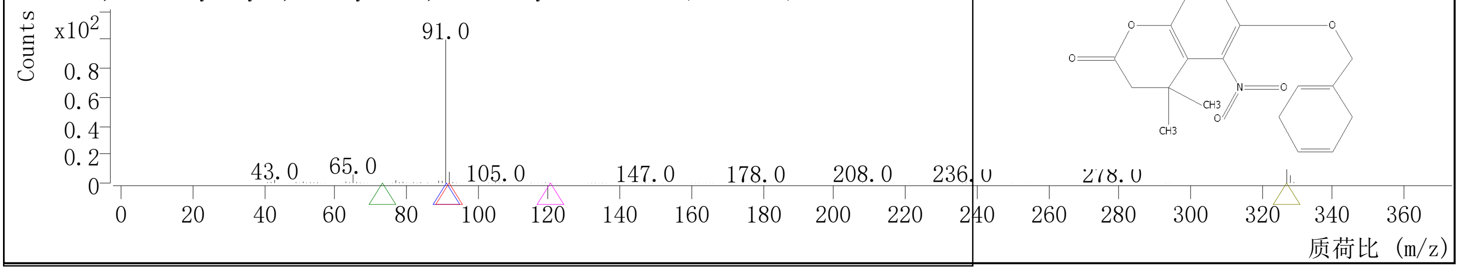

RT	化合物名称	CAS#	分子式	面积	匹配分数	样品	样品
18.5542	2-Furanmethanol	98-00-0	C5H6O2	206061	66.9	0.27	1.12

2-Furanmethanol (NIST08.L)

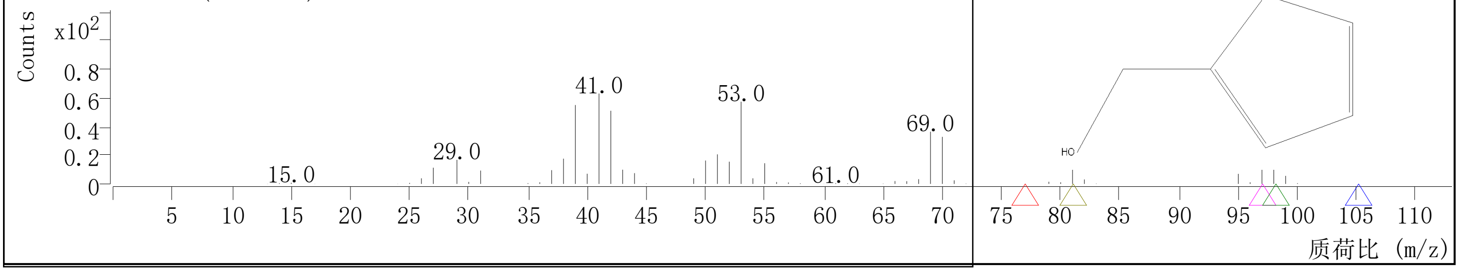

RT	化合物名称	CAS#	分子式	面积	匹配分数	样品	样品
19.2117	Dodecanal	112-54-9	C12H24O	108158	90.6	0.14	0.59

Dodecanal (NIST08.L)

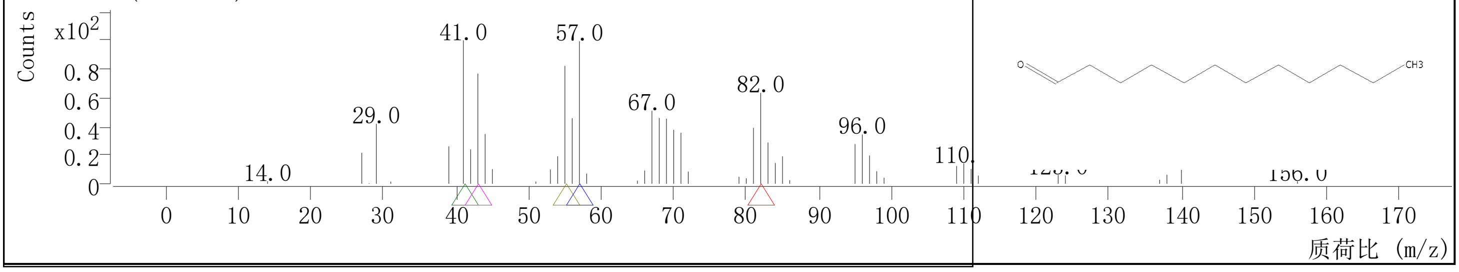

RT	化合物名称	CAS#	分子式	面积	匹配分数	样品	样品
19.4353	Oxime-, methoxy-phenyl-_	1000222-86-6	C8H9NO2	673700	83.1	0.87	3.65

Oxime-, methoxy-phenyl-_ (NIST08.L)

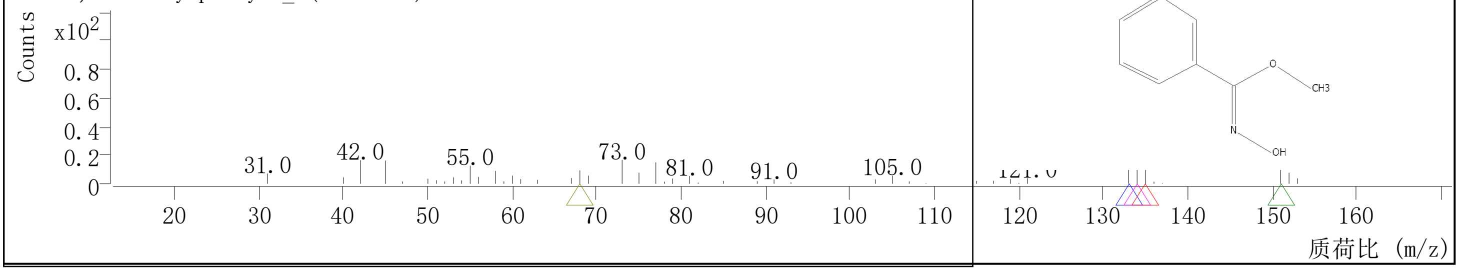

RT	化合物名称	CAS#	分子式	面积	匹配分数	样品	样品
19.5799	Naphthalene	91-20-3	C10H8	127249	92.1	0.17	0.69

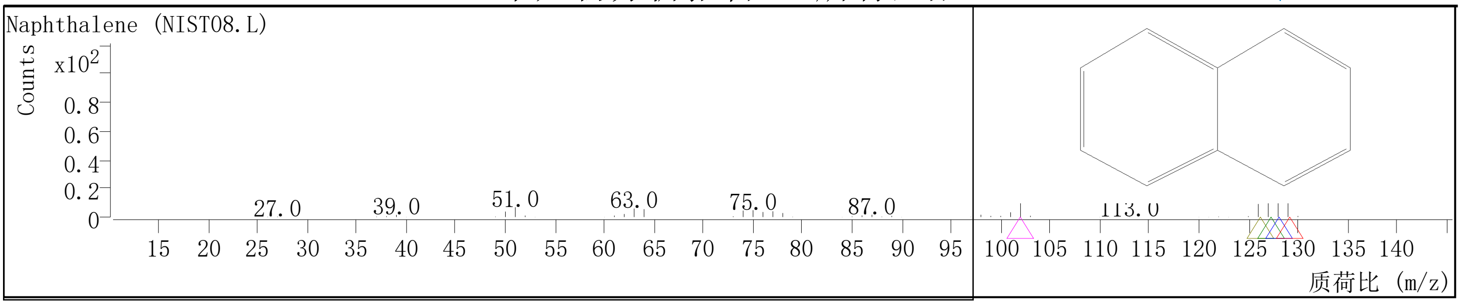

RT	化合物名称	CAS#	分子式	面积	匹配分数	样品	样品
19.6325	Aniline	62-53-3	C6H7N	113899	84.5	0.15	0.62

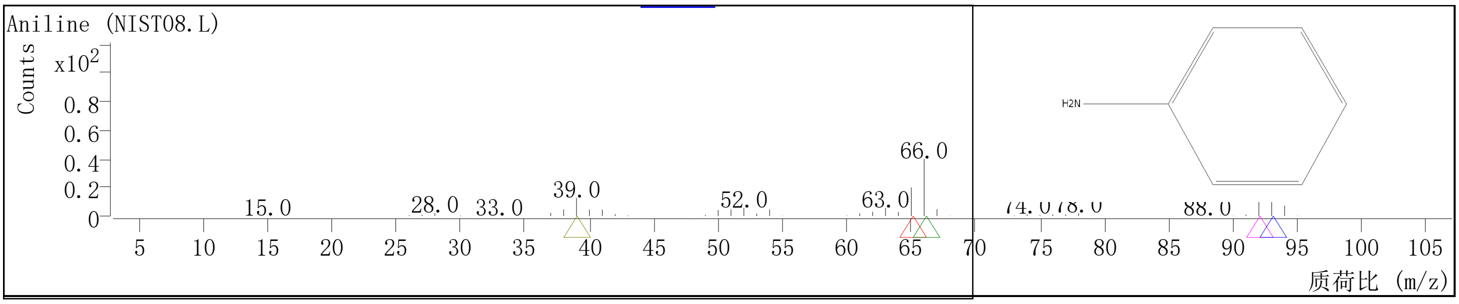

RT	化合物名称	CAS#	分子式	面积	匹配分数	样品	样品
19.8824	Cyclotrisiloxane, hexamethyl-	541-05-9	C6H18O3Si3	292141	91.5	0.38	1.58

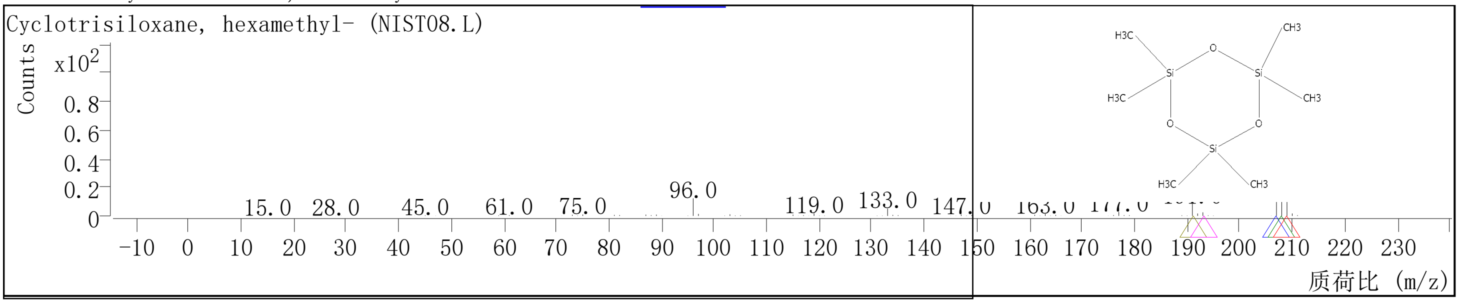

RT	化合物名称	CAS#	分子式	面积	匹配分数	样品	样品
20.2638	Tridecanal	10486-19-8	C13H26O	456710	94.0	0.59	2.47

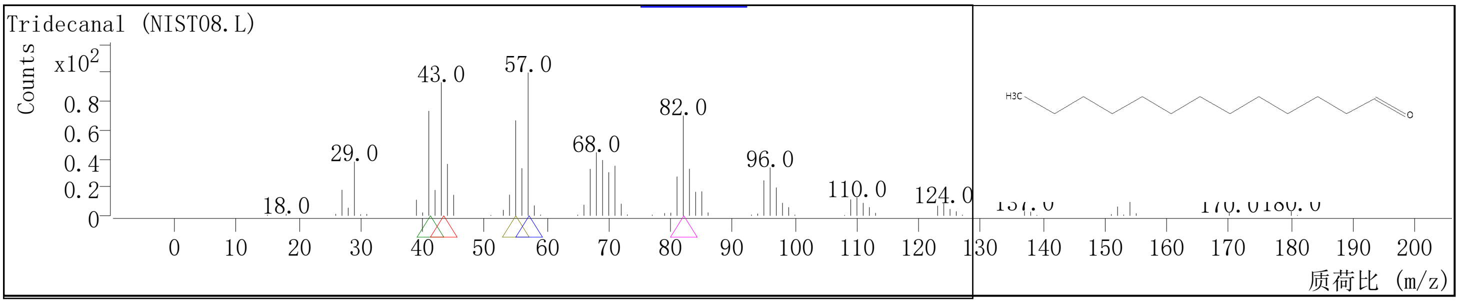

RT	化合物名称	CAS#	分子式	面积	匹配分数	样品	样品
20.4216	Cyclotetrasiloxane, octamethyl-	556-67-2	C8H24O4Si4	151932	93.1	0.20	0.82

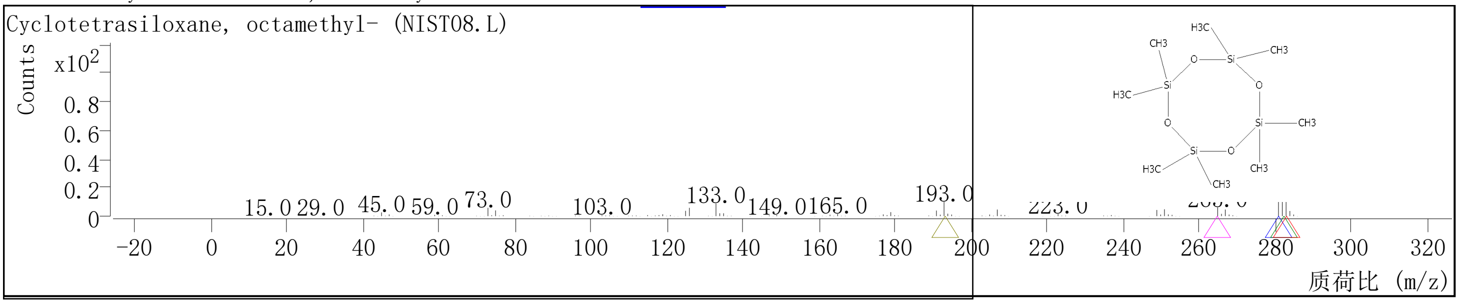

RT	化合物名称	CAS#	分子式	面积	匹配分数	样品	样品
21.0988	Cyclopentasiloxane, decamethyl-	541-02-6	C10H30O5Si5	153387	79.2	0.20	0.83

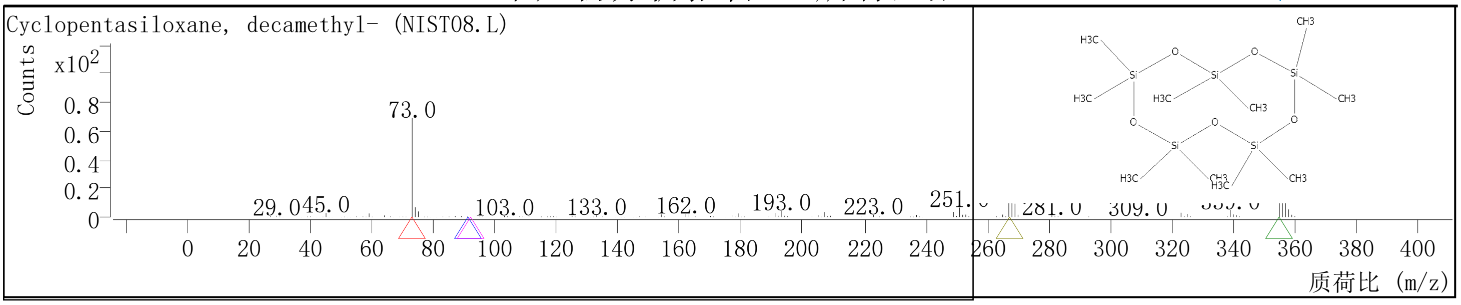

RT	化合物名称	CAS#	分子式	面积	匹配分数	样品	样品
21.2961	Benzyl nitrile	140-29-4	C8H7N	141765	92.2	0.18	0.77

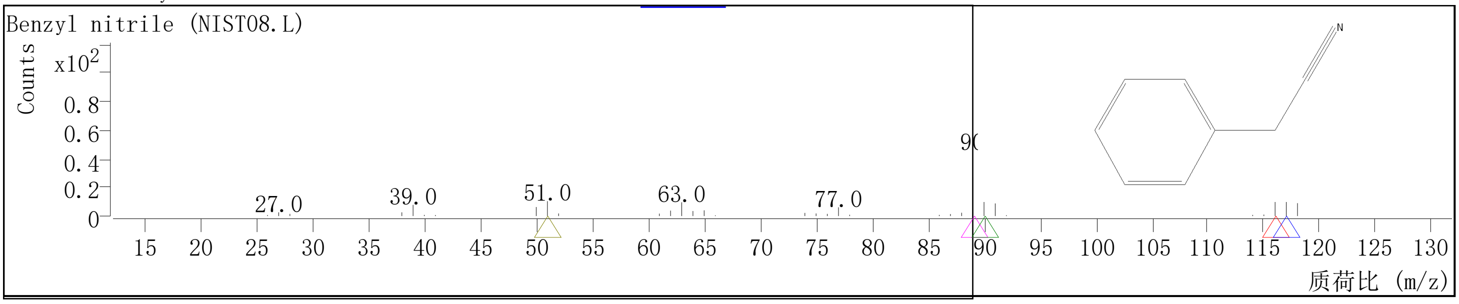

RT	化合物名称	CAS#	分子式	面积	匹配分数	样品	样品
22.1706	Oxirane, hexadecyl-	7390-81-0	C18H36O	210898	82.9	0.27	1.14

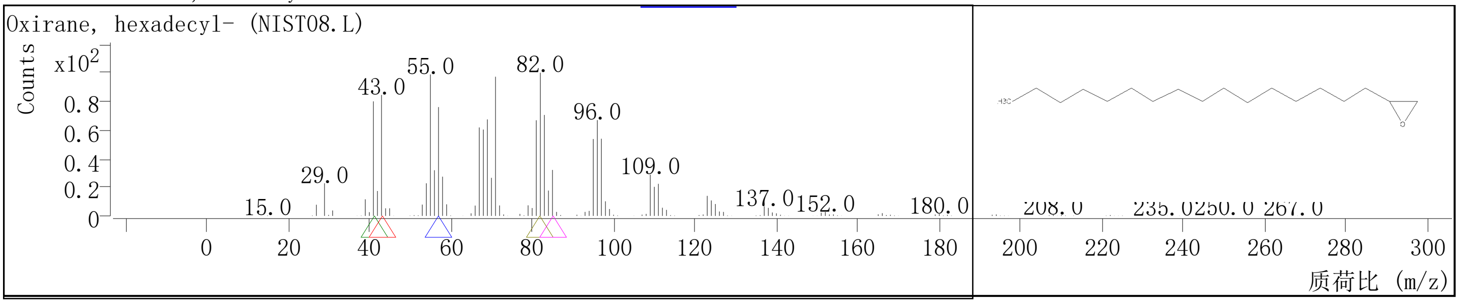

RT	化合物名称	CAS#	分子式	面积	匹配分数	样品	样品
22.3547	Nonanoic acid, 9-oxo-, methyl ester	1931-63-1	C10H18O3	340533	94.5	0.44	1.84

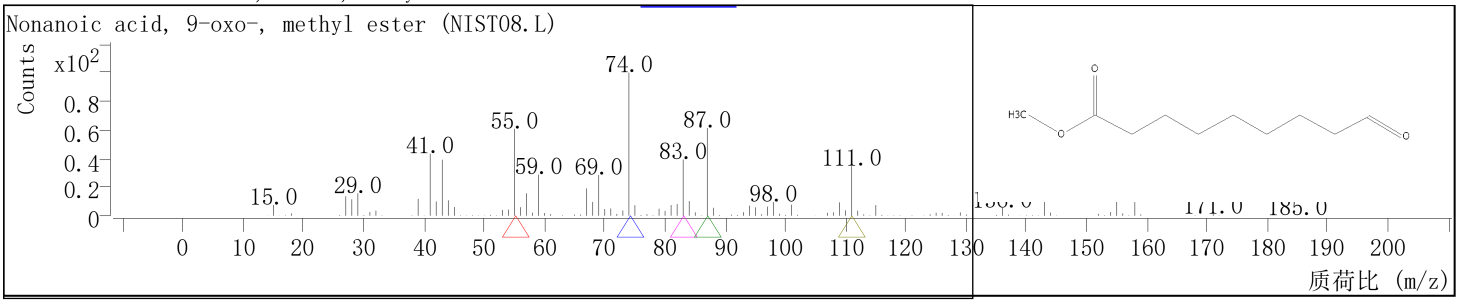

RT	化合物名称	CAS#	分子式	面积	匹配分数	样品	样品
22.4205	N-Acryloylmorpholine	5117-12-4	C7H11NO2	125225	80.6	0.16	0.68

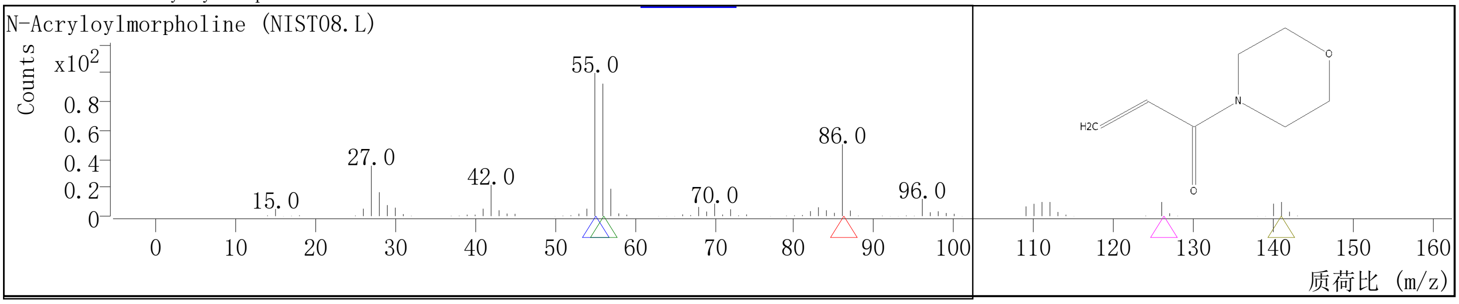

RT	化合物名称	CAS#	分子式	面积	匹配分数	样品	样品
23.7026	Hexadecanoic acid, methyl ester	112-39-0	C17H34O2	3335168	98.5	4.33	18.06

Hexadecanoic acid, methyl ester (NIST08.L)

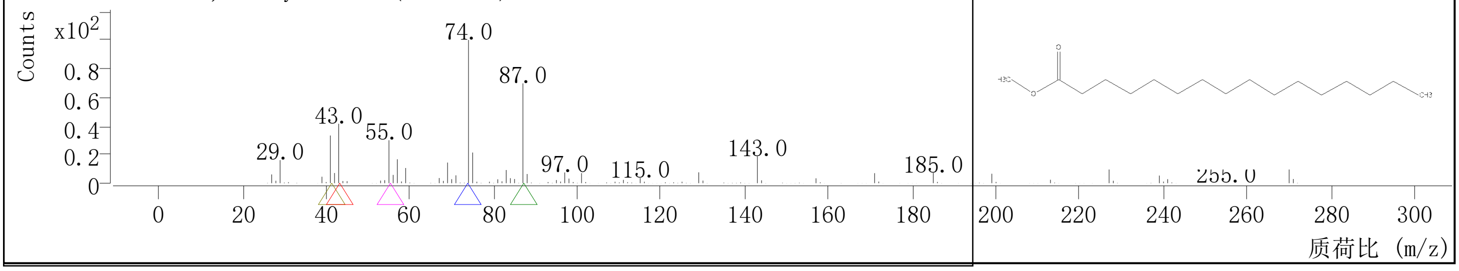

RT	化合物名称	CAS#	分子式	面积	匹配分数	样品	样品
23.9328	9-Hexadecenoic acid, methyl ester, (Z)-	1120-25-8	C17H32O2	139253	87.9	0.18	0.75

9-Hexadecenoic acid, methyl ester, (Z)- (NIST08.L)

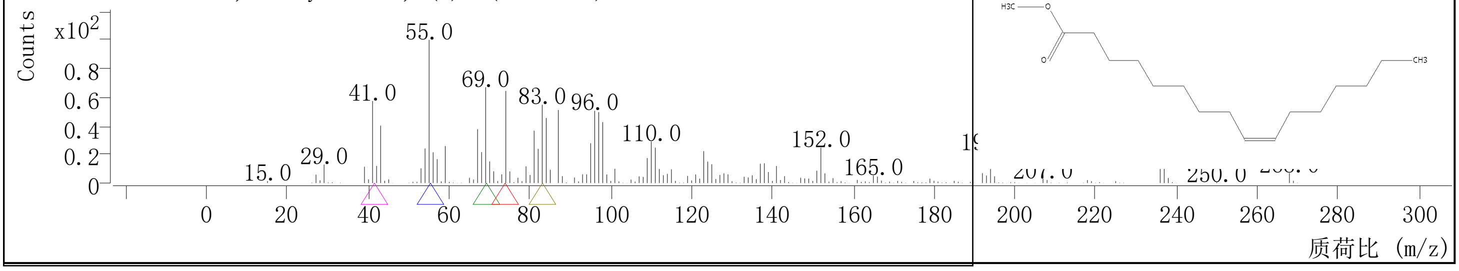

RT	化合物名称	CAS#	分子式	面积	匹配分数	样品	样品
23.9985	Hexadecanoic acid, ethyl ester	628-97-7	C18H36O2	109674	78.5	0.14	0.59

Hexadecanoic acid, ethyl ester (NIST08.L)

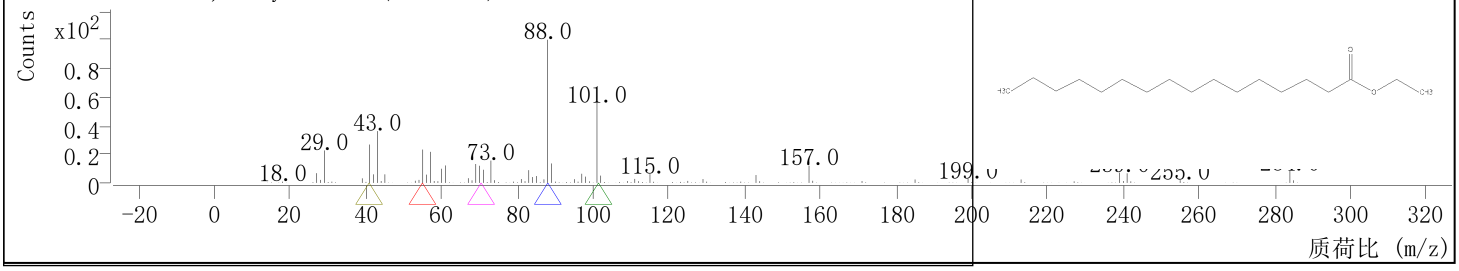

RT	化合物名称	CAS#	分子式	面积	匹配分数	样品	样品
24.3602	Phenol, 2,4-bis(1,1-dimethylethyl)-	96-76-4	C14H22O	159086	86.3	0.21	0.86

Phenol, 2,4-bis(1,1-dimethylethyl)- (NIST08.L)

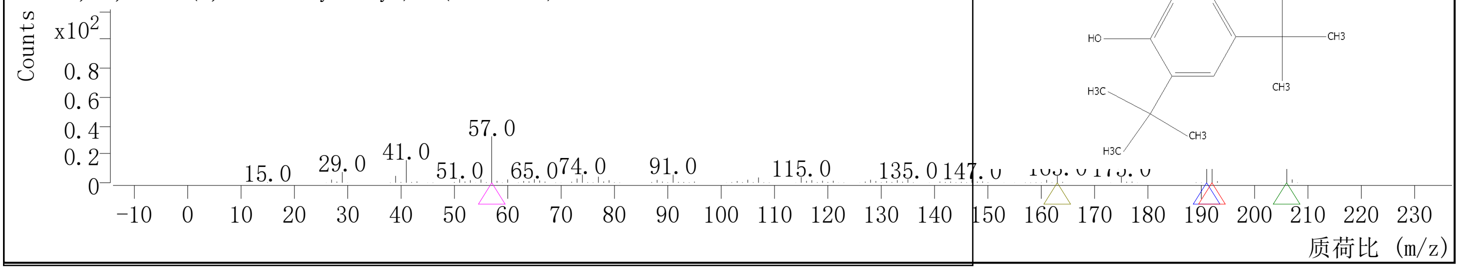

RT	化合物名称	CAS#	分子式	面积	匹配分数	样品	样品
25.5437	Octadecanoic acid, methyl ester	112-61-8	C19H38O2	741379	96.7	0.96	4.01

Octadecanoic acid, methyl ester (NIST08.L)

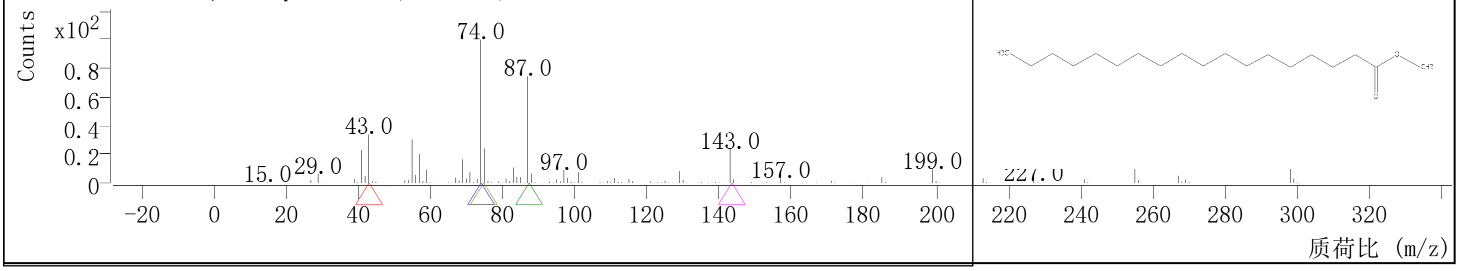

RT	化合物名称	CAS#	分子式	面积	匹配分数	样品	样品
25.7870	9-Octadecenoic acid (Z)-, methyl ester	112-62-9	C19H36O2	2019870	98.9	2.62	10.94

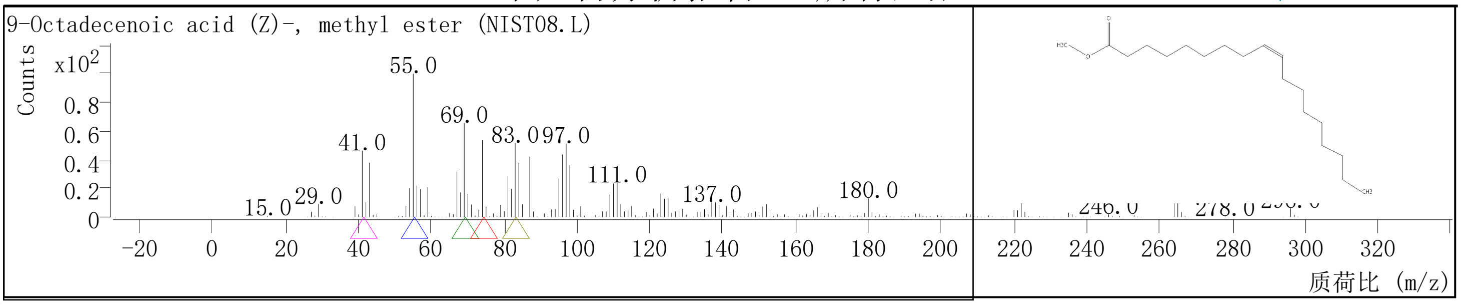

RT	化合物名称	CAS#	分子式	面积	匹配分数	样品	样品
25.8528	trans-13-Octadecenoic acid, methyl ester	1000333-61-3	C19H36O2	111184	67.0	0.14	0.60

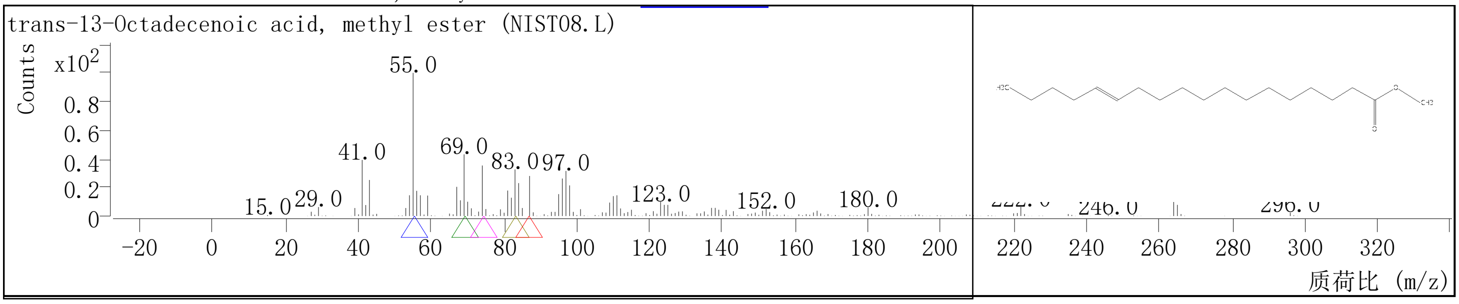

RT	化合物名称	CAS#	分子式	面积	匹配分数	样品	样品
25.9974	Dodecanoic acid	143-07-7	C12H24O2	123260	82.8	0.16	0.67

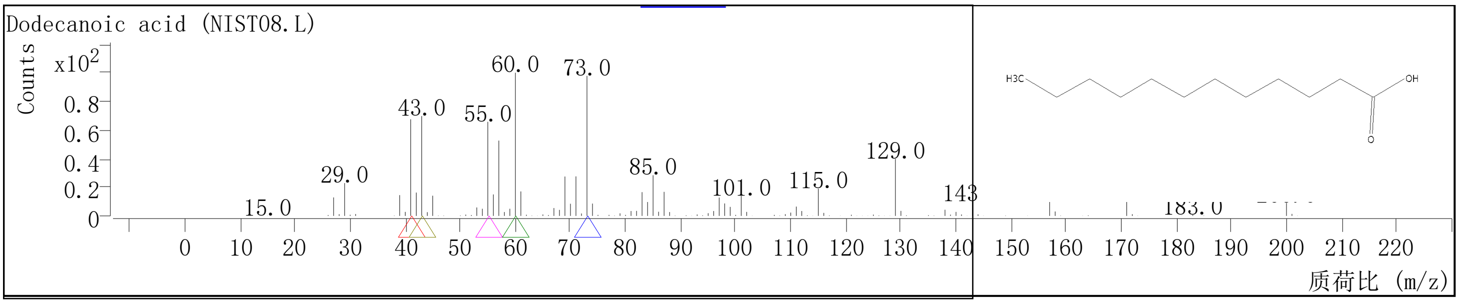

RT	化合物名称	CAS#	分子式	面积	匹配分数	样品	样品
26.3130	9,12-Octadecadienoic acid (Z,Z)-, methyl ester	112-63-0	C19H34O2	372612	91.7	0.48	2.02

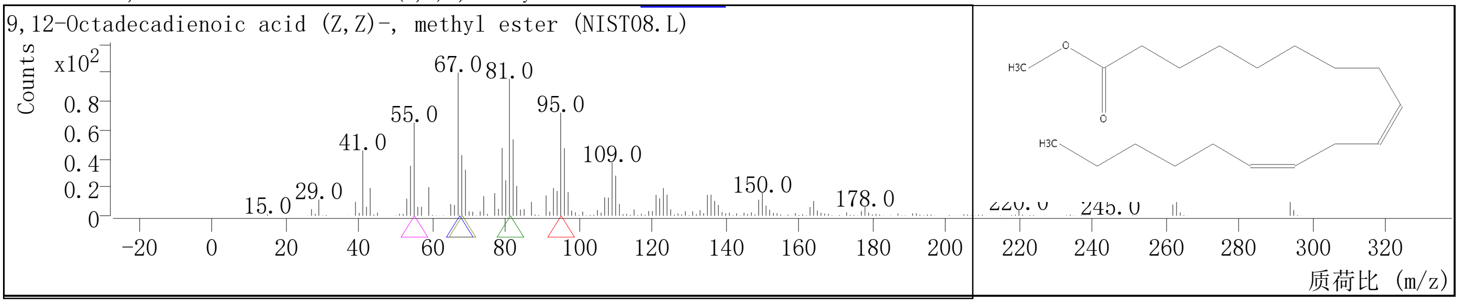

RT	化合物名称	CAS#	分子式	面积	匹配分数	样品	样品
27.3059	E-14-Hexadecenal	330207-53-9	C16H30O	111513	73.8	0.14	0.60

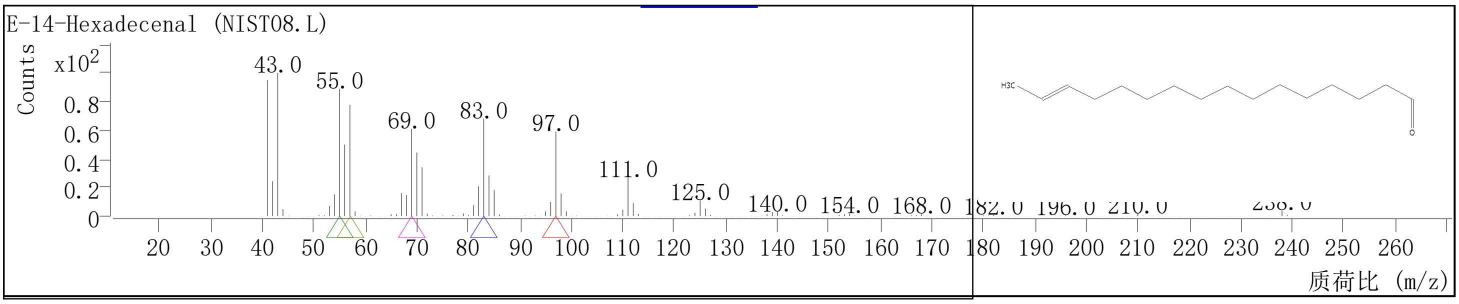

RT	化合物名称	CAS#	分子式	面积	匹配分数	样品	样品
28.0226	Tetradecanoic acid	544-63-8	C14H28O2	311432	86.9	0.40	1.69

Tetradecanoic acid (NIST08.L)

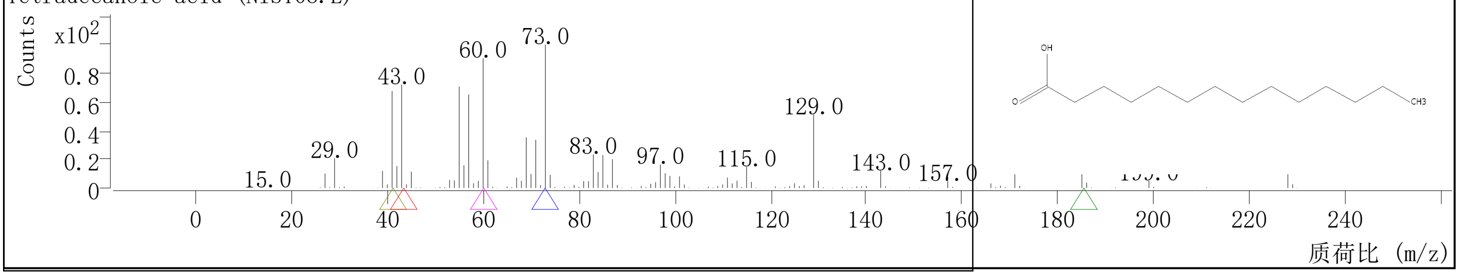

RT	化合物名称	CAS#	分子式	面积	匹配分数	样品	样品
28.8182	Tetradecanoic acid	544-63-8	C14H28O2	1487234	96.0	1.93	8.05

Tetradecanoic acid (NIST08.L)

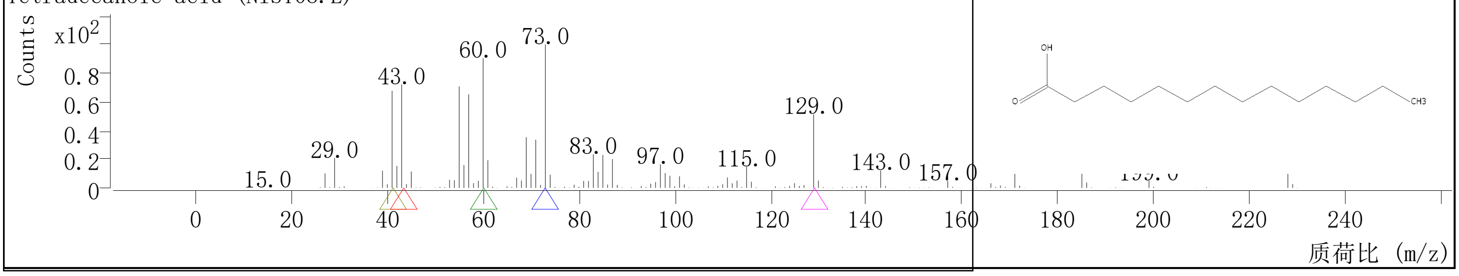

RT	化合物名称	CAS#	分子式	面积	匹配分数	样品	样品
29.0286	Dibutyl phthalate	84-74-2	C16H22O4	109588	63.6	0.14	0.59

Dibutyl phthalate (NIST08.L)

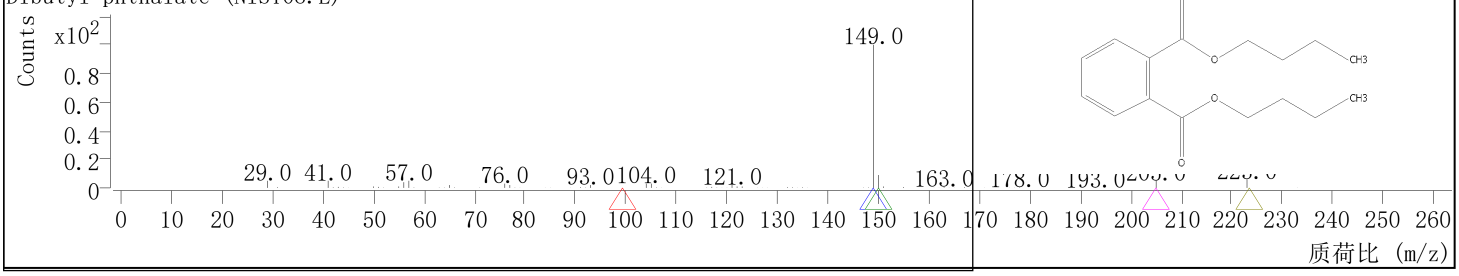

RT	化合物名称	CAS#	分子式	面积	匹配分数	样品	样品
29.5021	Z-7-Tetradecenoic acid	1000130-98-4	C14H26O2	381210	79.5	0.49	2.06

Z-7-Tetradecenoic acid (NIST08.L)

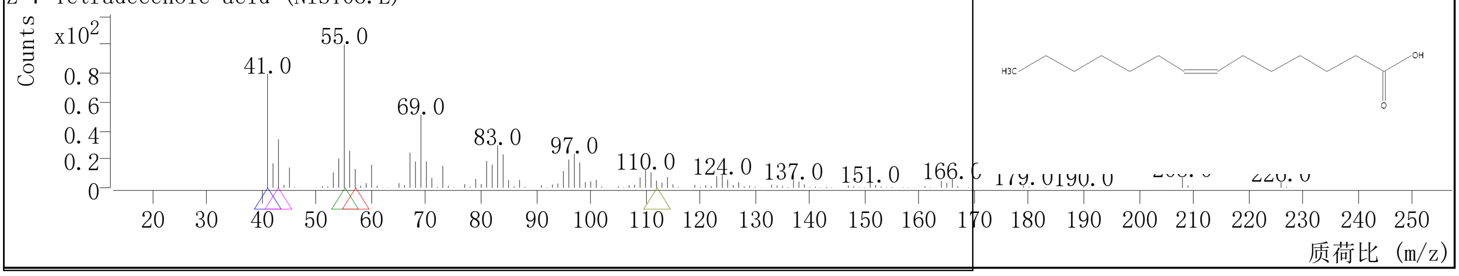

RT	化合物名称	CAS#	分子式	面积	匹配分数	样品	样品
30.1267	Pentadecanoic acid	1002-84-2	C15H30O2	398306	80.8	0.52	2.16

Pentadecanoic acid (NIST08.L)

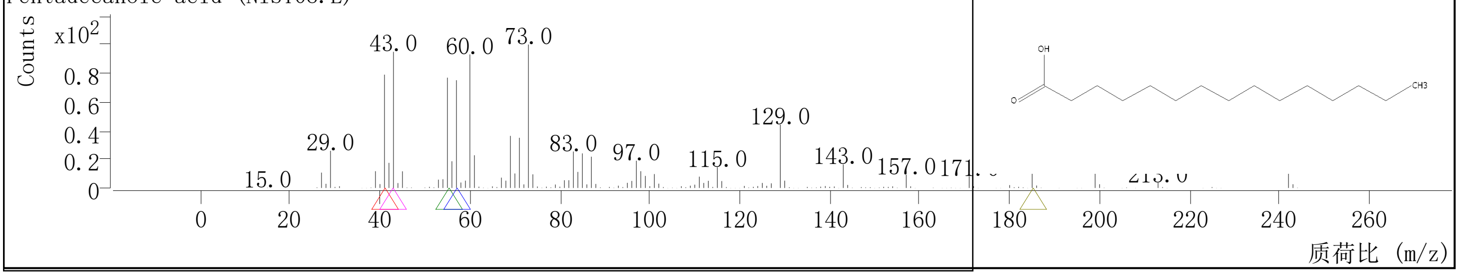

RT	化合物名称	CAS#	分子式	面积	匹配分数	样品	样品
30.7974	Pentadecanoic acid	1002-84-2	C15H30O2	825709	92.2	1.07	4.47

Pentadecanoic acid (NIST08.L)

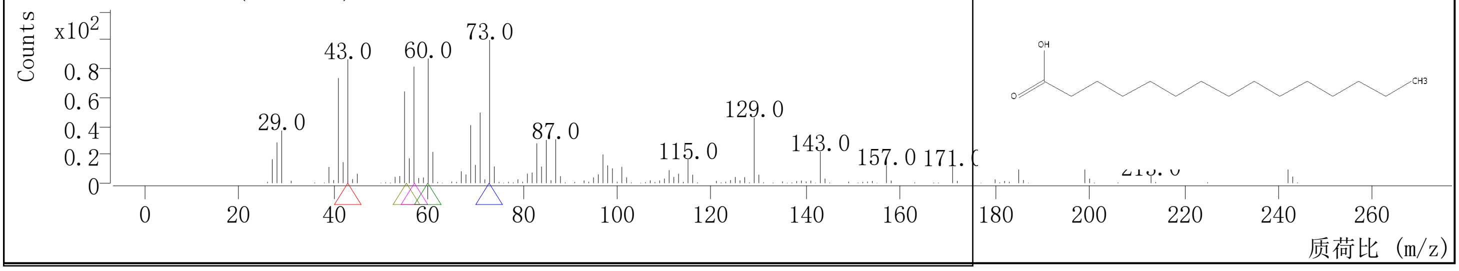

RT	化合物名称	CAS#	分子式	面积	匹配分数	样品	样品
31.6062	Cyclopentadecanone, 2-hydroxy-	4727-18-8	C15H28O2	146674	68.7	0.19	0.79

Cyclopentadecanone, 2-hydroxy- (NIST08.L)

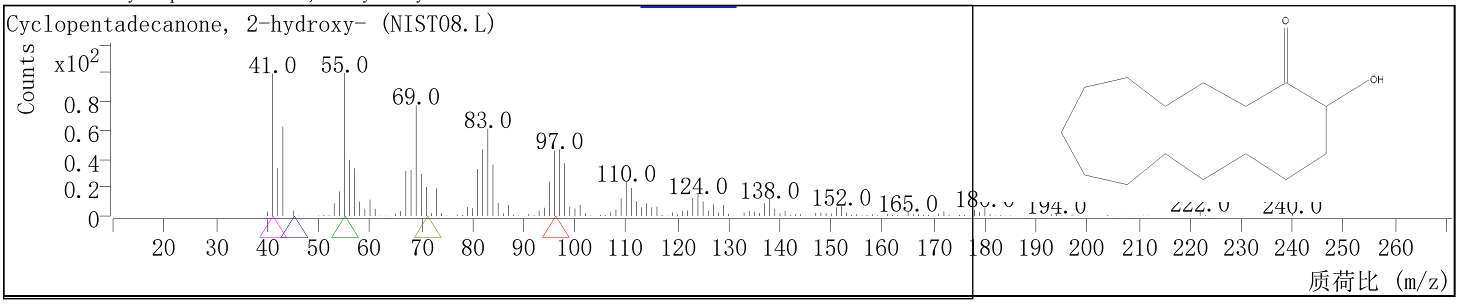

RT	化合物名称	CAS#	分子式	面积	匹配分数	样品	样品
32.0401	n-Hexadecanoic acid	57-10-3	C16H32O2	272028	77.2	0.35	1.47

n-Hexadecanoic acid (NIST08.L)

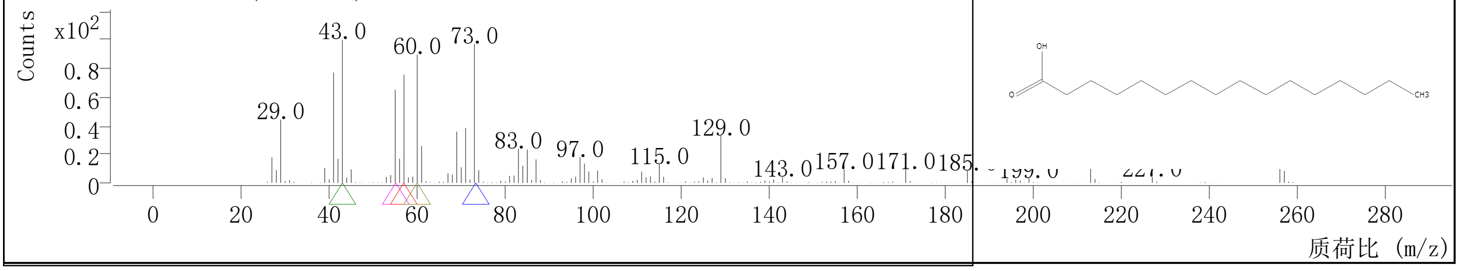

RT	化合物名称	CAS#	分子式	面积	匹配分数	样品	样品
32.9081	cis-9-Hexadecenoic acid	1000333-19-5	C16H30O2	194297	71.7	0.25	1.05

cis-9-Hexadecenoic acid (NIST08.L)

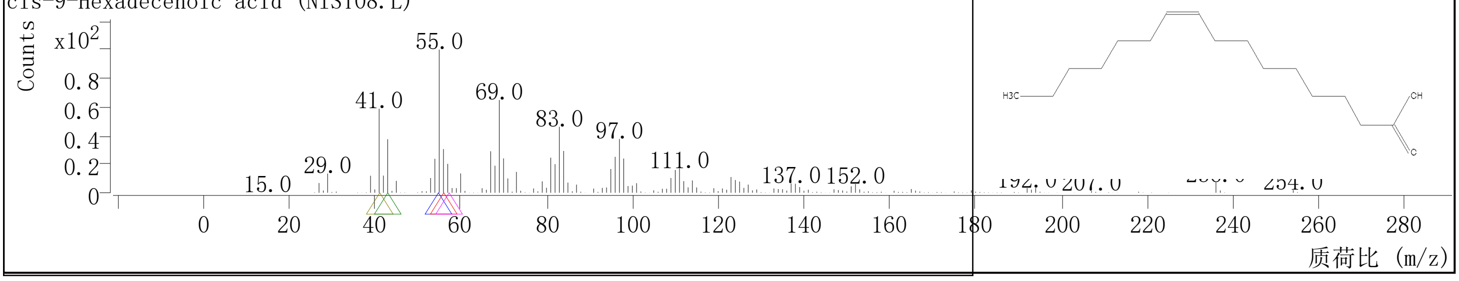

RT	化合物名称	CAS#	分子式	面积	匹配分数	样品	样品
33.3552	n-Hexadecanoic acid	57-10-3	C16H32O2	4787927	96.1	6.21	25.93

n-Hexadecanoic acid (NIST08.L)

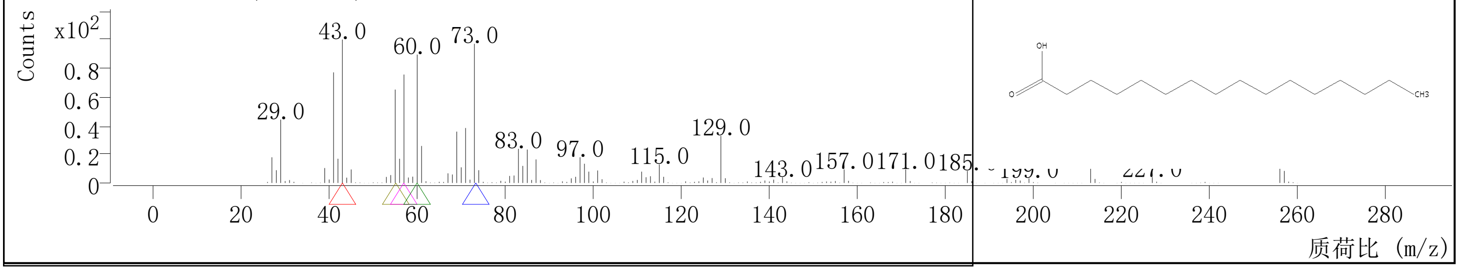

RT	化合物名称	CAS#	分子式	面积	匹配分数	样品	样品
34.3678	cis-9-Hexadecenoic acid	1000333-19-5	C16H30O2	1599687	92.8	2.08	8.66

cis-9-Hexadecenoic acid (NIST08.L)

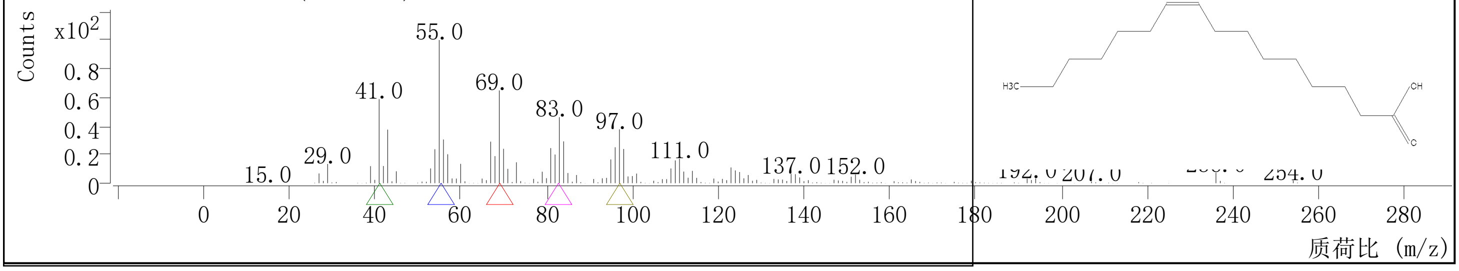

RT	化合物名称	CAS#	分子式	面积	匹配分数	样品	样品
37.8264	.beta.-D-Mannofuranoside, farnesyl-	1000155-15-5	C21H36O6	177528	55.0	0.23	0.96

.beta.-D-Mannofuranoside, farnesyl- (NIST08.L)

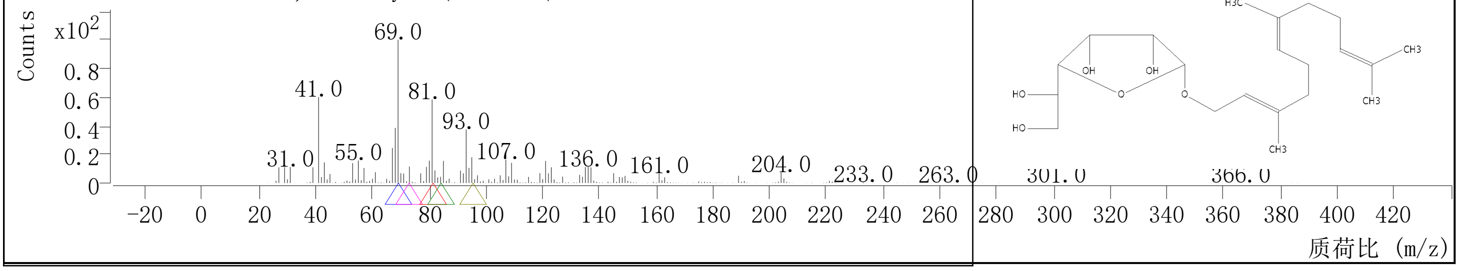

RT	化合物名称	CAS#	分子式	面积	匹配分数	样品	样品
39.3913	Squalene	7683-64-9	C30H50	1409518	90.5	1.83	7.63

Squalene (NIST08.L)

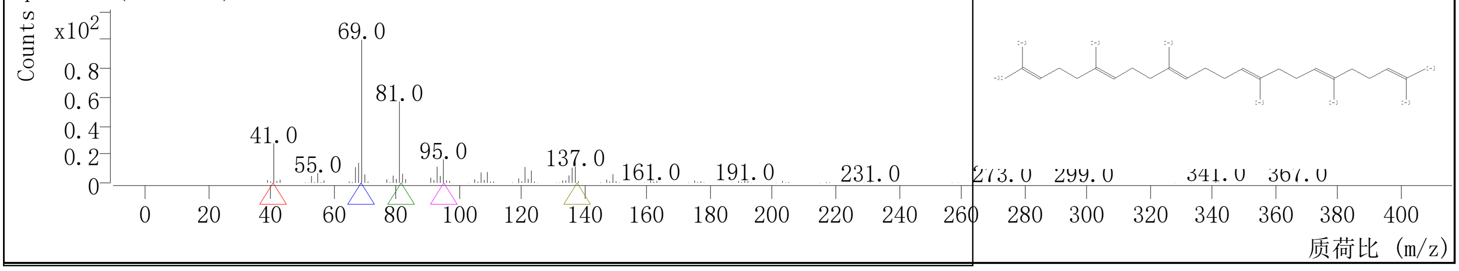

Supplement: Supplementary Chromatogram 2 — Chromatographic results of VOCs produced by LB medium. [file Data_Sheet_2.pdf]
